# Supplementary material for: A comparative analysis of heterogeneity in lung cancer screening effectiveness in two randomised controlled trials
Source: Nat Commun. 2025 Aug 28;16:8060. doi: 10.1038/s41467-025-63471-6 (PMC12394595; doi:10.1038/s41467-025-63471-6)
Supplement: Supplementary file 1 — Supplementary Information [file 41467_2025_63471_MOESM1_ESM.pdf]

Supplementary information for:

A comparative analysis of heterogeneity in lung cancer screening effectiveness in two randomised controlled trials

Max Welz MSc[1,2]†, Carlijn M. van der Aalst PhD[1], Andreas Alfons PhD[2], Andrea A. Naghi PhD[2,3], Marjolein A. Heuvelmans M.D., PhD[4,5,6], Harry J.M. Groen M.D., PhD[7], Pim A. de Jong M.D., PhD[8], Joachim Aerts M.D., PhD[9], Matthijs Oudkerk M.D., PhD[5], Harry J. de Koning M.D., PhD*[1], Kevin ten Haaf PhD*†[1], on behalf of the NELSON trial consortium

Statistical Methodology Supplementary Appendix

1 General Comments

In this document we provide a technical description of the methods used in the main paper and how they were implemented. The methods are traditional "one-variable-at-a-time-analysis", risk models, effect models, and causal random forests[1]. Risk models and effect models are recommended for the evaluation of treatment effect heterogeneity by recent clinical guidelines in the *predictive approaches to treatment effect heterogeneity* (PATH) statement[2], which is why we use them. In general, we use every method in accordance with the PATH statement.

2 Notation

Let $\{(X_i, Y_i, W_i)\}_{i=1}^n$ be a random sample of size n with the following characteristics. For individuals $i = 1, \ldots, n$, we assume that $X_i = (X_{1,i}, \ldots, X_{d,i})^\top$ is a d-dimensional real-valued random vector of explanatory variables that are measured before some treatment intervention (in this case, screening for lung cancer with computed tomography), Y_i is a binary response variable, and W_i is a binary treatment assignment variable. We say that individual i *has the event* (that is, lung cancer death here[i]) if $Y_i = 1$ and that i has been treated if $W_i = 1$. We call all individuals who have been screened the *treatment* group and all individuals who have not been screened the *control* group. We are interested in the causal effect of W_i on Y_i and potential heterogeneity therein. Using the well-known Rubin causal model, which defined the potential outcomes be defined as $Y_i(1)$ and $Y_i(0)$, denoting the outcome if individual i is treated and if it is not treated, respectively. We only ever observe one of the potential outcomes for a given i, that is, $Y_i = Y_i(W_i)$. The conditional average treatment effect (CATE) of individual i at an arbitrary covariate realization $X_i = x$ is formally defined as

$$\mathsf{CATE}(x) = \mathbb{E}[Y_i(1) - Y_i(0) \mid X_i = x] = \mathbb{P}[Y_i(1) = 1 \mid X_i = x] - \mathbb{P}[Y_i(0) = 1 \mid X_i = x]. \quad (1)$$

[i] The observation periods in which lung cancer deaths are observed as follows. In NELSON, the observation period is between randomization and December 31, 2015, or 10 years of follow-up since randomization (whichever came first). In NLST, it is between randomization and 7 years of follow-up.

We are interested in estimating the treatment effect function $x \mapsto \mathsf{CATE}(x)$ on at least a group level, based on the observations $\{(X_i, Y_i, W_i)\}_{i=1}^n$. We frequently call this quantity the *absolute benefit* (of the treatment). Another quantity of interest is the relative conditional average relative treatment effect,

$$\mathsf{RCATE}(x) = \frac{\mathbb{E}[Y_i(1)|X_i = x]}{\mathbb{E}[Y_i(0)|X_i = x]} = \frac{\mathbb{P}[Y_i(1) = 1 \mid X_i = x]}{\mathbb{P}[Y_i(0) = 1 \mid X_i = x]} - 1, \tag{2}$$

which is often called the *relative benefit* (of the treatment). The value 1 is deducted for interpretational consistency with the CATE, in the sense that if a treatment is effective in reducing mortality, both CATE and RCATE will be negative.

At $X_i = x_i$, we introduce the shorthand notation $\mathsf{CATE}_i = \mathsf{CATE}(x_i)$ and $\mathsf{RCATE}_i = \mathsf{RCATE}(x_i)$, for individuals $i = 1, \dots, n$.

3 One-Variable-at-a-Time Analyses

One-variable-at-a-time analyses are typically by means of a series of exact rate ratio tests. Suppose the n individuals at hand can be partitioned into two disjoint groups, $\mathcal{G}_0 \subset \{1, \dots, n\}$ and $\mathcal{G}_1 \subset \{1, \dots, n\}$, and these groups are not necessarily collectively exhaustive. The partitioning is usually done according to treatment assignment along some characteristics of a single explanatory variable, hence the name *one-variable-at-a-time analysis.* For example, $\mathcal{G}_0$ and $\mathcal{G}_1$ may contain the younger-than-60-years-old individuals in the control group and treatment group, respectively.

The random variables

$$P_0 = |\{i \in \mathcal{G}_0 : Y_i = 1\}| \quad \text{and} \quad P_1 = |\{i \in \mathcal{G}_1 : Y_i = 1\}|$$

count the number of events in each group. We furthermore assume that we observe a possibly right-censored time-at-risk of each individual i, denoted T_i. Let

$$N_0 = \sum_{\{i : i \in \mathcal{G}_0\}} T_i \quad \text{and} \quad N_1 = \sum_{\{i : i \in \mathcal{G}_1\}} T_i$$

denote the cumulative time at risk within each group. We assume that the random variables P_0, P_1 that measure number of events in each group are Poisson-distributed as

$$P_0 \sim \mathsf{Poisson}(N_0 \lambda_0) \quad \text{and} \quad P_1 \sim \mathsf{Poisson}(N_1 \lambda_1),$$

where the parameters $\lambda_0, \lambda_1 > 0$ are unknown. This assumption implies that we can interpret the cumulative time at risk in each group, N_0 and N_1, as the total time spent in Poisson processes P_0 and P_1, respectively. We are interested in the *rate ratio* RR, defined by

$$\mathsf{RR} = \frac{\lambda_0}{\lambda_1}.$$

Typically, a uniformly most powerful (UMP) test[3] is used for testing hypotheses on the rate ratio RR. In our case, such tests are based on the observation that the conditional random variable

$$P_0 \mid P_0 + P_1$$

follows a Binomial distribution with $P_0 + P_1$ trials and probability parameter

$$\pi(\mathsf{RR}) = \frac{N_0\lambda_0}{N_0\lambda_0 + N_1\lambda_1} = \frac{N_0\mathsf{RR}}{N_0\mathsf{RR} + N_1},$$

which is a function of the rate ratio RR.

We exploit the facts that we know the distribution of $P_0 \mid P_0 + P_1$ and that $\pi(\mathsf{RR})$ is a continuous and increasing function of the parameter of interest, RR, to perform inference on RR. For instance, suppose that we are interested in testing the null hypothesis, H_0, that the unknown true rate ratio RR is equal to some fixed value $c > 0$, against a two-sided alternative hypothesis, H_a. Formally, we test

$$H_0 : \mathsf{RR} = c \quad \text{vs.} \qquad H_a : \mathsf{RR} \neq c$$

and the associated UMP test can be shown to have a p-value of

$$2 \cdot \min\Big\{\mathbb{P}[B_c \leq P_0 \mid P_0 + P_1],\ \mathbb{P}[B_c \geq P_0 \mid P_0 + P_1]\Big\},$$

where $B_c|P_0 + P_1 \sim \mathsf{Binomial}\big(P_0 + P_1, \pi(c)\big)$.

Tests on the rate ratio RR are typically used as follows. Suppose that the groups $\mathcal{G}_0$ and $\mathcal{G}_1$ contain some subgroup of interest in the treatment and control group, respectively. If we test the null hypothesis $H_0 : \mathsf{RR} = 1$ and reject it at an appropriate level, we have found evidence that there seem to be systematic differences in the occurrence of events between the two groups, and this difference is possibly due to the treatment intervention.

4 Predictive Models

In the following, we describe the predictive modeling techniques as suggested in the PATH statement[2]. These predictive models can be broken down into *risk models* and *effect models*. The goal of these predictive models is to estimate the absolute and relative benefit in equations (1) and (2), respectively.

4.1 Risk Models

The idea behind risk models is to separately estimate the effect of X_i on Y_i and the effect of W_i on Y_i, for individuals $i = 1, \ldots, n$. When doing so, one separates the explanatory power for Y_i into a part that is due to X_i and a part that is due to W_i. This gives rise to the following two-stage estimation procedure.

4.1.1 Stage 1: Baseline Risk

In the first stage, one fits a linear logistic regression model of Y_i on X_i, which assumes the linear identity

$$\ln\left(\frac{\mathbb{P}[Y_i = 1|X_i = x_i]}{1 - \mathbb{P}[Y_i = 1|X_i = x_i]}\right) = \beta_0 + x_i^\top \beta, \tag{3}$$

for all $i = 1, \ldots, n$, where $(\beta_0, \beta) = (\beta_0, \beta_1, \ldots, \beta_p) \in \mathbb{R}^{d+1}$ is some fixed but unknown vector of coefficients. The left hand side of (3) is a log odds ratio. Rearranging model (3) yields the following useful identity for the conditional probabilities of having the event,

$$\begin{aligned}\mathbb{P}[Y_i = 1|X_i = x_i] &= \Big(1 + \exp\big(-\mathsf{LP}_i\big)\Big)^{-1} \\ &= F_{\text{logistic}}\big(\mathsf{LP}_i\big),\end{aligned} \tag{4}$$

where

$$\mathsf{LP}_i = \beta_0 + x_i^\top \beta \tag{5}$$

is said to be a *linear predictor* and F_{logistic} is the distribution function of the logistic distribution with location zero and scale one.

It is recommended to fit the model (3) by means of regularized logistic regression, which is sometimes called penalized logistic regression[2]. In our paper, we follow this recommendation and use a cross-validated elastic-net type regularization term ([4], see[5] for numerical details).[ii] We refer to Appendix A for a description of regularized logistic regression. Fitting model (3) by means of regularized logistic regression yields estimates $(\widehat{\beta}_0, \widehat{\beta})$ of the coefficients (β_0, β). The regularization has the effect that these estimates are shrunk, which means that covariates with low explanatory power for the outcome will have an estimated coefficient whose value is close to zero, possibly exactly zero. If an estimated coefficient vector $\widehat{\beta}$ has many zeros, it is said to be *sparse*.

The estimates $(\widehat{\beta}_0, \widehat{\beta})$ of the coefficients (β_0, β) in model (3) can then be used to compute an estimated linear predictor, $\widehat{\mathsf{LP}}_i$, by evaluating the linear predictor in (5) at the estimates, yielding

$$\widehat{\mathsf{LP}}_i = \widehat{\beta}_0 + x_i^\top \widehat{\beta}.$$

With this estimated linear predictor, we can obtain estimates of the baseline risk, $\widehat{\mathbb{P}}[Y_i = 1|X_i = x_i]$, by plugging it into (4), resulting in baseline risk estimates

$$\widehat{p}_i = \widehat{\mathbb{P}}[Y_i = 1|X_i = x_i] = F_{\text{logistic}}\Big(\widehat{\mathsf{LP}}_i\Big). \tag{6}$$

The baseline risk estimates allow us to estimate the baseline probability of having the event, even for individuals for which we do not have access to the outcome variable.

[ii] In the paper, we set the α mixing parameter in the elastic net estimator equal to 0.5, which leads to a neutral mixture between ℓ_1 and ℓ_2 regularization that allows for some dependence between covariates[4]. Furthermore, we use 10-fold-cross validation, which is the default value in the `glmnet` package[5] that we use. Unless stated otherwise, we use the default settings in this package.

4.1.2 Stage 2: Final Risk

In the second stage, we fit another linear logistic regression model. This time, we regress the Y_i on the treatment assignments W_i and an interaction term $W_i\widehat{p}_i$, where $\widehat{\pi}_i = \widehat{\mathbb{P}}[Y_i = 1|X_i = x_i]$ is the estimated baseline risk of individual $i = 1, \ldots, n$, which was obtained in Stage 1 via (6). Formally, the assumed model reads

$$\ln\left(\frac{\mathbb{P}[Y_i = 1|W_i = w_i, \mathsf{LP}_i = \widehat{\mathsf{LP}}_i]}{1 - \mathbb{P}[Y_i = 1|W_i = w_i, \mathsf{LP}_i = \widehat{\mathsf{LP}}_i]}\right) = \alpha_0 + \alpha_1 w_i + \alpha_2\widehat{\mathsf{LP}}_i + \alpha_3 w_i\widehat{\mathsf{LP}}_i, \tag{7}$$

for all $i = 1, \ldots, n$, where $\alpha_0, \alpha_1, \alpha_2, \alpha_3 \in \mathbb{R}$ are fixed but unknown coefficients. Risk models attempt to model possible treatment effect heterogeneity implicitly through the interaction term $W_i\widehat{\mathsf{LP}}_i$. The idea is that a statistically significant interaction between the treatment assignment status and the linear predictor–which was fitted without any information on treatment status–may indicate that the effectiveness of the treatment varies with baseline characteristics.

We fit the 2nd stage model (7) by means of non-regularized logistic regression. Akin to (6), the obtained estimates of the fitted model, $\widehat{\alpha}_j, j = 0, \ldots, 3$, can then be used to calculate the *risk prediction function* for a treatment status $w \in \{0, 1\}$:

$$\begin{aligned} \mathsf{risk}_i(w) &:= \widehat{\mathbb{P}}[Y_i = 1|W_i = w, \mathsf{LP}_i = \widehat{\mathsf{LP}}_i] \\ &= F_{\text{logistic}}\Big(\widehat{\alpha}_0 + \widehat{\alpha}_1 w + \widehat{\alpha}_2\widehat{\mathsf{LP}}_i + \widehat{\alpha}_3 w\widehat{\mathsf{LP}}_i\Big). \end{aligned} \tag{8}$$

The second stage risk model (7) gives rise to two modifications.

First, instead of explicitly fitting a baseline risk model (3) in the first stage, one may use an externally fitted baseline risk model such as the PLCO2012 model[6] to obtain estimated linear predictors $\widehat{\mathsf{LP}}_i$ for each individual. In the paper, we use the PLCO2012 linear predictors as well as linear predictors of a number of other common baseline risk models for lung cancer.

Second, one may exclude the interaction term $w\widehat{\mathsf{LP}}_i$ from the set of predictor variables in the second stage model (7) if there is no sufficient evidence of treatment effect heterogeneity. In our paper, we fit the second stage model twice: once with and once without the interaction term as regressor. The latter case is equivalent to setting $\alpha_3 = 0$ in (7). Then, we calculate the likelihood of each fitted model and perform a likelihood ratio test[7] using the two likelihoods. The likelihood ratio test tests the null hypothesis that the interaction term can be omitted from the model. If the likelihood ratio tests rejects this null hypothesis at a reasonable level (we use the 5% level in our paper), we keep the interaction term in the second stage model (7). If the test sustains the null hypothesis, we drop the interaction term and use the model that was fitted without the interaction term as the final model for the second stage.

4.2 Effect Models

Aside from risk models, *effect models* have been proposed to model treatment effect heterogeneity[2]. Effect models attempt to model possible treatment effect heterogeneity explicitly by adding interaction terms between the treatment assignment

status and baseline characteristics along which the treatment assignment is suspected to be of varying effectiveness. The idea is that when a model with such interaction terms is fitted by means of regularized logistic regression, the regularization shrinks coefficients of interaction terms and other predictor variables with low explanatory power for the outcome to zero, resulting in a parsimonious model for treatment effect heterogeneity.

As a first step in effect modeling, one must specify a set $\mathcal{I} \subset \{1, \ldots, d\}$ that contains the explanatory variables that are to be interacted with the treatment assignment variable.[iii] Then, for all individuals $i = 1, \ldots, n$, an effect model is a logistic linear regression model which reads

$$\ln\left(\frac{\mathbb{P}[Y_i = 1 \mid W_i = w_i, X_i = x_i]}{1 - \mathbb{P}[Y_i = 1 \mid W_i = w_i, X_i = x_i]}\right) = \beta_0 + x_i^\top \beta + \gamma_0 w_i + \sum_{\{j : j \in \mathcal{I}\}} \gamma_j w_i x_{ij}, \quad (9)$$

where x_{ij} is the j-th element in the d-dimensional vector x_i, while $\beta_0, \gamma_0 \in \mathbb{R}, \beta \in \mathbb{R}^d$, and $\gamma := \{\gamma_j\}_{j \in \mathcal{I}} \in \mathbb{R}^{|\mathcal{I}|}$ are fixed but unknown coefficients.

It is recommended to fit the effect model (9) by means of regularized logistic regression[2]. Like in the first stage of the risk model, we apply elastic net regularization[4] and choose the final regularization term by means of cross validation[5]. After fitting, we obtain possibly sparse estimates $(\widehat{\beta}_0, \widehat{\beta}, \widehat{\gamma}_0, \widehat{\gamma})$ of the coefficients $\beta_0, \beta, \gamma_0, \gamma)$ in effect model (9).

Given some treatment assignment status $w \in \{0, 1\}$, the *risk prediction function* of the fitted effect model is, for all individuals $i = 1, \ldots, n$, given by

$$\begin{aligned} \mathsf{risk}_i(w) &= \widehat{\mathbb{P}}[Y_i = 1 | W_i = w, X_i = x_i] \\ &= F_{\text{logistic}}\left(\widehat{\beta}_0 + x_i^\top \widehat{\beta} + \widehat{\gamma}_0 w + \sum_{\{j : j \in \mathcal{I}\}} \widehat{\gamma}_j w x_{ij}\right). \end{aligned} \quad (10)$$

The effect model (9) gives rise to one modification. It may occur that the estimate of the coefficient of the treatment assignment, γ_0, is shrunk by the regularized fitting process to be exactly zero, that is, $\widehat{\gamma}_0 = 0$, while some coefficients of the interaction terms may be nonzero. This case yields a problematic interpretation since it means that the treatment is ineffective, but heterogeneous along baseline characteristics. To avoid this scenario, one may opt to not regularize γ_0 in the fitting process, which is what we do in our paper.

4.3 Estimation of Benefits

Suppose we have fitted a predictive model, either a risk model (Section 4.1) or an effect model (Section 4.2). Consider the risk prediction function $\{0, 1\} \ni w \mapsto \mathsf{risk}_i(w), i = 1, \ldots, n$, associated with the fitted model. For the observed treatment

[iii] The choice $\mathcal{I} = \varnothing$ is also permitted (i.e. no variable is interacted), but we decidedly do not reflect this possibility in our notation for the sake of notational brevity.

status of individual i, $W_i = w_i$, we define the *regular risk* as $\mathsf{risk}_i^{\mathsf{reg}} = \mathsf{risk}_i(w_i)$. Furthermore, define the *reversed* treatment assignment by

$$W_i^{\text{rev}} = \begin{cases} 1 & \text{if } W_i = 0, \\ 0 & \text{if } W_i = 1. \end{cases}$$

Thereupon, at $W_i^{\text{rev}} = w_i^{\text{rev}}$, we define the *reverse risk* by $\mathsf{risk}_i^{\mathsf{rev}} = \mathsf{risk}_i(w_i^{\text{rev}})$. We estimate the absolute benefit in (1) by the *predicted absolute benefit*, $\widehat{\theta}_i$,

$$\widehat{\theta}_i = \mathsf{risk}_i - \mathsf{risk}_i^{\mathsf{rev}}$$

and the relative benefit in (2) by the *predicted relative benefit*, $\widehat{\theta}_i^{rel}$,

$$\widehat{\theta}_i^{\text{rel}} = \frac{\mathsf{risk}_i}{\mathsf{risk}_i^{\mathsf{rev}}} - 1.$$

We use the predictive benefits to measure the strength of treatment effect heterogeneity, which will be discussed in the next section.

5 Measuring Treatment Effect Heterogeneity

Suppose we have obtained predictive absolute and relative benefits, $\widehat{\theta}_i$ and $\widehat{\theta}_i^{\text{rel}}$, respectively, for $i = 1, \ldots, n$. Suppose furthermore that the observations $\{1, \ldots, n\}$ can be partitioned into m disjoint groups which we index by $1, \ldots, m$. Denote the group membership of observation i by $G_i \in \{1, \ldots, m\} = \mathcal{G}$. The grouping is usually done based on characteristics in the covariates X_i.

5.1 Predicted Benefits

Given a group $g \in \mathcal{G}$, denote the group-level absolute predicted benefit and relative predicted benefit by

$$\begin{aligned} \widehat{\theta}^{(g)} &= |\{i : G_i = g\}|^{-1} \sum_{\{i:G_i=g\}} \widehat{\theta}_i \quad \text{and} \\ \widehat{\theta}^{\text{rel},(g)} &= |\{i : G_i = g\}|^{-1} \sum_{\{i:G_i=g\}} \widehat{\theta}_i^{\text{rel}}, \end{aligned} \tag{11}$$

respectively. The group-level absolute predicted benefit $\widehat{\theta}^{(g)}$ estimates the within-group conditional treatment effect

$$\mathsf{CATE}^{(g)} = \mathbb{E}\big[Y_i | X_i = x_i, W_i = 1, G_i = g\big] - \mathbb{E}\big[Y_i | X_i = x_i, W_i = 0, G_i = g\big] \tag{12}$$

and the relative predictive benefit $\widehat{\theta}^{\text{rel},(g)}$ estimates the within-group relative conditional average treatment effect

$$\begin{aligned} \mathsf{RCATE}^{(g)} &= \frac{\mathbb{E}\big[Y_i | X_i = x_i, W_i = 1, G_i = g\big]}{\mathbb{E}\big[Y_i | X_i = x_i, W_i = 0, G_i = g\big]} - 1 \\ &= \frac{\mathbb{P}\big[Y_i = 1 | X_i = x_i, W_i = 1, G_i = g\big]}{\mathbb{P}\big[Y_i = 1 | X_i = x_i, W_i = 0, G_i = g\big]} - 1, \end{aligned} \tag{13}$$

for groups $g \in \mathcal{G}$ and individuals $i = 1, \ldots, n$.

5.2 Observed Benefits

In addition to predicted benefits, observed benefits are useful in prediction models. Observed benefits help in assessing the calibration of a predictive model by comparing how well the predicted benefits align with the empirically observed benefits. Just like predicted benefits, we distinguish between absolute and relative effects.

5.2.1 Observed Absolute Benefit

For each group $g \in \mathcal{G}$, calculate the group-level observed absolute benefit, $\mathsf{OAB}^{(g)}$, which is defined as

$$\begin{aligned} \mathsf{OAB}^{(g)} =& |\{i : G_i = g, W_i = 1\}|^{-1} \sum_{\{i:G_i=g,W_i=1\}} Y_i - \\ & |\{i : G_i = g, W_i = 0\}|^{-1} \sum_{\{i:G_i=g,W_i=0\}} Y_i. \end{aligned} \tag{14}$$

Since the group-level observed absolute benefit is essentially the difference in the average group-level occurrence of the event, we can view it as an estimate of within-group conditional treatment effect.

Due to its “difference in means” structure, we can use elementary statistical theory to construct confidence intervals for the absolute observed benefit $\mathsf{OAB}^{(g)}$. Concretely, for a given significance level $\alpha \in (0, 0.5)$, we use the $(1-\alpha)$-confidence interval implied by a two-sample t-test, where we use the first set of summands in (14) as the first sample, and the second set of summands as the second sample. We assume non-equal population variances of the two samples, which renders the test a Welch t-test.

5.2.2 Observed Relative Benefit

For each group $g \in \mathcal{G}$, calculate the group-level observed relative benefit, $\mathsf{ORB}^{(g)}$, by the relative risk

$$\mathsf{ORB}^{(g)} = \frac{|\{i : G_i = g, W_i = 1\}|^{-1} \sum_{\{i:G_i=g,W_i=1\}} Y_i}{|\{i : G_i = g, W_i = 0\}|^{-1} \sum_{\{i:G_i=g,W_i=0\}} Y_i} - 1. \tag{15}$$

Observe that the relative observed benefit corresponds to the ratio of the two summands that constitute the absolute observed benefit in equation (14) and can be viewed as a ratio of two proportions. The observed relative benefit estimates the group-level relative conditional average treatment effect, RCATE, in (13).

We describe in the following how to constructing confidence intervals for the group-level observed relative benefit $\mathsf{ORB}^{(g)}$.

We first need some ancillary quantities. Within a given group $g \in \mathcal{G}$, let the

constants

$$\begin{aligned}
N_g^{(Y=1,W=1)} &= |\{i : G_i = g, Y_i = 1, W_i = 1\}|,\\
N_g^{(Y=0,W=1)} &= |\{i : G_i = g, Y_i = 0, W_i = 1\}|,\\
N_g^{(Y=1,W=0)} &= |\{i : G_i = g, Y_i = 1, W_i = 0\}|, \text{ and}\\
N_g^{(Y=0,W=0)} &= |\{i : G_i = g, Y_i = 0, W_i = 0\}|
\end{aligned} \tag{16}$$

count the number of treated events, treated non-events, untreated events, and untreated non-events, respectively. Similarly, let

$$N_g^{(W=1)} = |\{i : G_i = g, W_i = 1\}| \quad \text{and} \quad N_g^{(W=0)} = |\{i : G_i = g, W_i = 0\}| \tag{17}$$

count the number of treated and untreated samples, respectively, in group g. For a given group $g \in \mathcal{G}$, denote by

$$Z_g = \frac{N_g^{(Y=0,W=1)}}{N_g^{(Y=1,W=1)}} \Big/ N_g^{(W=1)} + \frac{N_g^{(Y=0,W=0)}}{N_g^{(Y=1,W=0)}} \Big/ N_g^{(W=0)} \tag{18}$$

the sum of the relative non-event occurrence for the treatment and control group (normalized by the number of samples in each of these groups).

Finally, for a given group $g \in \mathcal{G}$, a corresponding observed relative benefit $\mathsf{ORB}^{(g)}$, a significance level $\alpha \in (0, 0.5)$, and a left $(1-\alpha)$-quantile of the standard normal distribution $z_{1-\alpha/2}$, denote the real-valued interval $\mathcal{C}^{(g)}_{\log(\mathsf{RCATE})}$ by

$$\mathcal{C}^{(g)}_{\log(\mathsf{RCATE})} = \Big[\log\big(\mathsf{ORB}^{(g)}\big) - z_{1-\alpha/2}\sqrt{Z_g},\ \log\big(\mathsf{ORB}^{(g)}\big) + z_{1-\alpha/2}\sqrt{Z_g}\Big]. \tag{19}$$

The interval $\mathcal{C}^{(g)}_{\log(\mathsf{RCATE})}$ is a $(1-\alpha)$-confidence interval for $\log(\mathsf{RCATE}^{(g)})$. A simple exponential transformation yields the desired $(1-\alpha)$-confidence interval for $\mathsf{RCATE}^{(g)}$, denoted by $\mathcal{C}^{(g)}_{\mathsf{RCATE}}$ and defined as

$$\begin{aligned}
\mathcal{C}^{(g)}_{\mathsf{RCATE}} &= \left[\exp\Big(\log\big(\mathsf{ORB}^{(g)}\big) - z_{1-\alpha/2}\sqrt{Z_g}\Big), \exp\Big(\log\big(\mathsf{ORB}^{(g)}\big) + z_{1-\alpha/2}\sqrt{Z_g}\Big)\right]\\
&= \left[\mathsf{ORB}^{(g)} \exp\Big(-z_{1-\alpha/2}\sqrt{Z_g}\Big),\ \mathsf{ORB}^{(g)} \exp\Big(z_{1-\alpha/2}\sqrt{Z_g}\Big)\right].
\end{aligned} \tag{20}$$

6 Causal Random Forests

Causal (random) forests[1] yield estimates $\widehat{\theta}_i$ of the CATE for each individual i with observed covariate vector x_i. That is, it estimates

$$\mathsf{CATE}(x_i) = \mathbb{E}[Y_i(1) - Y_i(0) \mid X_i = x] = \mathbb{P}[Y_i(1) = 1 \mid X_i = x_i] - \mathbb{P}[Y_i(0) = 1 \mid X_i = x_i],$$

see (1). This estimate is, under regularity conditions, consistent and asymptotically normally distributed[1]. However, when the outcome variable Y_i is binary, which is

the case in our investigation, then neither $\mathbb{P}[Y_i(1) = 1 \mid X_i = x_i]$ nor $\mathbb{P}[Y_i(0) = 1 \mid X_i = x_i]$ can be estimated by causal forests, only their difference. Hence, unlike in risk or effect models, no estimate of relative CATE can be directly obtained. Hence, to ensure that relative CATE estimates of risk and effect models can be compared with causal forest CATE estimates, we need to transform the latter in a meaningful and intuitive way.

In the following, we motivate a transformation to transform causal forest CATE estimates to a relative quantity for a given group $g \in \mathcal{G}$ whose group-level predicted benefit we are interested in. First, calculate the the causal forest's group-level absolute predicted benefit by $\widehat{\theta}^{(g)}$ according to equation 8 in Athey & Wager (2019)[8], which is the ordinary way in which group-level absolute effects are calculated in the `grf` package[1].

Recall the definition of the group-level CATE in (12) for a group $g \in \mathcal{G}$ is defined as

$$\theta^{(g)} = \mathbb{E}\big[Y_i|X_i = x_i, W_i = 1, G_i = g\big] - \mathbb{E}\big[Y_i|X_i = x_i, W_i = 0, G_i = g\big],$$

which can be rewritten as

$$\mathbb{E}\big[Y_i|X_i = x_i, W_i = 1, G_i = g\big] = \theta^{(g)} + \mathbb{E}\big[Y_i|X_i = x_i, W_i = 0, G_i = g\big].$$

Subsequently, we can express the relative CATE in (13) as

$$\begin{aligned}\theta^{\text{rel},(g)} &= \frac{\mathbb{E}\big[Y_i|X_i = x_i, W_i = 1, G_i = g\big]}{\mathbb{E}\big[Y_i|X_i = x_i, W_i = 0, G_i = g\big]} - 1 \\ &= \frac{\theta^{(g)} + \mathbb{E}\big[Y_i|X_i = x_i, W_i = 0, G_i = g\big]}{\mathbb{E}\big[Y_i|X_i = x_i, W_i = 0, G_i = g\big]} - 1\end{aligned} \tag{21}$$

Since we can consistently estimate the average effect $\theta^{(g)}$ via $\widehat{\theta}^{(g)}$, we need a consistent estimate of the controls' average group-level mortality $\mathbb{E}\big[Y_i|X_i = x_i, W_i = 0, G_i = g\big]$ to consistently estimate the relative effect $\theta^{\text{rel},(g)}$. Since we use data from randomized trials, we can estimate the average mortality in the control group for group g via the observed lung cancer mortality rate

$$\mathsf{LCMR}^{(g)}_{\text{control}} = |\{i : G_i = g, W_i = 0\}|^{-1} \sum_{\{i:G_i=g, W_i=0\}} Y_i.$$

We can now construct an estimator $\theta^{\text{rel},(g)}$ of the relative effect $\theta^{\text{rel},(g)}$ by replacing all quantities in (21) with their respective estimator, that is, $\theta^{(g)}$ by $\widehat{\theta}^{(g)}$ and $\mathbb{E}\big[Y_i|X_i = x_i, W_i = 0, G_i = g\big]$ by $\mathsf{LCMR}^{(g)}_{\text{control}}$. This is exactly the transformation given in the main paper.

To obtain a standard error of the relative estimator $\theta^{\text{rel},(g)}$ constructed with the transformation described above, we use the Delta Method[7] based on the standard error of RCATE $\widehat{\theta}^{(g)}$ computed by equation 8 in Athey & Wager (2019)[8].

Appendix

A (Regularized) Logistic Regression

Assume we are interested in predicting binary outcomes Y_i via q-dimensional random vectors Z_i, where $\{(Z_i, Y_i)\}_{i=1}^n$ is a random sample. Hence, since $Y_i|Z_i \sim \mathsf{Bernoulli}(p_{i,Z})$, we are effectively interested in estimating the parameter $p_{i,Z} = \mathbb{P}[Y_i = 1 \mid Z_i]$, where the subscript "$Z$" reminds us that $p_{i,Z}$ is a conditional probability.

We assume a logistic linear model, which assumes the linear identity

$$\ln\left(\frac{p_{i,Z}}{1-p_{i,Z}}\right) = \beta_0 + Z_i^\top \beta, \tag{22}$$

for all $i = 1, \dots, n$, where $(\beta_0, \beta) = (\beta_0, \beta_1, \dots, \beta_q) \in \mathbb{R}^{q+1}$ is some fixed vector of coefficients. Rearranging identifies the probability of interest, $p_{i,Z}$, as

$$p_{i,Z} = \left(1 + \exp\left(-\beta_0 - Z_i^\top \beta\right)\right)^{-1}.$$

We can fit the model (22) by *logistic regression*, which performs maximum likelihood estimation over the coefficients. The corresponding optimization problem can be shown to solve

$$(\widehat{\beta}_0, \widehat{\beta}) \in \arg \min_{(\beta_0,\beta)\in\mathbb{R}^{p+1}} \left\{ -\frac{1}{n}\sum_{i=1}^n \left(Y_i(\beta_0 + Z_i^\top\beta) - \ln\left(1 + \mathrm{e}^{\beta_0 + Z_i^\top \beta}\right)\right)\right\}. \tag{23}$$

However, when there are more explanatory variables than observations, $q \geq n$, then the solution $(\widehat{\beta}_0, \widehat{\beta})$ may be degenerate. In this case, adding a regularization penalty to the objective function in (23) enforces sparsity in the solution and thereby renders the solution stable again. Concretely, given fixed $\lambda_n \geq 0$ and $\alpha \in [0, 1]$, *regularized logistic regression* solves

$$\begin{aligned}(\widehat{\beta}_0, \widehat{\beta}) \in \arg \min_{(\beta_0,\beta)\in\mathbb{R}^{p+1}} \Bigg\{ & -\frac{1}{n}\sum_{i=1}^n \left(Y_i(\beta_0 + Z_i^\top\beta) - \ln\left(1 + \mathrm{e}^{\beta_0 + Z_i^\top \beta}\right)\right) + \\ & + \lambda_n\left((1-\alpha)\|\beta\|_2^2 + \alpha\|\beta\|_1\right)\Bigg\}.\end{aligned} \tag{24}$$

Observe that for $\alpha = 0.5$, we obtain the elastic net penalty and for $\alpha = 1$, we obtain the Lasso penalty. We can find an appropriate choice of λ_n by cross-validation over a grid of candidate values for λ_n[5].

Suppose we have obtained an estimate $(\widehat{\beta}_0, \widehat{\beta})$ of (β_0, β) in the identity (22), either by regularized or non-regularized logistic regression. Then we can estimate the probabilities of interest $\mathbb{P}[Y_i = 1 \mid X_i]$ by

$$\widehat{p}_{i,Z} = \left(1 + \exp\left(-\widehat{\beta}_0 - Z_i^\top \widehat{\beta}\right)\right)^{-1}.$$

Supplementary Tables

Table S1: Designs of NELSON and NLST

	NELSON	NLST
**Intervention group**	Computed tomography screening	Computed tomography screening
**Control group**	No screening	Chest radiography screening
**Eligibility criteria**<br>**Age**<br>**Smoking criteria**	 50-74<br>Having smoked at least 15 cigarettes per day for ≥25 years or 10 cigarettes per day for ≥30 years, currently smoking or quitting <10 years ago	 55-74<br>Having smoked at least 30 pack-years, currently smoking or quitting <15 years ago
**Exclusion criteria**	Moderate or bad self-reported health and unable to climb two flights of<br>stairs; bodyweight >= 140 kilograms; Current or past renal cancer, melanoma or breast cancer; Lung cancer diagnosed < 5 years ago or >5 years but still receiving treatment; chest CT examination <1 year ago	Previous diagnosis of lung cancer; chest radiography examination < 1.5 years before enrollment; hemoptysis; unexplained weight loss of > 6.8 kilograms in the preceding year
**Enrollment period**	December 2003 through July 2006	August 2002 through April 2004
**Number of centres**	Four (three Dutch centres, 1 Belgian; only the Dutch centres were included in this study)	33 centres in the United States
**Screening period**	January 2004 through December 2012	August 2002 through September 2007
**Follow-up duration**	10 years or December 31st 2015; whichever came first	7 years or December 31st 2009; whichever came first
**Screening schedule**	Four CT screenings with different intervals: at baseline, year 1 (1-year interval), year 3 (2-year interval) and year 5.5 (2.5-year interval)	Three CT screenings with a one-year interval: at baseline, year 1 and year 2. A similar screening schedule was applied to the control group.

Table S2: Characteristics of methods for evaluating heterogeneity in screening effectiveness

Method	Type	Description	Blinded for intervention	Advantages	Limitations
**One-variable-at-a-time**	Traditional sub-group analyses	Compares the rate-ratio for lung cancer mortality between the control and intervention arm across individual characteristics. The rate-ratios are derived as the ratios of event rates, under the assumption of a Poisson distribution for the number of events.	Not applicable	Straightforward interpretation	Does not account for other covariates<br><br>Low statistical power<br><br>High potential for multiplicity
**Risk-prediction models**	Traditional sub-group analyses	Compares the rate-ratio for lung cancer mortality between the control and intervention arm across quintiles of baseline risk without explicitly modeling screening effectiveness.	Not applicable	Straightforward interpretation<br><br>Accounts for covariates included in the original models	Does not directly incorporate screening effectiveness<br><br>Does not account for risk-factors not included in the original models
**Risk modelling**	Predictive-modelling	Estimates the risk for lung cancer mortality blinded for the intervention.	Yes	Predictions for benefit generally well-calibrated<br><br>Accounts for covariates	Does not allow inclusion of treatment-by-covariate interaction terms
**Effect-modelling**	Predictive-modelling	Estimates the risk for lung cancer mortality unblinded for the intervention	No	Improved discrimination for benefit in the presence of true interaction effects<br><br>Accounts for covariates	Prone to overfitting (even in the presence of true interaction effects)
**Causal forests**	Machine-learning	Tree-based ensemble learning method that does not make distributional assumptions regarding the data	No	Ability to implicitly estimate heterogeneous treatment effects without explicitly modeling heterogeneity through interaction terms<br><br>Accounts for covariates<br><br>No linearity assumptions required	Interpretation is not straightforward: Only provides graphical representation of heterogeneity in screening effectiveness<br><br>Unable to provide C-Statistics for LCM<br><br>Estimates cannot be externally validated

Table S3: Parameter estimates for the first stage of the risk-modelling approach for overall LCM

Risk-factors	NELSON parameter estimates	NLST parameter estimates
**Intercept**	-8.35	-8.93
**Age (per 1-year increase)**	0.06	0.06
**Female sex* (binary)**	-0.16	-0.14
**Personal history of cancer (binary)**	0.06	0.31
**Body-mass index, per 1 unit increase**	-0.04	-0.03
**Level of education, per 1 unit increase**	-0.11	-0.10
**Current smoking status** (binary)**	0.51	0.42
**Number of cigarettes per day, per 1 unit increase**	0.02	0.02
**Number of years smoked, per 1 unit increase**	0.05	0.04
**Number of years since smoking cessation, per 1 unit increase**	-0.03	-0.02
**Chronic obstructive pulmonary disease (binary)**	-	0.36
**Presence of emphysema (binary)**	-	0.37
**Family history of lung cancer (binary)**	Not inquired in NELSON	0.16
**Asian race/ethnic group*** (binary)**	Not inquired in NELSON	-0.21
**Black race/ethnic group*** (binary)**	Not inquired in NELSON	0.12
**Hispanic race/ethnic group*** (binary)**	Not inquired in NELSON	-1.08
**American Indian or Alaskan Native race/ethnic group*** (binary)**	Not inquired in NELSON	0.32

Table notes:*compared to male sex, **compared to former smokers, ***compared to a White race/ethnic group

Parameter coefficients were shrunk through penalized (elastic net) logistic regression

Table S4: Parameter estimates for the effect-model approaches for overall LCM

Risk-factors	NELSON parameter estimates	NLST parameter estimates
**Intercept**	-8.24	-8.83
**Age (per 1-year increase)**	0.06	0.05
**Female sex* (binary)**	-0.17	-0.13
**Personal history of cancer (binary)**	0.08	0.30
**Body-mass index, per 1 unit increase**	-0.04	-0.03
**Level of education, per 1 unit increase**	-0.11	-0.10
**Current smoking status** (binary)**	0.52	0.42
**Number of cigarettes per day, per 1 unit increase**	0.02	0.02
**Number of years smoked, per 1 unit increase**	0.05	0.04
**Number of years since smoking cessation, per 1 unit increase**	-0.03	-0.02
**Chronic obstructive pulmonary disease (binary)**	-	0.36
**Presence of emphysema (binary)**	-	0.37
**Family history of lung cancer (binary)**	Not inquired in NELSON	0.15
**Asian race/ethnic group*** (binary)**	Not inquired in NELSON	-0.19
**Black race/ethnic group*** (binary)**	Not inquired in NELSON	0.11
**Hispanic race/ethnic group*** (binary)**	Not inquired in NELSON	-1.03
**American Indian or Alaskan Native race/ethnic group*** (binary)**	Not inquired in NELSON	0.30
**Native Hawaiian or Pacific Islander race/ethnic group*** (binary)**	Not inquired in NELSON	0.04
**Screening effectiveness (binary)**	-0.33	-0.17

Table notes:*compared to male sex, **compared to former smokers, ***compared to a White race/ethnic group

Parameter coefficients were shrunk through penalized (elastic net) logistic regression

Table S5: Parameter estimates for the second stage risk-models for overall LCM

	First-stage model based on individual risk factors	First-stage model based on the LLPv3 model	First-stage model based on the PLCOm2012 model
**Estimates based on NELSON**			
**Intercept (standard error)**	-4.19 (0.10)	-3.92 (0.09)	-4.04 (0.09)
**Screening effectiveness (binary) (standard error)**	-0.32 (0.10)	-0.13 (0.14)	-0.32 (0.10)
**Linear predictor of the first stage model**	23.28 (1.78)	36.90 (3.97)	23.11 (1.92)
**Interaction between the screening effectiveness parameter and the linear predictor of the first stage model (only included in the model when statistically significant through a likelihood ratio test) (standard error)**	-	-12.46 (6.11)	-
**Estimates based on NLST**			
**Intercept (standard error)**	-4.49 (0.06)	-4.39 (0.06)	-4.44 (0.06)
**Screening effectiveness (binary) (standard error)**	-0.18 (0.07)	-0.16 (0.07)	-0.17 (0.07)
**Linear predictor of the first stage model (standard error)**	25.96 (1.17)	22.96 (1.28)	12.36 (0.63)
**Interaction between the screening effectiveness parameter and the linear predictor of the first stage model (only included in the model when statistically significant through a likelihood ratio test) (standard error)**	-	-	-

Table S6: Parameter estimates for the effect-model approaches for overall LCM (with interactions between screening effectiveness and age-group)

Risk-factors	NELSON parameter estimates	NLST parameter estimates
**Intercept**	-7.99	-8.73
**Age (per 1-year increase)**	0.05	0.05
**Female sex* (binary)**	-0.07	-0.13
**Personal history of cancer (binary)**	-	0.29
**Body-mass index, per 1 unit increase**	-0.03	-0.03
**Level of education, per 1 unit increase**	-0.09	-0.09
**Current smoking status** (binary)**	0.47	0.41
**Number of cigarettes per day, per 1 unit increase**	0.02	0.02
**Number of years smoked, per 1 unit increase**	0.05	0.04
**Number of years since smoking cessation, per 1 unit increase**	-0.02	-0.01
**Chronic obstructive pulmonary disease (binary)**	-	0.35
**Presence of emphysema (binary)**	-	0.37
**Family history of lung cancer (binary)**	Not inquired in NELSON	0.15
**Asian race/ethnic group*** (binary)**	Not inquired in NELSON	-0.17
**Black race/ethnic group*** (binary)**	Not inquired in NELSON	0.10
**Hispanic race/ethnic group*** (binary)**	Not inquired in NELSON	-0.97
**American Indian or Alaskan Native race/ethnic group*** (binary)**	Not inquired in NELSON	0.27
**Native Hawaiian or Pacific Islander race/ethnic group*** (binary)**	Not inquired in NELSON	-
**Screening effectiveness (binary)**	-0.32	-0.18
**Screening effectiveness interaction with age-group 50-54 (binary)**	-	Age-group not included in NLST
**Screening effectiveness interaction with age-group 55-59 (binary)**	-0.04	-
**Screening effectiveness interaction with age-group 60-64 (binary)**	0.01	-
**Screening effectiveness interaction with age-group 65-69 (binary)**	-	0.04
**Screening effectiveness interaction with age-group 70-74 (binary)**	0.01	0.03

Table notes:*compared to male sex, **compared to former smokers, ***compared to a White race/ethnic group

Parameter coefficients were shrunk through penalized (elastic net) logistic regression

Table S7: Parameter estimates for the effect-model approaches for overall LCM (with interactions between screening effectiveness and smoking status and years since smoking cessation)

Risk-factors	NELSON parameter estimates	NLST parameter estimates
**Intercept**	-8.19	-8.81
**Age (per 1-year increase)**	0.06	0.05
**Female sex* (binary)**	-0.17	-0.12
**Personal history of cancer (binary)**	0.07	0.29
**Body-mass index, per 1 unit increase**	-0.04	-0.03
**Level of education, per 1 unit increase**	-0.11	-0.09
**Current smoking status** (binary)**	0.44	0.43
**Number of cigarettes per day, per 1 unit increase**	0.02	0.02
**Number of years smoked, per 1 unit increase**	0.05	0.04
**Number of years since smoking cessation, per 1 unit increase**	-0.03	-0.01
**Chronic obstructive pulmonary disease (binary)**	-	0.35
**Presence of emphysema (binary)**	-	0.37
**Family history of lung cancer (binary)**	Not inquired in NELSON	0.15
**Asian race/ethnic group*** (binary)**	Not inquired in NELSON	-0.16
**Black race/ethnic group*** (binary)**	Not inquired in NELSON	0.09
**Hispanic race/ethnic group*** (binary)**	Not inquired in NELSON	-0.95
**American Indian or Alaskan Native race/ethnic group*** (binary)**	Not inquired in NELSON	0.26
**Native Hawaiian or Pacific Islander race/ethnic group*** (binary)**	Not inquired in NELSON	-
**Screening effectiveness (binary)**	-0.40	-0.15
**Screening effectiveness interaction with current smoking status (binary)**	0.13 ¶	-
**Screening effectiveness interaction with smoking cessation <5 years ago (binary)**	-0.06	-
**Screening effectiveness interaction with smoking cessation 5-10 years ago (binary)**	-0.01	-
**Screening effectiveness interaction with smoking cessation ≥10 years ago (binary)**	Not included in trial	-0.17

Table notes:*compared to male sex, **compared to former smokers, ***compared to a White race/ethnic group

¶ relative excess risk due to interaction (RERI): -0.50 (95% confidence interval: -1.89, 0.89 ; p-value = 0.76) Tests were two-sided based on the Delta method through R-package "InteractionR" (version 0.1.7).[9,10]

Parameter coefficients were shrunk through penalized (elastic net) logistic regression

Table S8: Parameter estimates for the effect-model approaches for overall LCM (with interactions between screening effectiveness and accumulated pack-years)

Risk-factors	NELSON parameter estimates	NLST parameter estimates
**Intercept**	-7.85	-8.68
**Age (per 1-year increase)**	0.06	0.06
**Female sex* (binary)**	-0.18	-0.13
**Personal history of cancer (binary)**	0.09	0.30
**Body-mass index, per 1 unit increase**	-0.04	-0.03
**Level of education, per 1 unit increase**	-0.11	-0.10
**Current smoking status** (binary)**	0.52	0.41
**Number of cigarettes per day, per 1 unit increase**	0.02	0.02
**Number of years smoked, per 1 unit increase**	0.04	0.04
**Number of years since smoking cessation, per 1 unit increase**	-0.03	-0.02
**Chronic obstructive pulmonary disease (binary)**	-	0.35
**Presence of emphysema (binary)**	-	0.37
**Family history of lung cancer (binary)**	-	0.15
**Asian race/ethnic group*** (binary)**	Not inquired in NELSON	-0.18
**Black race/ethnic group*** (binary)**	Not inquired in NELSON	0.12
**Hispanic race/ethnic group*** (binary)**	Not inquired in NELSON	-1.01
**American Indian or Alaskan Native race/ethnic group*** (binary)**	Not inquired in NELSON	0.30
**Native Hawaiian or Pacific Islander race/ethnic group*** (binary)**	Not inquired in NELSON	0.04
**Screening effectiveness (binary)**	-0.15	-0.12
**Screening effectiveness interaction with <30 accumulated pack-years (binary)**	-0.60	Participants with <30 pack-years were not included in NLST
**Screening effectiveness interaction with 30-39 accumulated pack-years (binary)**	-0.23	-0.39
**Screening effectiveness interaction with 40-49 accumulated pack-years (binary)**	-0.17	-
**Screening effectiveness interaction with ≥50 accumulated pack-years (binary)**	0.06	-

Table notes:*compared to male sex, **compared to former smokers, ***compared to a White race/ethnic group

Parameter coefficients were shrunk through penalized (elastic net) logistic regression

Table S9: Parameter estimates for the effect-model approaches for overall LCM (with interactions between screening effectiveness and sex)

Risk-factors	NELSON parameter estimates	NLST parameter estimates
**Intercept**	-8.22	-8.86
**Age (per 1-year increase)**	0.06	0.05
**Female sex* (binary)**	-0.16	-0.06
**Personal history of cancer (binary)**	0.07	0.30
**Body-mass index, per 1 unit increase**	-0.04	-0.03
**Level of education, per 1 unit increase**	-0.11	-0.10
**Current smoking status** (binary)**	0.51	0.42
**Number of cigarettes per day, per 1 unit increase**	0.02	0.02
**Number of years smoked, per 1 unit increase**	0.05	0.04
**Number of years since smoking cessation, per 1 unit increase**	-0.03	-0.02
**Chronic obstructive pulmonary disease (binary)**	NA	0.36
**Presence of emphysema (binary)**	-	0.37
**Family history of lung cancer (binary)**	-	0.15
**Asian race/ethnic group*** (binary)**	Not inquired in NELSON	-0.19
**Black race/ethnic group*** (binary)**	Not inquired in NELSON	0.11
**Hispanic race/ethnic group*** (binary)**	Not inquired in NELSON	-1.03
**American Indian or Alaskan Native race/ethnic group*** (binary)**	Not inquired in NELSON	0.31
**Native Hawaiian or Pacific Islander race/ethnic group*** (binary)**	Not inquired in NELSON	0.04
**Screening effectiveness (binary)**	-0.33	-0.11
**Screening effectiveness interaction with female sex (binary)**	-0.01¶	-0.16#

Table notes:*compared to male sex, **compared to former smokers, ***compared to a White race/ethnic group

¶ relative excess risk due to interaction (RERI): 0.96 (95% confidence interval: 0.53, 1.74 ; p-value = 0.90). Tests were two-sided based on the Delta method through R-package "InteractionR" (version 0.1.7)..[9,10]

# relative excess risk due to interaction (RERI): 0.04 (95% confidence interval: -0.68, 0.774 ; p-value = 0.46). Tests were two-sided based on the Delta method through R-package "InteractionR" (version 0.1.7)..[9,10]

Parameter coefficients were shrunk through penalized (elastic net) logistic regression

Table S10: Parameter estimates for the first stage of the risk-modelling approach in NELSON by histology

Parameter	Adenocarcinoma	Squamous cell carcinoma	Other lung cancers	Small cell carcinoma
**Intercept**	-8.34	-9.79	-9.37	-8.99
**Age (per 1-year increase)**	0.05	0.06	0.04	0.03
**Female sex (binary)***	-0.02	-0.83	-	0.0001
**Personal history of cancer (binary)**	-	0.58	-	-0.19
**Body-mass index, per 1 unit increase**	-0.05	-0.04	-0.001	0.01
**Level of education, per 1 unit increase**	-0.13	-0.06	-	-0.13
**Current smoking status (binary)****	0.18	0.76	-	0.95
**Number of cigarettes per day, per 1 unit increase**	0.01	0.03	0.002	0.02
**Number of years smoked, per 1 unit increase**	0.06	0.04	0.03	0.03
**Number of years since smoking cessation, per 1 unit increase**	-0.03	-	-	-0.04

Table notes:*compared to male sex, **compared to former smokers.

Parameter coefficients were shrunk through penalized (elastic net) logistic regression

Table S11: Parameter estimates for the first stage of the risk-modelling approach in NLST by histology

Parameter	Adenocarcinoma	Squamous cell carcinoma	Other lung cancers	Small cell carcinoma
Intercept	-9.18	-8.51	-11.09	-11.13
Age (per 1-year increase)	0.05	0.04	0.06	0.05
Female sex* (binary)	-0.16	-0.51	0.02	0.001
Personal history of cancer (binary)	0.68	-0.17	0.37	-0.08
Body-mass index, per 1 unit increase	-0.03	-0.04	-0.02	-
Level of education, per 1 unit increase	-0.04	-0.16	-0.07	-0.11
Current smoking status** (binary)	0.37	0.26	0.23	0.48
Number of cigarettes per day, per 1 unit increase	0.02	0.02	0.01	0.02
Number of years smoked, per 1 unit increase	0.04	0.04	0.05	0.05
Number of years since smoking cessation, per 1 unit increase	0.01	-0.04	-0.03	-0.04
Chronic obstructive pulmonary disease (binary)	0.17	0.43	0.39	0.37
Presence of emphysema (binary)	0.36	0.45	0.47	0.07
Family history of lung cancer (binary)	0.38	-	-	-
Asian race/ethnic group*** (binary)	-0.57	0.001	-	-0.30
Black race/ethnic group*** (binary)	0.08	-	0.40	-0.03
Hispanic race/ethnic group*** (binary)	-0.65	-0.49	-1.24	-0.65
American Indian or Alaskan Native race/ethnic group*** (binary)	0.73	-	0.10	-0.63

Table notes:*compared to male sex, **compared to former smokers, ***compared to a White race/ethnic group. Parameter coefficients were shrunk through penalized (elastic net) logistic regression

Table S12: Parameter estimates for the effect-model approaches in NELSON by histology

Parameter	Adenocarcinoma	Squamous cell carcinoma	Other lung cancers	Small cell carcinoma
**Intercept**	-8.29	-9.57	-8.72	-8.69
**Age (per 1-year increase)**	0.05	0.06	0.04	0.03
**Female sex (binary)***	-0.04	-0.86	-	-
**Personal history of cancer (binary)**	-	0.61	-	-0.14
**Body-mass index, per 1 unit increase**	-0.06	-0.04	-	0.01
**Level of education, per 1 unit increase**	-0.14	-0.06	-	-0.12
**Current smoking status (binary)****	0.19	0.78	-	0.92
**Number of cigarettes per day, per 1 unit increase**	0.01	0.03	-	0.02
**Number of years smoked, per 1 unit increase**	0.06	0.04	0.02	0.03
**Number of years since smoking cessation, per 1 unit increase**	-0.03	-	-	-0.03
**Screening effectiveness (binary)**	-0.20	-0.74	-0.28	-0.11

Table notes:*compared to male sex, **compared to former smokers

Parameter coefficients were shrunk through penalized (elastic net) logistic regression

Table S13: Parameter estimates for the effect-model approaches in NLST by histology

Parameter	Adenocarcinoma	Squamous cell carcinoma	Other lung cancers	Small cell carcinoma
**Intercept**	-9.02	-8.61	-11.07	-11.06
**Age (per 1-year increase)**	0.05	0.04	0.06	0.05
**Female sex* (binary)**	-0.14	-0.49	0.03	-
**Personal history of cancer (binary)**	0.65	-0.14	0.38	-0.08
**Body-mass index, per 1 unit increase**	-0.03	-0.04	-0.02	-
**Level of education, per 1 unit increase**	-0.04	-0.15	-0.07	-0.11
**Current smoking status** (binary)**	0.30	0.26	0.24	0.48
**Number of cigarettes per day, per 1 unit increase**	0.01	0.02	0.01	0.02
**Number of years smoked, per 1 unit increase**	0.03	0.04	0.05	0.05
**Number of years since smoking cessation, per 1 unit increase**	0.002	-0.04	-0.03	-0.04
**Chronic obstructive pulmonary disease (binary)**	0.15	0.41	0.41	0.37
**Presence of emphysema (binary)**	0.35	0.45	0.49	0.07
**Family history of lung cancer (binary)**	0.36	-	0.004	-
**Asian race/ethnic group*** (binary)**	-0.46	-	0.002	-0.29
**Black race/ethnic group*** (binary)**	0.04	-	0.44	-0.03
**Hispanic race/ethnic group*** (binary)**	-0.52	-0.45	-1.55	-0.63
**American Indian or Alaskan Native race/ethnic group*** (binary)**	0.66	-	0.25	-0.59
**Native Hawaiian or Pacific Islander race/ethnic group*** (binary)**	0.68	-	-0.82	-0.56
**Screening effectiveness (binary)**	-0.26	0.24	-0.44	-0.12

Table notes:*compared to male sex, **compared to former smokers, ***compared to a White race/ethnic group. Parameter coefficients were shrunk through penalized (elastic net) logistic regression

Table S14: Parameter estimates for the effect-model approaches in NELSON for histology-specific mortality (with interactions between screening effectiveness and smoking status and years since smoking cessation)

Parameter	Adenocarcinoma	Squamous cell carcinoma	Other lung cancers	Small cell carcinoma
**Intercept**	-8.28	-9.54	-8.72	-8.40
**Age (per 1-year increase)**	0.05	0.05	0.04	0.02
**Female sex (binary)***	-0.05	-0.84	-	-
**Personal history of cancer (binary)**	-	0.60	-	-0.09
**Body-mass index, per 1 unit increase**	-0.06	-0.04	-	0.00
**Level of education, per 1 unit increase**	-0.14	-0.06	-	-0.12
**Current smoking status (binary)****	0.08	0.86	-	0.88
**Number of cigarettes per day, per 1 unit increase**	0.02	0.03	0.02	0.02
**Number of years smoked, per 1 unit increase**	0.06	0.04	-	0.03
**Number of years since smoking cessation, per 1 unit increase**	-0.01	-	-	-0.03
**Screening effectiveness (binary)**	-0.38	-0.80	-0.28	-0.11
**Screening effectiveness interaction with current smoking status (binary)**	0.33 ¶	-0.03#	-	-
**Screening effectiveness interaction with smoking cessation <5 years ago (binary)**	-	-	-	-
**Screening effectiveness interaction with smoking cessation 5-10 years ago (binary)**	-0.40	0.61	-	-
**Screening effectiveness interaction with smoking cessation ≥10 years ago (binary)**	Participants with ≥10 years since cessation were not included in NELSON	Participants with ≥10 years since cessation were not included in NELSON	Participants with ≥10 years since cessation were not included in NELSON	Participants with ≥10 years since cessation were not included in NELSON

Table notes:*compared to male sex, **compared to former smokers. Parameter coefficients were shrunk through penalized (elastic net) logistic regression

¶ relative excess risk due to interaction (RERI): 0.78, interaction contrast (95% confidence interval: -0.80, 2.36; p-value =0.17). Tests were two-sided based on the Delta method through R-package “InteractionR” (version 0.1.7)..[9,10]

# relative excess risk due to interaction (RERI): 0.32, interaction contrast (95% confidence interval: -6.18, 6.83.; p-value =0.46). Tests were two-sided based on the Delta method through R-package “InteractionR” (version 0.1.7).[9,10]

Table S15: Parameter estimates for the effect-model approaches in NLST for histology-specific mortality (with interactions between screening effectiveness and smoking status and years since smoking cessation)

Parameter	Adenocarcinoma	Squamous cell carcinoma	Other lung cancers	Small cell carcinoma
**Intercept**	-9.15	-8.63	-10.90	-10.89
**Age (per 1-year increase)**	0.05	0.04	0.06	0.05
**Female sex* (binary)**	-0.16	-0.50	0.01	-
**Personal history of cancer (binary)**	0.68	-0.15	0.36	-0.03
**Body-mass index, per 1 unit increase**	-0.03	-0.04	-0.02	-
**Level of education, per 1 unit increase**	-0.05	-0.16	-0.07	-0.10
**Current smoking status** (binary)**	0.41	0.30	0.22	0.46
**Number of cigarettes per day, per 1 unit increase**	0.02	0.02	0.01	0.02
**Number of years smoked, per 1 unit increase**	0.04	0.04	0.05	0.05
**Number of years since smoking cessation, per 1 unit increase**	0.03	-0.05	-0.03	-0.04
**Chronic obstructive pulmonary disease (binary)**	0.17	0.42	0.39	0.35
**Presence of emphysema (binary)**	0.36	0.45	0.48	0.06
**Family history of lung cancer (binary)**	0.38	-	-	-
**Asian race/ethnic group*** (binary)**	-0.57	-	-	-0.20
**Black race/ethnic group*** (binary)**	0.08	-	0.40	-0.00
**Hispanic race/ethnic group*** (binary)**	-0.65	-0.47	-1.22	-0.50
**American Indian or Alaskan Native race/ethnic group*** (binary)**	0.74	-	0.11	-0.36
**Native Hawaiian or Pacific Islander race/ethnic group*** (binary)**	0.75	-	-0.47	-0.33
**Screening effectiveness (binary)**	-0.18	0.21	-0.45	-0.11
**Screening effectiveness interaction with current smoking status (binary)**	-	-	0.02¶	-
**Screening effectiveness interaction with smoking cessation <5 years ago (binary)**	0.00	-	-	-
**Screening effectiveness interaction with smoking cessation 5-10 years ago (binary)**	-0.04	0.35	-0.05	-0.01
**Screening effectiveness interaction with smoking cessation ≥10 years ago (binary)**	-0.49	-	-	-

Table notes:*compared to male sex, **compared to former smokers, ***compared to a White race/ethnic group. Parameter coefficients were shrunk through penalized (elastic net) logistic regression

¶ relative excess risk due to interaction (RERI): 0.57 (95% confidence interval: -0.58, 1.72; p-value = 0.17). Tests were two-sided based on the Delta method through R-package "InteractionR" (version 0.1.7)..[9,10]

Table S16: Parameter estimates for the effect-model approaches in NELSON for histology-specific mortality (with interactions between screening effectiveness and accumulated pack-years)

Parameter	Adenocarcinoma	Squamous cell carcinoma	Other lung cancers	Small cell carcinoma
Intercept	-7.92	-9.26	-8.25	-8.40
Age (per 1-year increase)	0.05	0.06	0.03	0.03
Female sex (binary)*	-	-0.91	-	-
Personal history of cancer (binary)	-	0.62	-	-0.14
Body-mass index, per 1 unit increase	-0.05	-0.05	-	0.01
Level of education, per 1 unit increase	-0.12	-0.07	-	-0.12
Current smoking status (binary)**	0.18	0.83	-	0.92
Number of cigarettes per day, per 1 unit increase	0.01	0.02	-	0.01
Number of years smoked, per 1 unit increase	0.06	0.03	0.02	0.02
Number of years since smoking cessation, per 1 unit increase	-0.02	0.00	-	-0.03
Screening effectiveness (binary)	-0.17	-0.43	-0.28	-0.27
Screening effectiveness interaction with <30 accumulated pack-years (binary)	-0.32	-0.67	-	-
Screening effectiveness interaction with 30-39 accumulated pack-years (binary)	-	-0.65	-	-
Screening effectiveness interaction with 40-49 accumulated pack-years (binary)	-	-0.35	-	0.34
Screening effectiveness interaction with ≥50 accumulated pack-years (binary)	0.12	-	-	0.38

Table notes:*compared to male sex, **compared to former smokers. Parameter coefficients were shrunk through penalized (elastic net) logistic regression

Table S17: Parameter estimates for the effect-model approaches in NLST for histology-specific mortality (with interactions between screening effectiveness and accumulated pack-years)

Parameter	Adenocarcinoma	Squamous cell carcinoma	Other lung cancers	Small cell carcinoma
**Intercept**	-8.82	-8.54	-10.85	-10.85
**Age (per 1-year increase)**	0.05	0.04	0.06	0.05
**Female sex* (binary)**	-0.13	-0.49	0.01	-
**Personal history of cancer (binary)**	0.65	-0.14	0.35	-0.09
**Body-mass index, per 1 unit increase**	-0.03	-0.04	-0.02	-
**Level of education, per 1 unit increase**	-0.04	-0.15	-0.06	-0.11
**Current smoking status** (binary)**	0.30	0.26	0.22	0.48
**Number of cigarettes per day, per 1 unit increase**	0.01	0.02	0.01	0.02
**Number of years smoked, per 1 unit increase**	0.03	0.04	0.05	0.04
**Number of years since smoking cessation, per 1 unit increase**	0.00	-0.04	-0.03	-0.04
**Chronic obstructive pulmonary disease (binary)**	0.15	0.41	0.39	0.37
**Presence of emphysema (binary)**	0.35	0.45	0.47	0.07
**Family history of lung cancer (binary)**	0.36	-	-	-
**Asian race/ethnic group*** (binary)**	-0.46	-	-	-0.30
**Black race/ethnic group*** (binary)**	0.05	-	0.39	-0.02
**Hispanic race/ethnic group*** (binary)**	-0.53	-0.45	-1.13	-0.65
**American Indian or Alaskan Native race/ethnic group*** (binary)**	0.68	-	0.06	-0.62
**Native Hawaiian or Pacific Islander race/ethnic group*** (binary)**	0.70	-	-0.38	-0.57
**Screening effectiveness (binary)**	-0.18	0.28	-0.44	-0.04
**Screening effectiveness interaction with <30 accumulated pack-years (binary)**	Participants with <30 pack-years were not included in NLST	Participants with <30 pack-years were not included in NLST	Participants with <30 pack-years were not included in NLST	Participants with <30 pack-years were not included in NLST
**Screening effectiveness interaction with 30-39 accumulated pack-years (binary)**	-0.55	-	-	-0.76
**Screening effectiveness interaction with 40-49 accumulated pack-years (binary)**	-	-0.17	-	-
**Screening effectiveness interaction with ≥50 accumulated pack-years (binary)**	-	-	-	-

Table notes:*compared to male sex, **compared to former smokers, ***compared to a White race/ethnic group

Parameter coefficients were shrunk through penalized (elastic net) logistic regression

Table S18: Parameter estimates for the effect-model approaches in NELSON for histology-specific mortality (with interactions between screening effectiveness and sex)

Parameter	Adenocarcinoma	Squamous cell carcinoma	Other lung cancers	Small cell carcinoma
**Intercept**	-8.26	-9.55	-8.72	-8.24
**Age (per 1-year increase)**	0.05	0.05	0.04	0.02
**Female sex (binary)***	-0.02	-0.69	-	-
**Personal history of cancer (binary)**	-	0.59	-	-0.06
**Body-mass index, per 1 unit increase**	-0.05	-0.04	-	0.00
**Level of education, per 1 unit increase**	-0.13	-0.06	-	-0.11
**Current smoking status (binary)****	0.18	0.76	-	0.86
**Number of cigarettes per day, per 1 unit increase**	0.01	0.03	-	0.02
**Number of years smoked, per 1 unit increase**	0.06	0.04	0.02	0.03
**Number of years since smoking cessation, per 1 unit increase**	-0.03	-	-	-0.03
**Screening effectiveness (binary)**	-0.20	-0.70	-0.28	-0.11
**Screening effectiveness interaction with female sex (binary)**	-	-0.48¶	-	-

Table notes:*compared to male sex, **compared to former smokers

¶ relative excess risk due to interaction (RERI): 1.47 (95% confidence interval: -4.55, 7.49; p-value = 0.32)). Tests were two-sided based on the Delta method through R-package "InteractionR" (version 0.1.7)..[9,10]

Parameter coefficients were shrunk through penalized (elastic net) logistic regression

Table S19: Parameter estimates for the effect-model approaches in NLST for histology-specific mortality (with interactions between screening effectiveness and sex)

Parameter	Adenocarcinoma	Squamous cell carcinoma	Other lung cancers	Small cell carcinoma
**Intercept**	-9.00	-8.60	-10.99	-10.88
**Age (per 1-year increase)**	0.05	0.04	0.06	0.05
**Female sex* (binary)**	-0.13	-0.46	0.03	-
**Personal history of cancer (binary)**	0.65	-0.13	0.37	-0.03
**Body-mass index, per 1 unit increase**	-0.03	-0.04	-0.02	-
**Level of education, per 1 unit increase**	-0.04	-0.15	-0.07	-0.10
**Current smoking status** (binary)**	0.29	0.26	0.23	0.46
**Number of cigarettes per day, per 1 unit increase**	0.01	0.02	0.01	0.02
**Number of years smoked, per 1 unit increase**	0.03	0.04	0.05	0.05
**Number of years since smoking cessation, per 1 unit increase**	0.00	-0.04	-0.03	-0.04
**Chronic obstructive pulmonary disease (binary)**	0.15	0.41	0.40	0.35
**Presence of emphysema (binary)**	0.35	0.45	0.48	0.06
**Family history of lung cancer (binary)**	0.36	-	-	-
**Asian race/ethnic group*** (binary)**	-0.44	-	-	-0.20
**Black race/ethnic group*** (binary)**	0.03	-	0.42	-0.00
**Hispanic race/ethnic group*** (binary)**	-0.50	-0.44	-1.38	-0.50
**American Indian or Alaskan Native race/ethnic group*** (binary)**	0.65	-	0.19	-0.36
**Native Hawaiian or Pacific Islander race/ethnic group*** (binary)**	0.66	-	-0.65	-0.33
**Screening effectiveness (binary)**	-0.26	0.26	-0.44	-0.11
**Screening effectiveness interaction with female sex (binary)**	-	-0.05 ¶	-	-0.02 #

Table notes:*compared to male sex, **compared to former smokers, ***compared to a White race/ethnic group

¶ relative excess risk due to interaction (RERI): 0.43 (95% confidence interval: -0.43, 1.29; p-value = 0.17)). Tests were two-sided based on the Delta method through R-package "InteractionR" (version 0.1.7)..[9,10]

# relative excess risk due to interaction (RERI): 0.46 (95% confidence interval: -1.11, 0.19 ; p-value = 0.92)). Tests were two-sided based on the Delta method through R-package "InteractionR" (version 0.1.7)..[9,10]

Parameter coefficients were shrunk through penalized (elastic net) logistic regression

Table S20: Comparison of the lung cancer histology distribution between the NLST control arm and the Prostate, Lung, Colorectal, and Ovarian (PLCO) Cancer Screening Trial.

Histology distribution	NLST Control arm	PLCO Chest x-ray arm* (Oken, JAMA, 2011)
**Adenocarcinoma (includes large-cell carcinoma)**	44.6%	45.6%
**Other non-small cell (Including non small cell cancer, not otherwise specified)**	16.8%	19.8%
**Small cell**	15.7%	13.5%
**Squamous**	21.9%	20.4%
**Histology unknown**	1.0%	0.8%

Table notes: *derived from Table 2 of Oken et al, JAMA, 2011.

Supplementary Figures

Figure S1: Screening effectiveness for overall LCM by methodology and trial

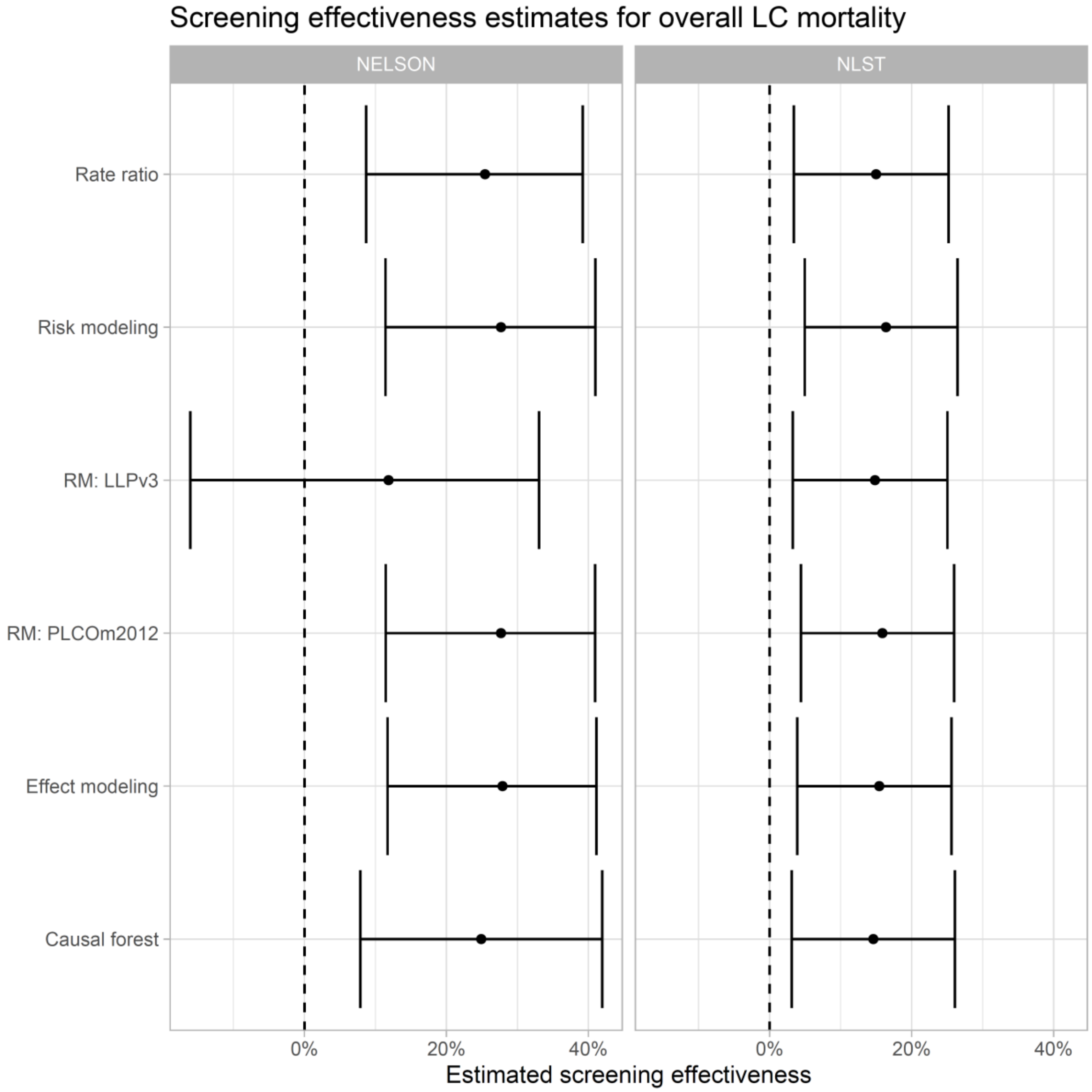

Figure notes: Based on N = 400 lung cancer deaths in NELSON and N = 977 lung cancer deaths in NLST. The NELSON estimate for screening effectiveness for the risk-model approach which uses LLPv3 risk in its first stage includes an interaction-effect between first-stage risk and screening effectiveness The figure represents the estimate for the screening effectiveness parameter without the interaction effect,. The error bars represent the 95% confidence intervals.

Abbreviations: Risk-modelling (RM)

Figure S2: Rate-ratio by risk-prediction model quintiles based on NELSON in NELSON

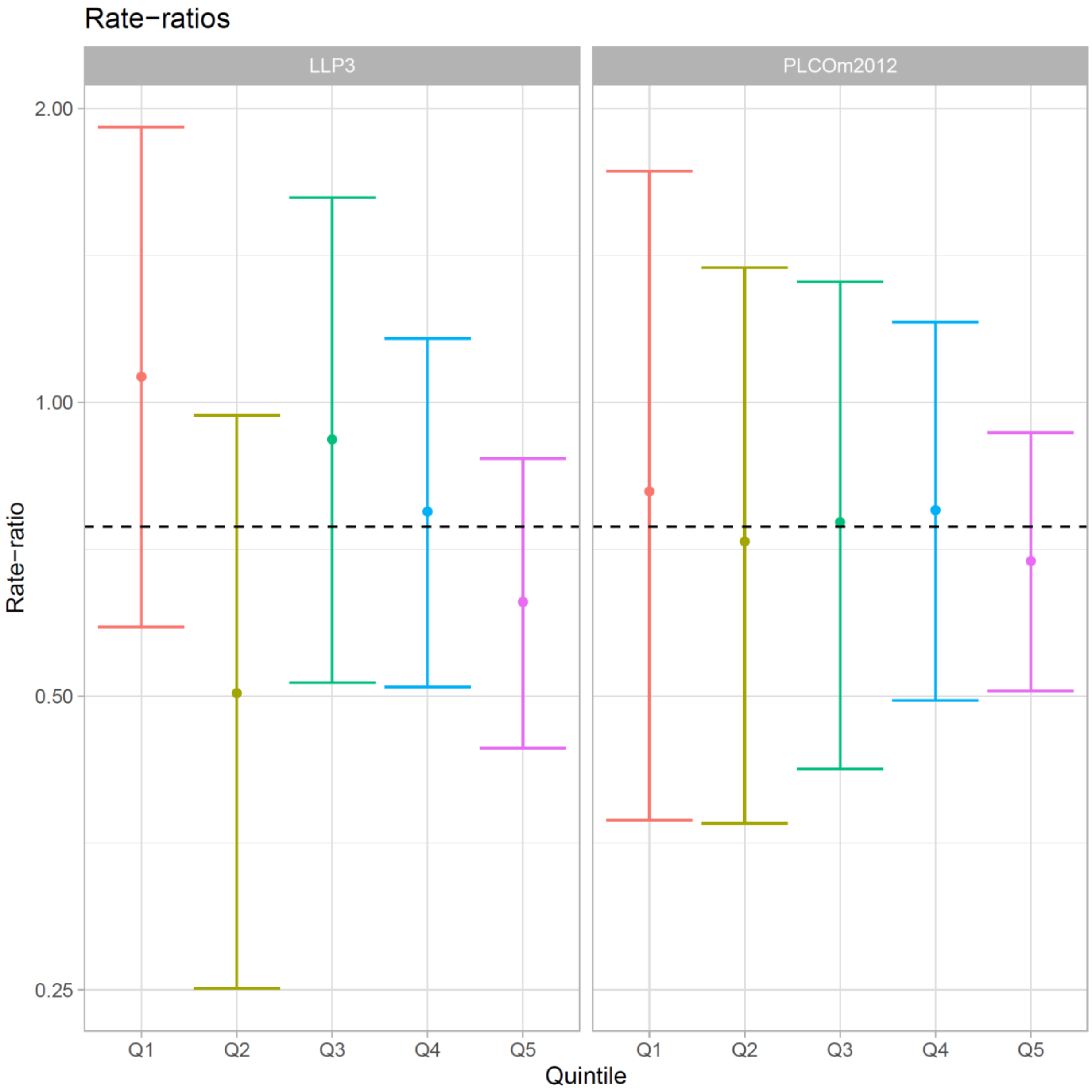

Figure notes: Based on N = 400 lung cancer deaths in NELSON. The y-axis represents a logarithmic scale. The error bars represent the 95% confidence intervals.

Quintile thresholds	Q1	Q2	Q3	Q4	Q5
Risk modelling (LLPv3 model)	<0.27%	0.27% - 0.41%	0.41% - 0.75%	0.75% - 1.73%	>1.73%
Risk modelling (PLCOm2012 model)	<0.96%	0.96% - 1.42%	1.42% - 2.01%	2.01% - 3.02%	>3.02%

Figure S3: Rate-ratio by risk-prediction model quintile based on NLST in NLST

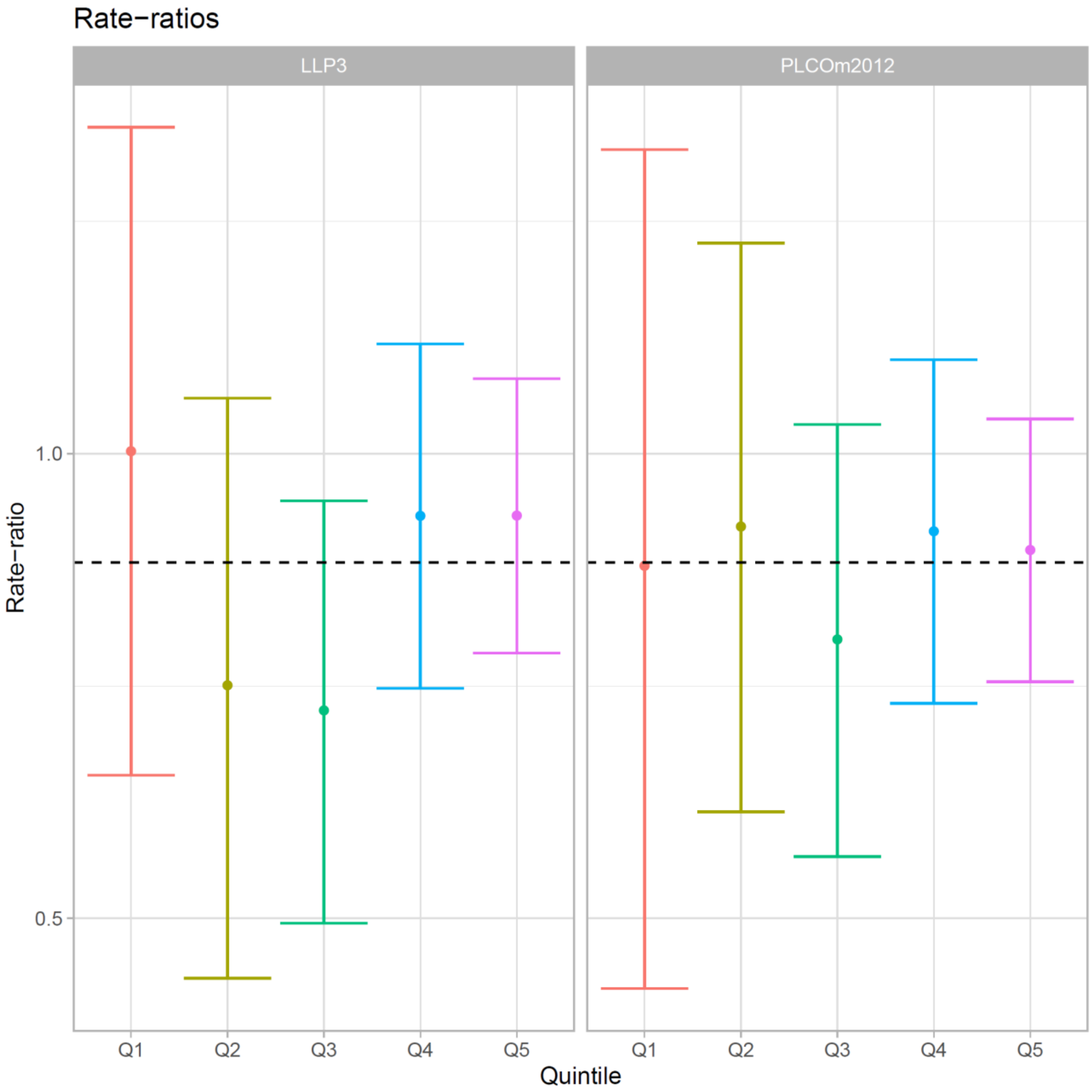

Figure notes: Based on N = 977 lung cancer deaths in NLST. The y-axis represents a logarithmic scale. The error bars represent the 95% confidence intervals.

Quintile thresholds	Q1	Q2	Q3	Q4	Q5
Risk modelling (LLPv3 model)	<0.51%	0.51% - 0.98%	0.98% - 1.61%	1.61% - 2.65%	>2.65%
Risk modelling (PLCOm2012 model)	<1.52%	1.52% - 2.25%	2.25% - 3.25%	3.25% - 5.12%	>5.12%

Figure S4: Rate-ratio by risk-prediction model based on NELSON in NLST

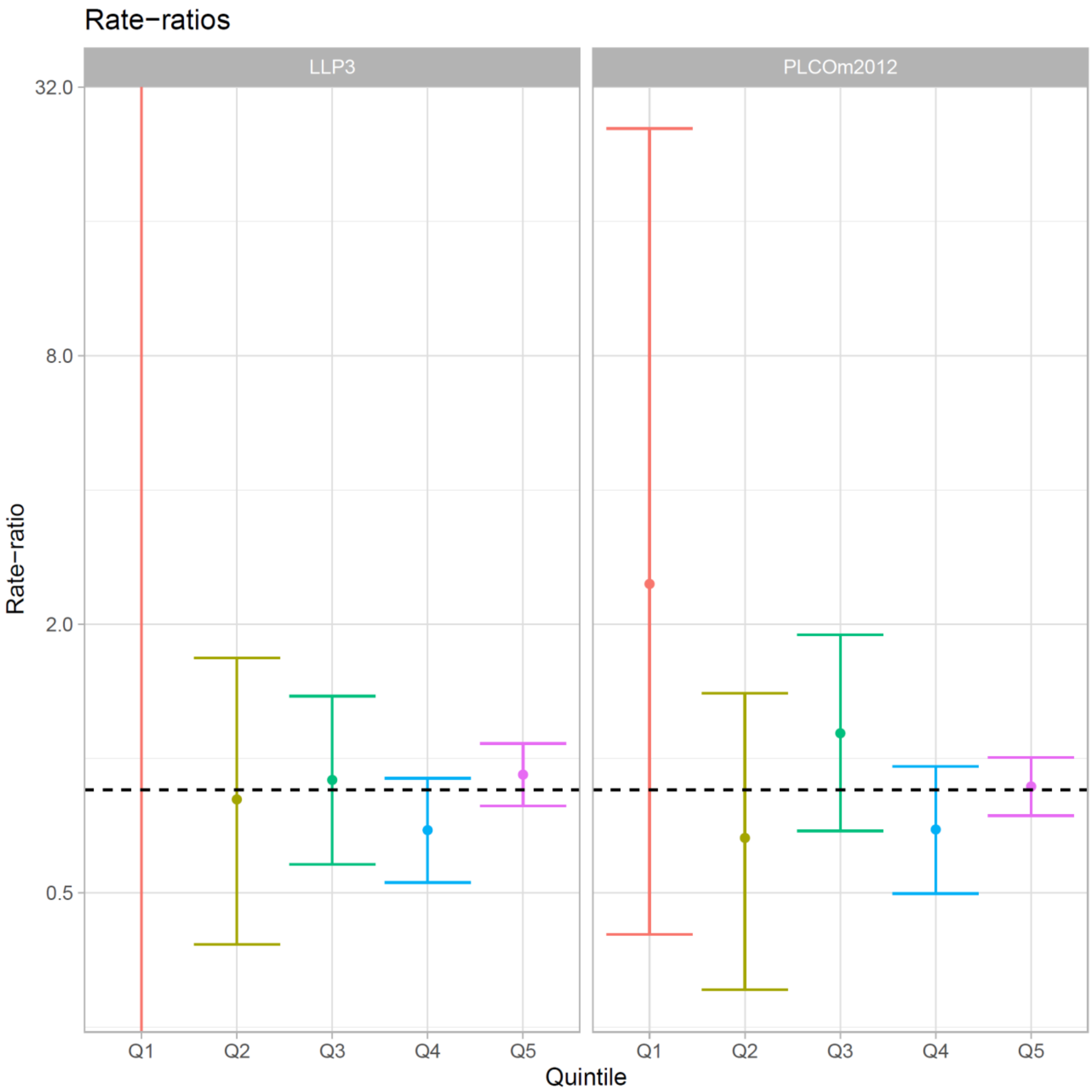

Figure notes: Based on N = 977 lung cancer deaths in NLST. The y-axis represents a logarithmic scale. The error bars represent the 95% confidence intervals.

Quintile thresholds	Q1	Q2	Q3	Q4	Q5
Risk modelling (LLPv3 model)	<0.27%	0.27% - 0.41%	0.41% - 0.75%	0.75% - 1.73%	>1.73%
Risk modelling (PLCOm2012 model)	<0.96%	0.96% - 1.42%	1.42% - 2.01%	2.01% - 3.02%	>3.02%

Figure S5: Rate-ratio by risk-prediction model based on NLST in NELSON

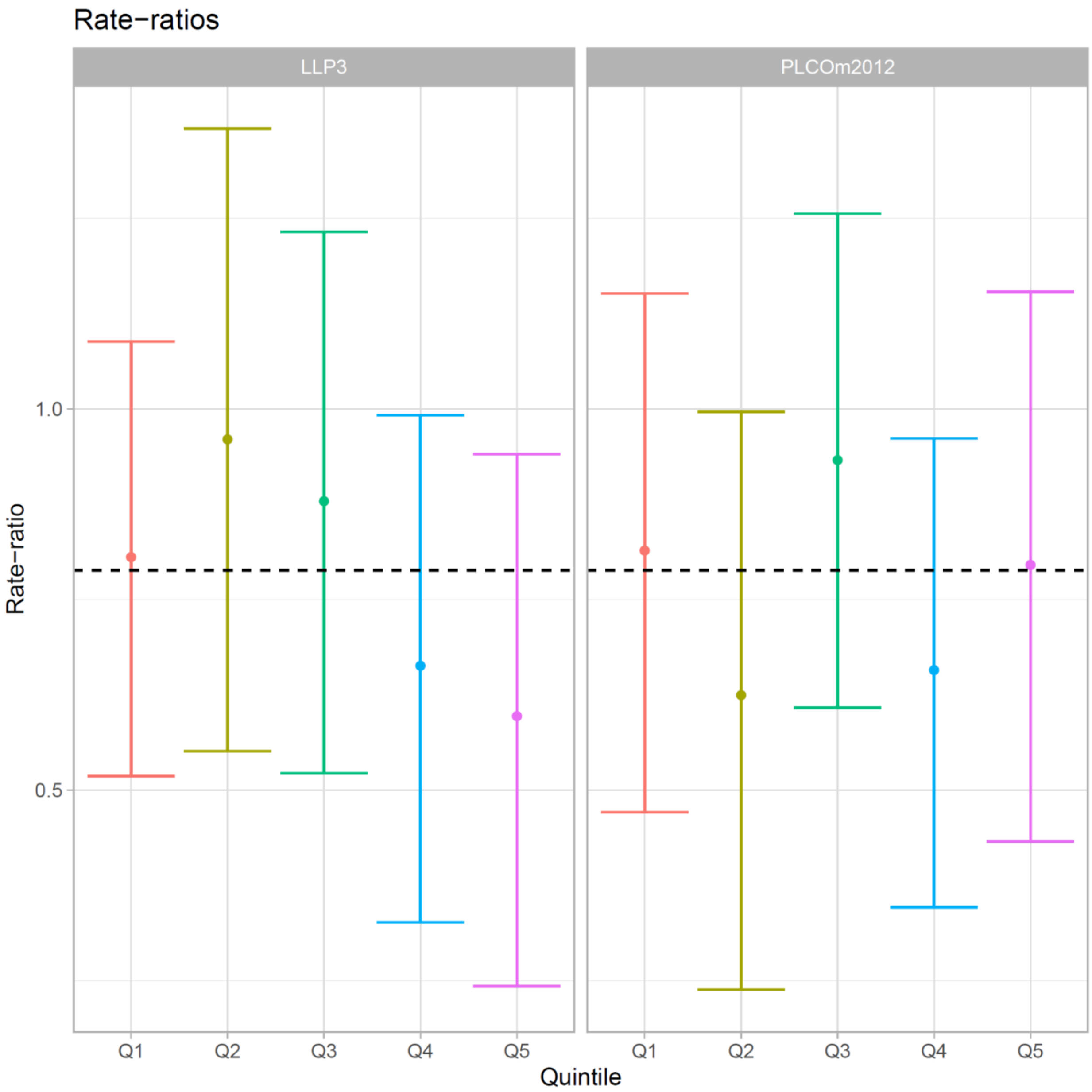

Figure notes: Based on N = 400 lung cancer deaths in NELSON. The y-axis represents a logarithmic scale. The error bars represent the 95% confidence intervals.

Quintile thresholds	Q1	Q2	Q3	Q4	Q5
Risk modelling (LLPv3 model)	<0.51%	0.51% - 0.98%	0.98% - 1.61%	1.61% - 2.65%	>2.65%
Risk modelling (PLCOm2012 model)	<1.52%	1.52% - 2.25%	2.25% - 3.25%	3.25% - 5.12%	>5.12%

Figure S6: Screening effectiveness for overall LCM by age-group

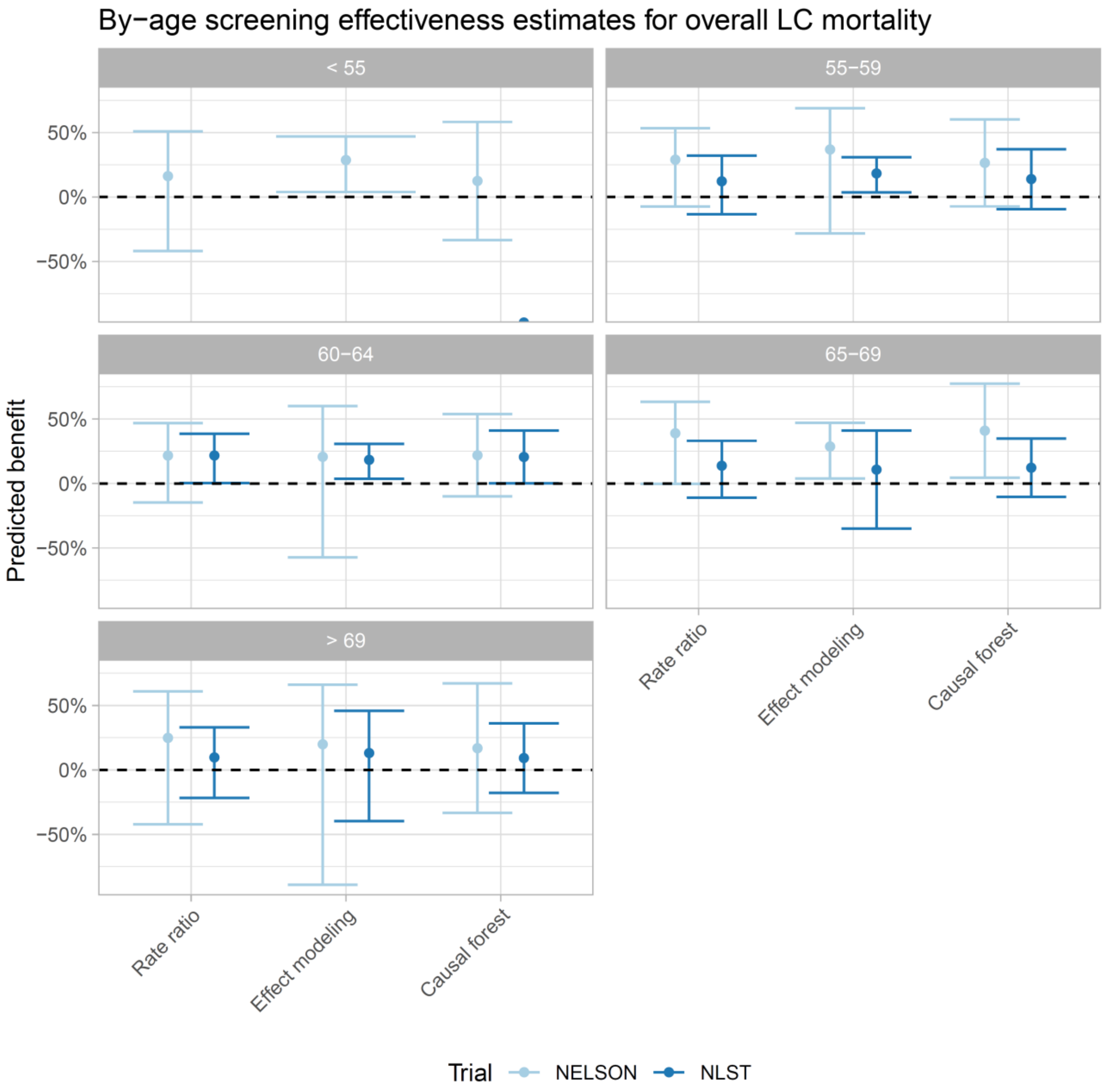

Figure notes: Based on N = 400 lung cancer deaths in NELSON and N = 977 lung cancer deaths in NLST. The error bars represent the 95% confidence intervals.

Figure S7: Screening effectiveness for overall LCM by smoking status

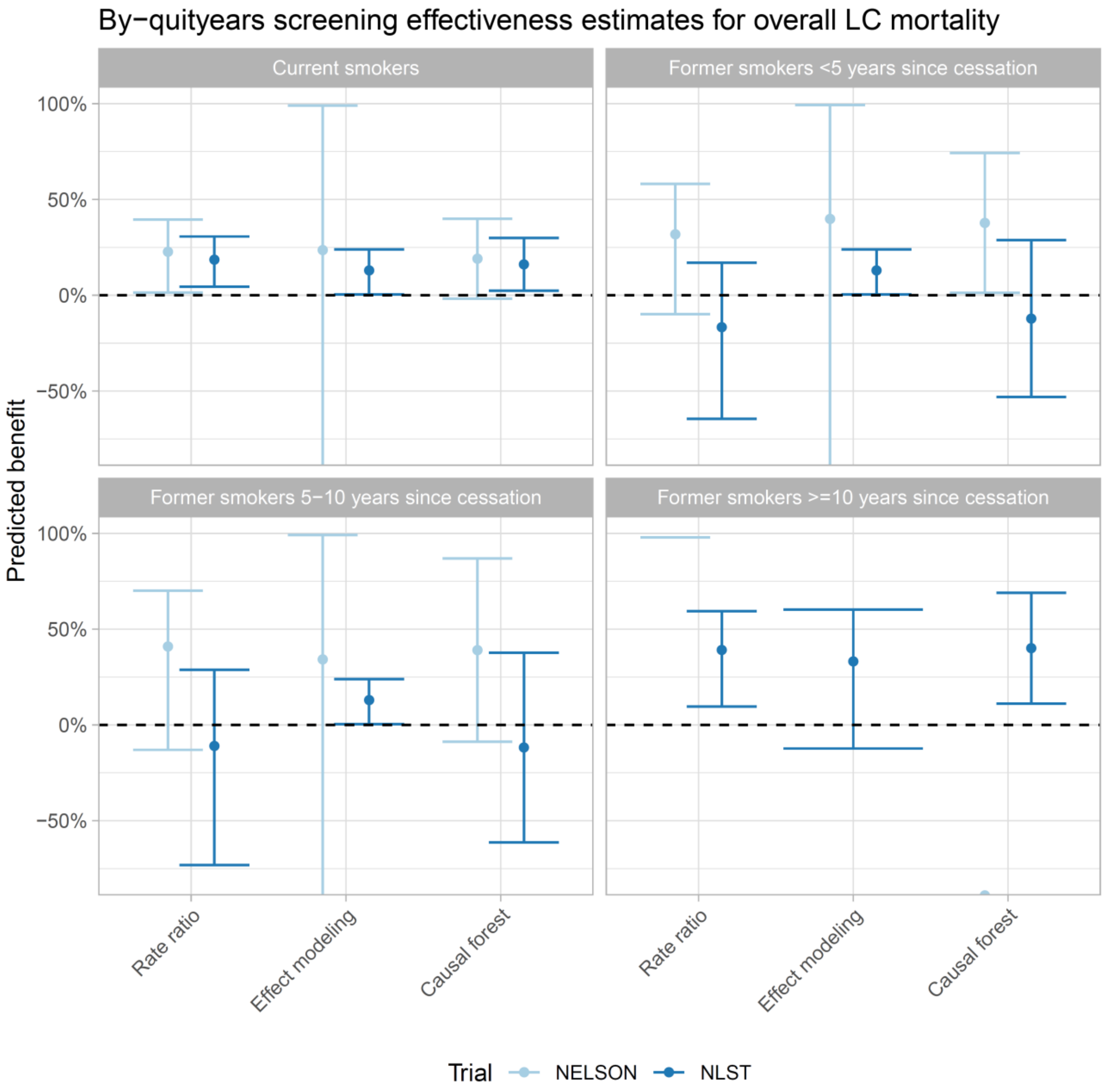

Figure notes: Based on N = 400 lung cancer deaths in NELSON and N = 977 lung cancer deaths in NLST. The range of the plot was restricted because the effect model's lower confidence bounds of NELSON are excessively large. This happens because the penalization in the effect model does not eliminate any of the interactions with the years-since-cessation category groups, leading to strong but imperfect collinearity between the interaction terms, which is a well-known statistical issue resulting in excessively wide confidence intervals. This issue is closely related to overfitting, which is a concern raised in the PATH statement on effect models. However, the PATH statement does not provide guidance on the issue of collinearity resulting from the penalization retaining all interaction terms. We have therefore decided to not adjust the modeling strategy for this particular case, but point out this issue for future methodological research. The error bars represent the 95% confidence intervals.

Figure S8: Screening effectiveness for overall LCM by accumulated pack-years

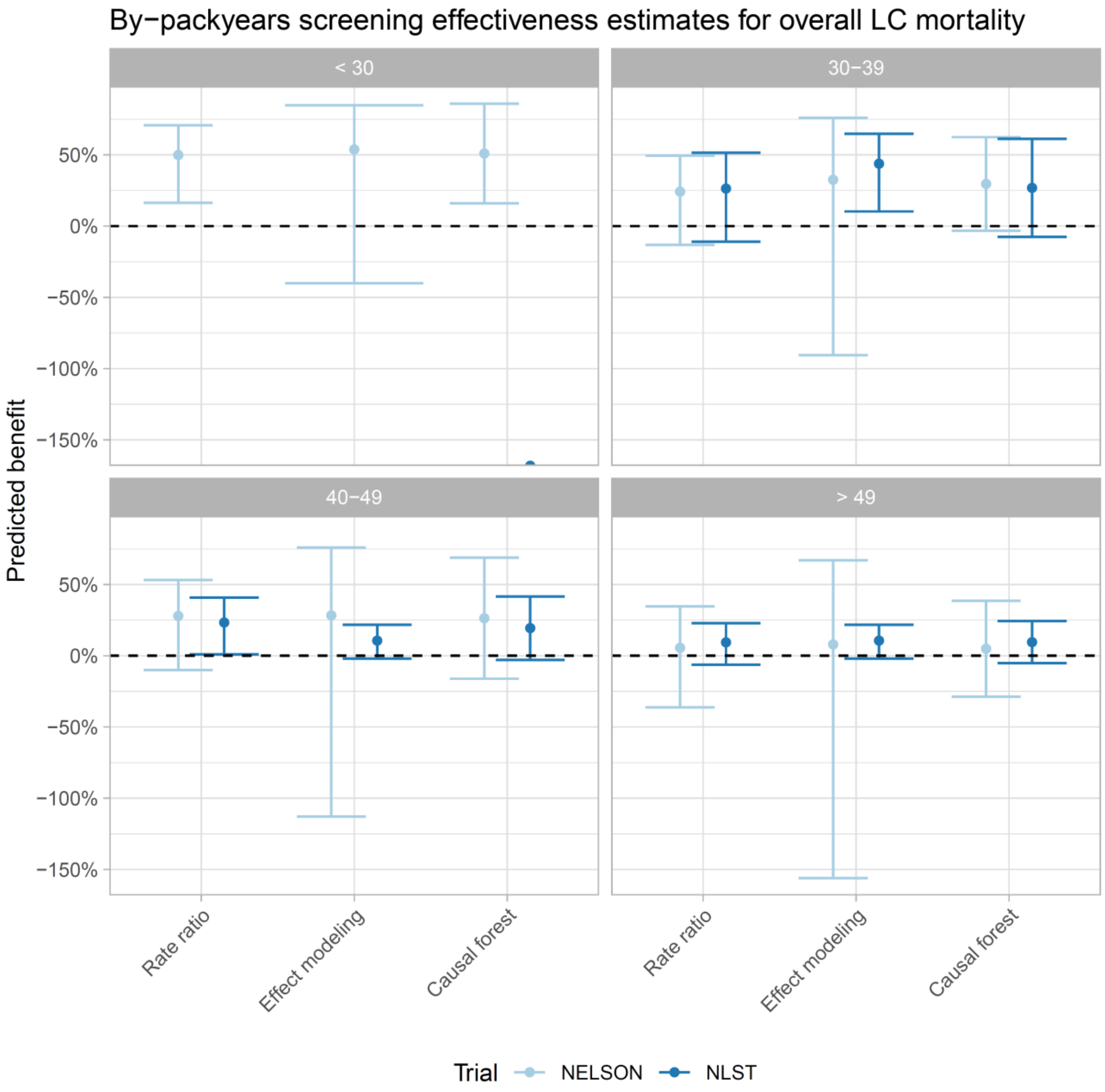

Figure notes: Based on N = 400 lung cancer deaths in NELSON and N = 977 lung cancer deaths in NLST. The error bars represent the 95% confidence intervals.

Figure S9: Screening effectiveness for overall LCM by sex

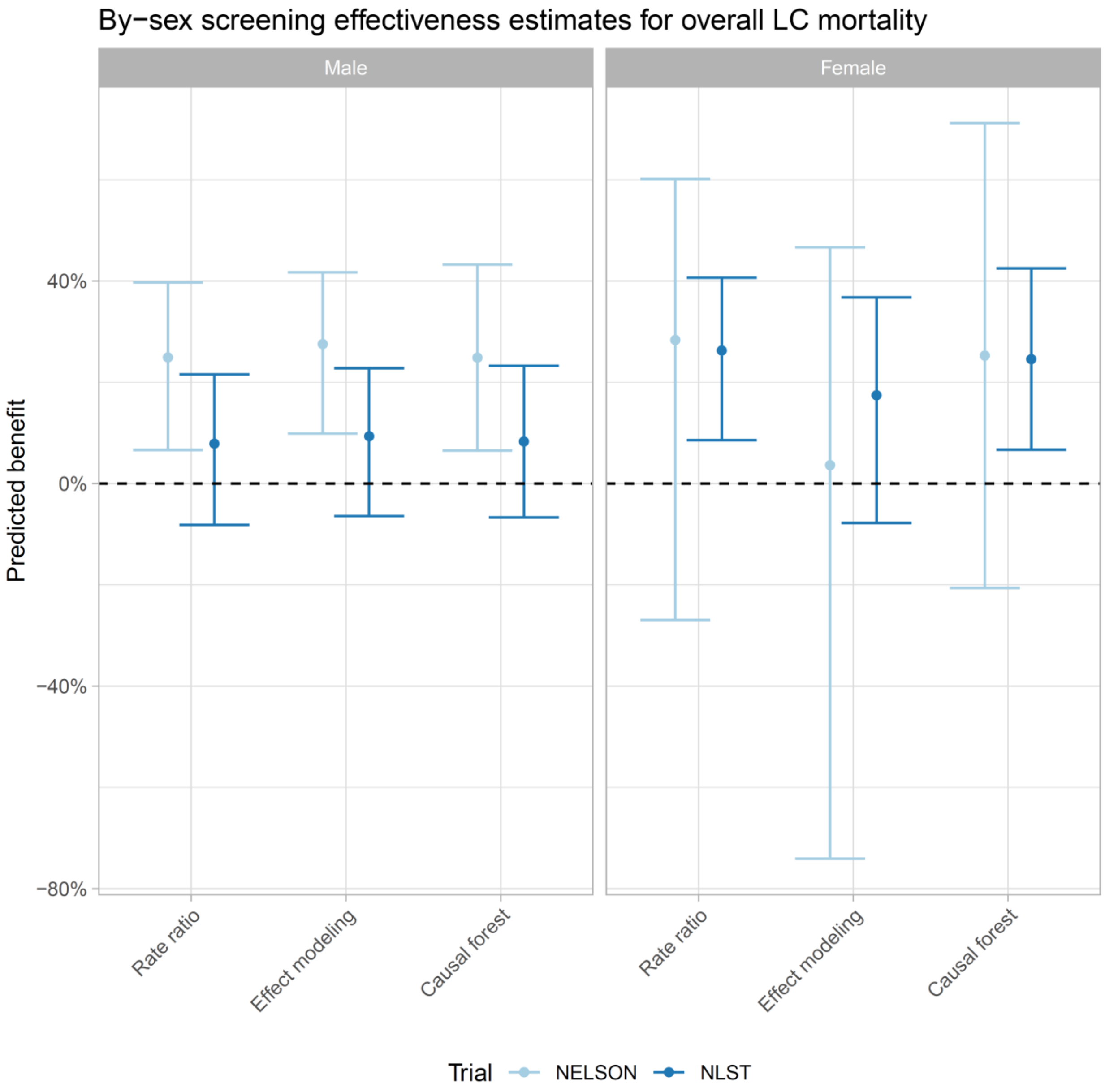

Figure notes: Based on N = 400 lung cancer deaths in NELSON and N = 977 lung cancer deaths in NLST. The error bars represent the 95% confidence intervals.

Figure S10: LLPv3 model estimate distribution by risk-factor in the CT-arms of NELSON and NLST

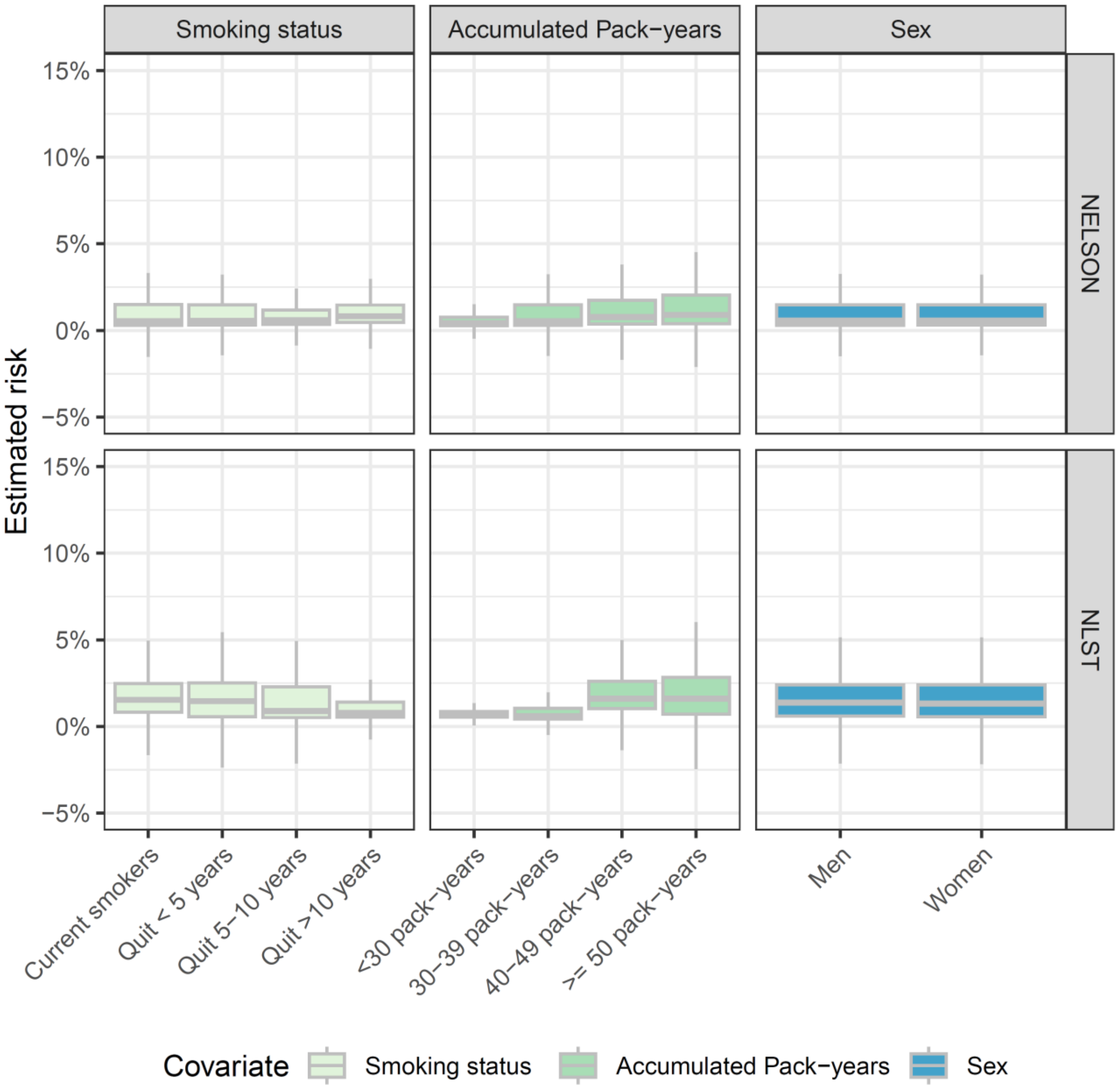

Figure notes: Based on N = 7,401 participants from NELSON and N = 26,701 participants from NLST. Box. Box-plot whisker length represent 1.5 times the interquartile range.

Figure S11: PLCOm2012 model estimate distribution by risk-factor in the CT-arms of NELSON and NLST

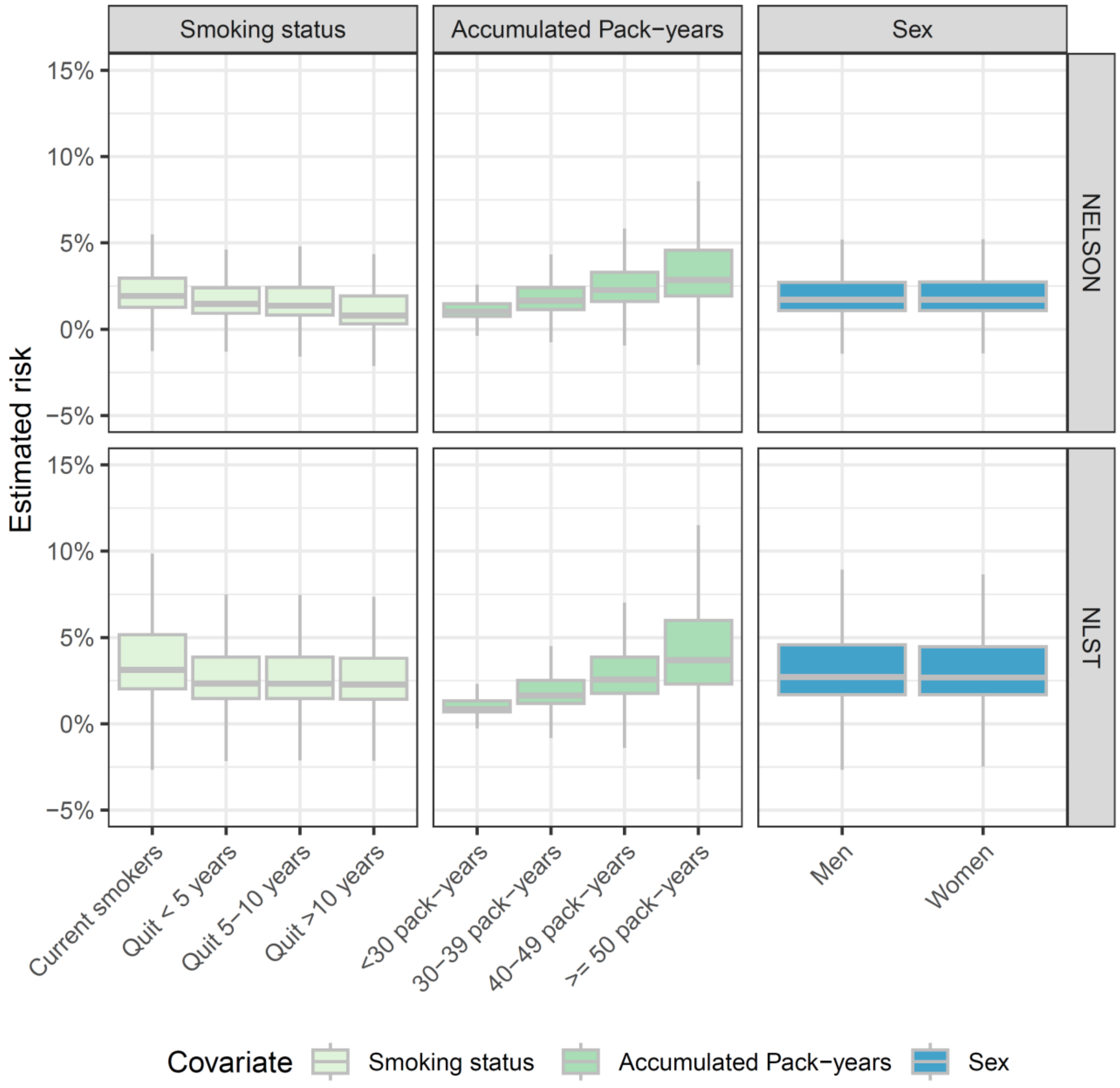

Figure notes: Based on N = 7,401 participants from NELSON and N = 26,701 participants from NLST. Box-plot whisker length represent 1.5 times the interquartile range.

Figure S12: Screening effectiveness for histology-specific mortality by smoking cessation duration

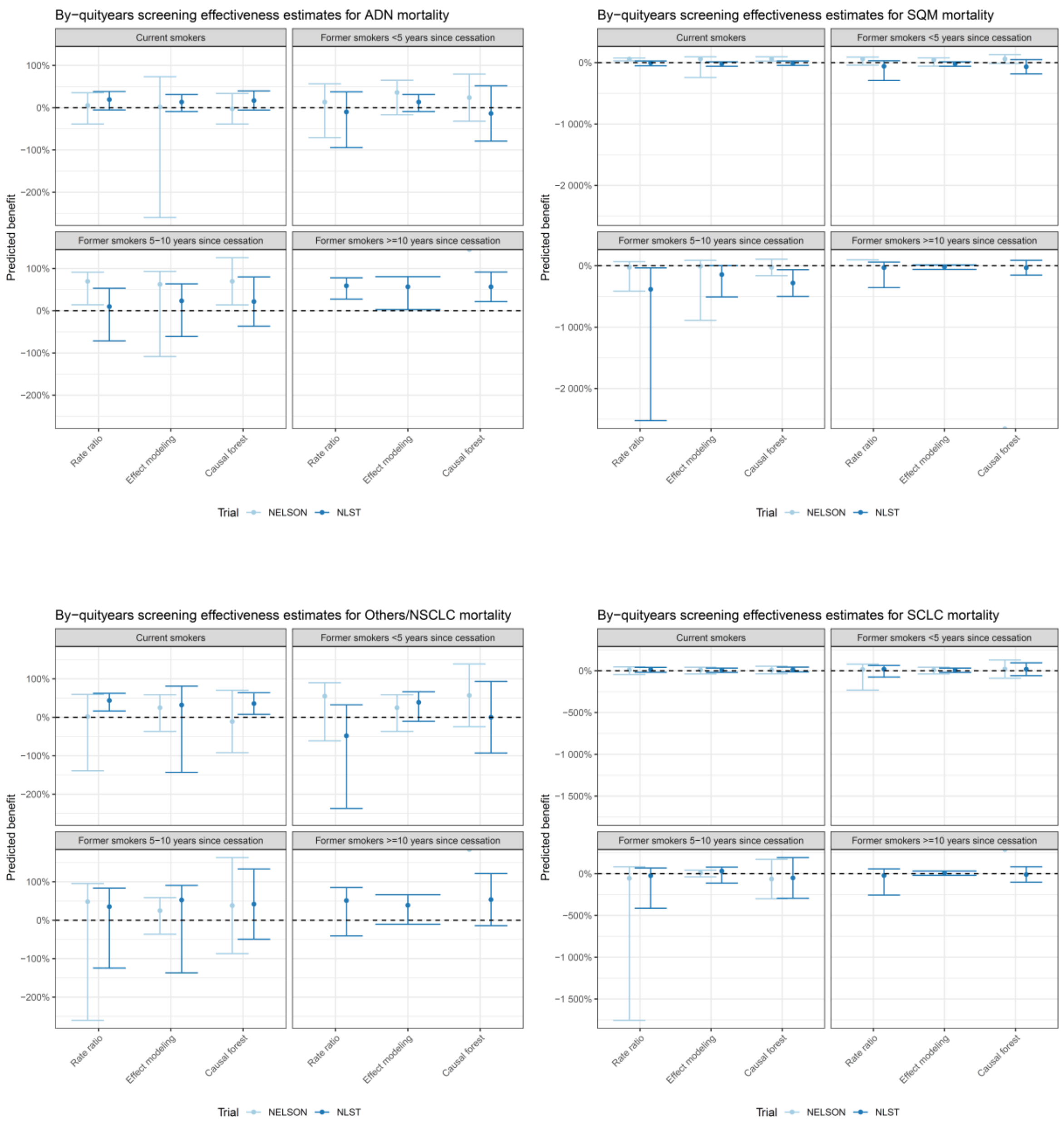

Figure notes: Based on N = 178 Adenocarcinoma deaths, N = 94 Squamous-cell carcinoma deaths, N = 43 Other lung cancer deaths and N = 84 Small-cell carcinoma deaths in NELSON and N = 393 Adenocarcinoma deaths, N = 184 Squamous-cell carcinoma deaths, N = 176 Other lung cancer deaths and N = 209 Small-cell carcinoma deaths in NLST. The error bars represent the 95% confidence intervals.

Abbreviations: Risk-modeling (RM), Adenocarcinoma (ADN), Squamous cell carcinoma (SQM), Other lung cancers (OTH), Small cell carcinoma (SCLC).

Figure S13: Screening effectiveness for histology-specific mortality by accumulated pack-years

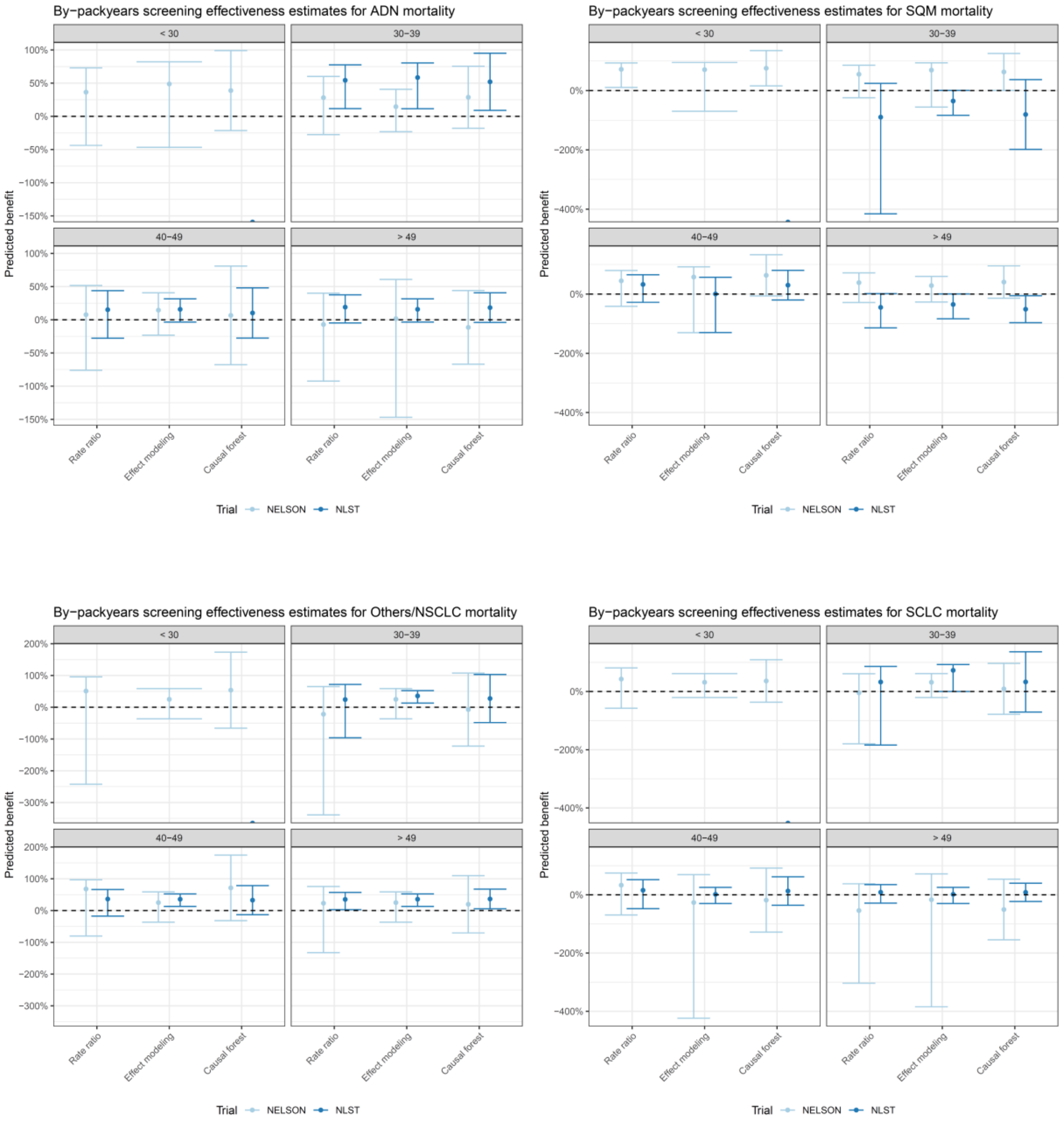

Figure notes: Based on N = 178 Adenocarcinoma deaths, N = 94 Squamous-cell carcinoma deaths, N = 43 Other lung cancer deaths and N = 84 Small-cell carcinoma deaths in NELSON and N = 393 Adenocarcinoma deaths, N = 184 Squamous-cell carcinoma deaths, N = 176 Other lung cancer deaths and N = 209 Small-cell carcinoma deaths in NLST. The error bars represent the 95% confidence intervals.

Abbreviations: Risk-modeling (RM), Adenocarcinoma (ADN), Squamous cell carcinoma (SQM), Other lung cancers (OTH), Small cell carcinoma (SCLC).

Figure S14: Screening effectiveness for histology-specific mortality by sex

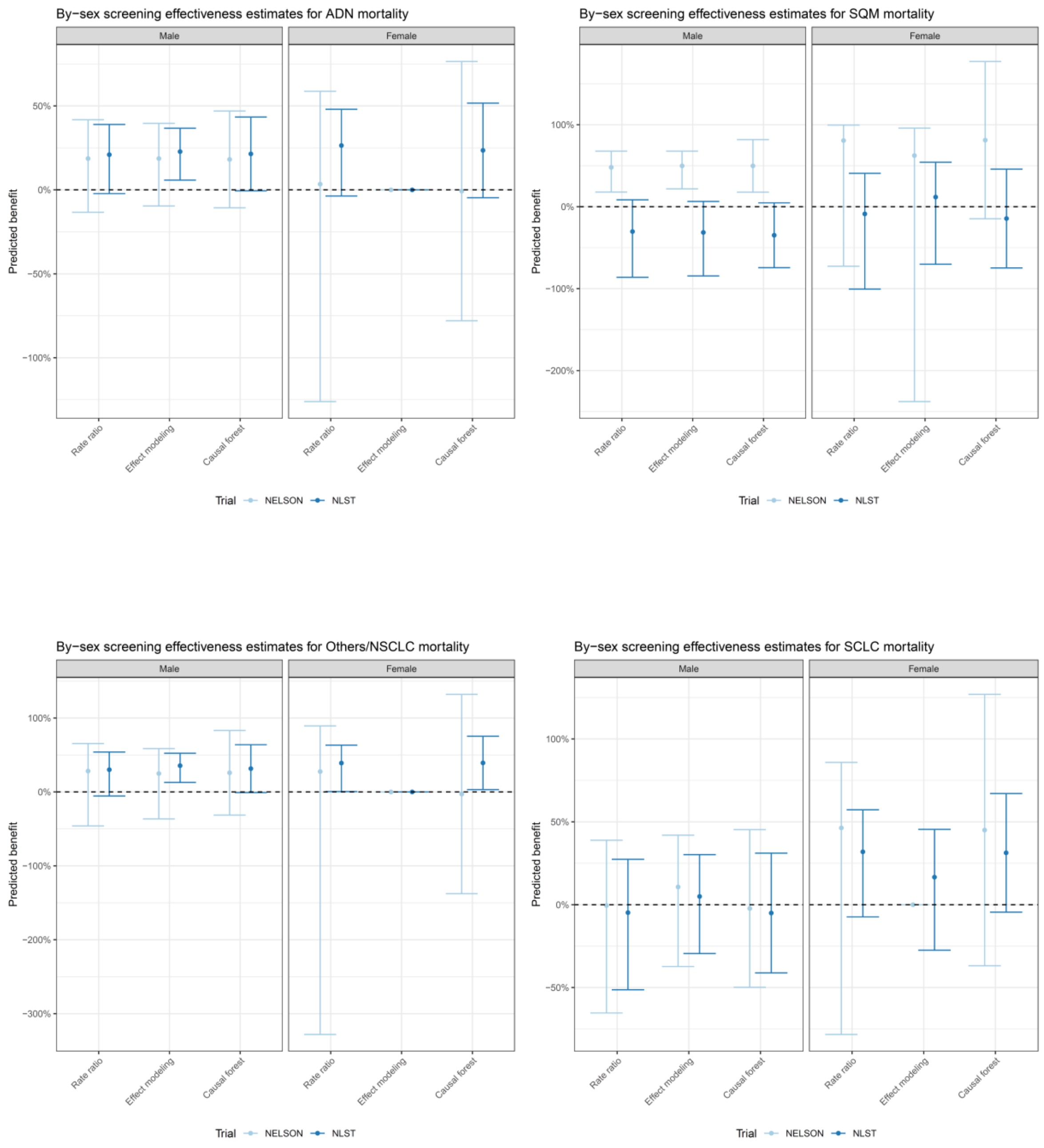

Figure notes: Based on N = 178 Adenocarcinoma deaths, N = 94 Squamous-cell carcinoma deaths, N = 43 Other lung cancer deaths and N = 84 Small-cell carcinoma deaths in NELSON and N = 393 Adenocarcinoma deaths, N = 184 Squamous-cell carcinoma deaths, N = 176 Other lung cancer deaths and N = 209 Small-cell carcinoma deaths in NLST. The error bars represent the 95% confidence intervals.

Abbreviations: Risk-modeling (RM), Adenocarcinoma (ADN), Squamous cell carcinoma (SQM), Other lung cancers (OTH), Small cell carcinoma (SCLC).

Figure S15: Stage distribution of screen-detected cases by histology in NELSON and NLST

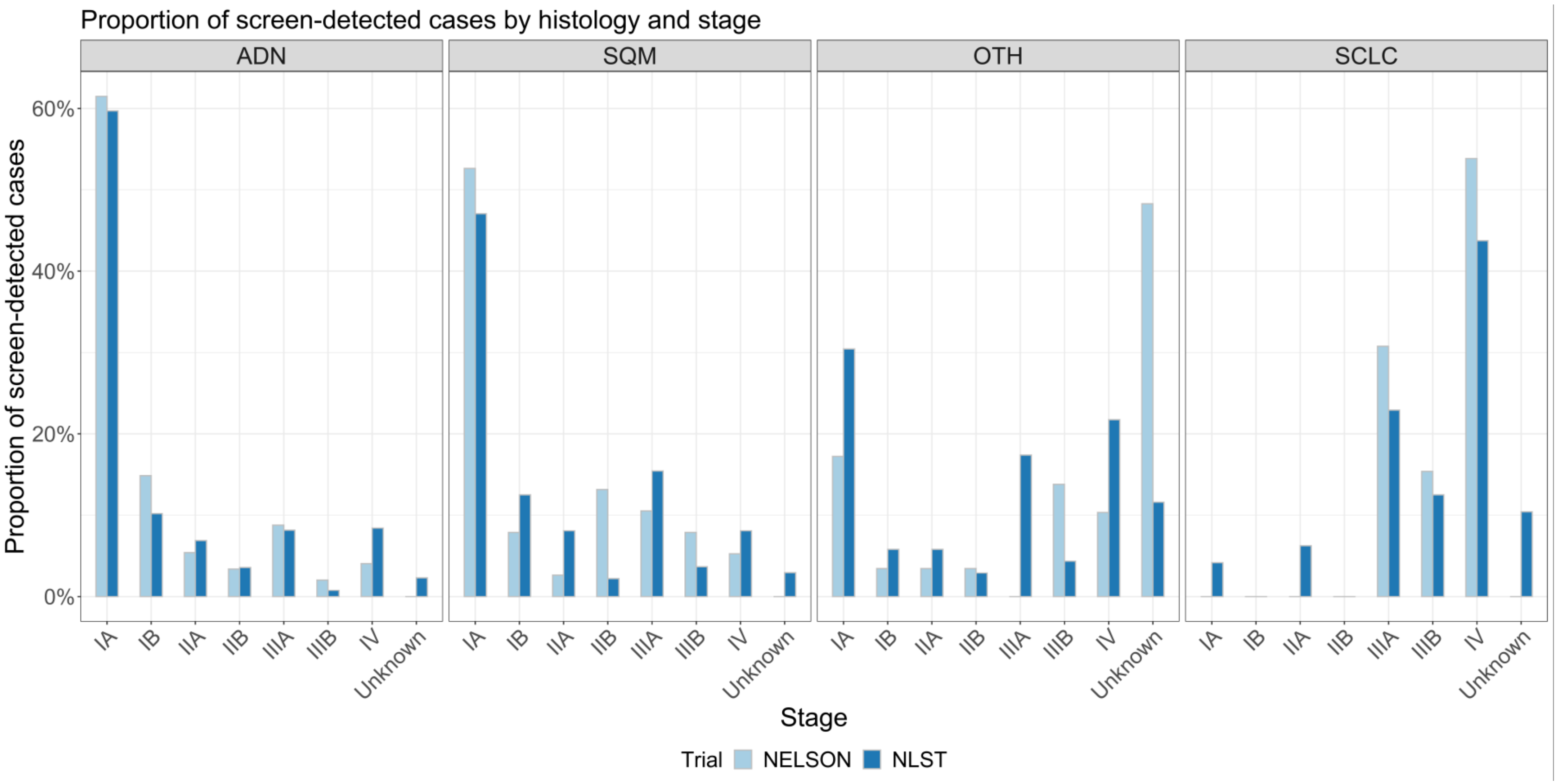

Figure notes: Based on N = 228 screen-detected cases in NELSON and N = 645 screen-detected cases in NLST. None of the screen-detected adenocarcinoma, squamous cell carcinoma and small cell carcinoma had an unknown stage in NELSON. In NELSON, none of the screen-detected other non-small cell cancers were detected in stage IIA. Similarly, none of the screen-detected small cell cancers in NELSON were detected in stages IA, IB, IIA or IIB. Similarly, none of the screen-detected small cell cancers in NLST were detected in stages IB or IIB.

Abbreviations: Adenocarcinoma (ADN), Squamous cell carcinoma (SQM), Other lung cancers (OTH), Small cell carcinoma (SCLC).

Figure S16: Screening effectiveness for histology-specific mortality by smoking status

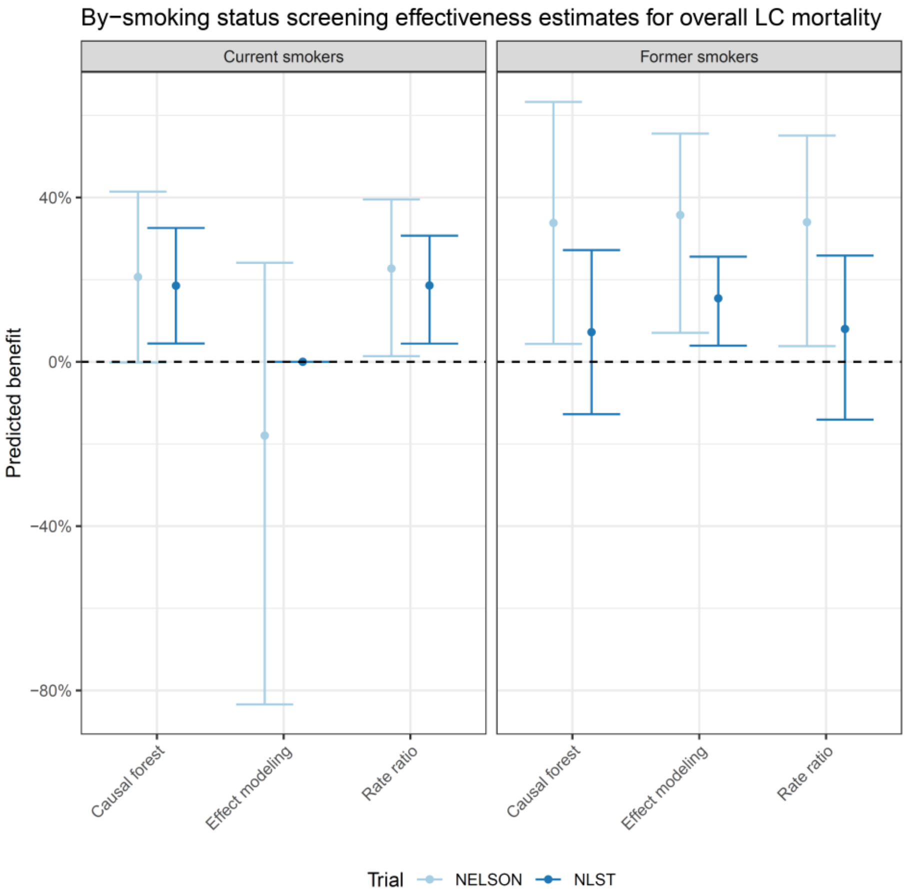

Figure notes: Based on N = 400 lung cancer deaths in NELSON and N = 977 lung cancer deaths in NLST. The error bars represent the 95% confidence intervals.

Data harmonisation

Reported average numbers of cigarettes smoked per day above 100 were considered implausible and recoded as 100 cigarettes per day (NELSON: 0 individuals, NLST: 11 individuals). Furthermore, body mass index (BMI) values less than 14 kg/m2 and over 60 kg/m2 were considered implausible for enrollment in both trials and recoded as 14 kg/m2 (NELSON: 0 individuals, NLST: 5 individuals) and 60kg/m2 (NLST: 4 individuals), respectively. Education in both NELSON and NLST were harmonized to the categories used in the PLCOm2012 model, due to its broad use in international screening recommendations, screening pilots, and clinical trials.[6,11-13] Table 24 shows how the educational categories used in NELSON and NLST were reclassified to the PLCOm2012 categories.

Table S21: Mapping of the educational categories measured in NELSON and NLST to the PLCOm2012 classifications

NELSON educational levels	NLST educational levels	PLCOm2012 classification
“Primary school”, “Lower vocational education”, “Lower general secondary education”	"8th grade or less", "9th-11th grade"	Less than high-school graduate
"Middle-level vocational education"	"High school graduate/GED"	High-school graduate
“Pre-university education”	"Post high school training, excluding college"	Some training after high school
-	"Associate degree/ some college"	Some college
“Higher Vocational Education”	"Bachelors Degree"	College graduate
“University education”	"Graduate School"	Postgraduate or professional degree

Lung cancer histology was harmonized between the two trials based on the International Classification of Diseases for Oncology, Third Edition (ICD-O-3) codes.[14] An overview of the histology classifications by ICD-O-3 code is given in Table S25.

Table S22: Classification of histology categories by ICD-O-3 codes

Histology classification	Adenocarcinoma	Squamous cell carcinoma	Other lung cancers	Small cell carcinoma
**Corresponding ICD-O-3 codes**	8012, 8013, 8140, 8200, 8250, 8251, 8252, 8253, 8254, 8255, 8260, 8310, 8323, 8480, 8481, 8490, 8550, 8560, 8570, 8574	8052, 8070, 8071, 8072, 8075, 8083, 8084	8000, 8001, 8010, 8020, 8021, 8022, 8032, 8033, 8046, 8050, 8240, 8244, 8246, 8249, 8980, 9680, 9699	8041, 8042, 8044, 8045

Risk-prediction models

Description of the Liverpool Lung Project model version 3 (LLPv3)

The Liverpool Lung Project model version 3 (LLPv3) was based on data from the Liverpool Lung Project case-control study.[15] The model was estimated through multivariable conditional logistic regression and predicts lung cancer incidence for a 5-year timeframe. The risk factors incorporated in the model are listed in Table S23, along with their corresponding model coefficients.

As the model intercept could not be estimated using case-control data, the authors derived the age-group and sex-specific model intercepts based on national data from the U.K. The age-group and sex-specific model intercepts are shown in Table S24. The model intercept for an individual aged *x*+*y* years, where *x* is a multiple of 5 and *y* is 0, 1, 2, 3 or 4, can be calculated as follows:

$$Intercept_{Age\ x+y,sex} = \frac{(5-y-0.5)*Intercept_{Age\ x,sex}+(y+0.5)*Intercept_{Age\ x+5,sex}}{5}$$

Table S23: Risk factors considered in the LLPv3 model

Risk factor	Model coefficient
Smoking duration	
Never	0.000 (reference)
1-20 years	0.7692
21-40 years	1.4516
41-60 years	2.5072
>60 years	2.7243
Prior diagnosis of pneumonia or other lung conditions	
No	0.000 (reference)
Yes	0.6025
Asbestos exposure	
No	0.000 (reference)
Yes	0.6343
Personal history of cancer	
No	0.000 (reference)
Yes	0.6754
Family history of lung cancer	
No	0.000 (reference)
Early onset (age < 60 years)	0.7034
Late onset (age ≥ 60 years)	0.1677

Table S24: LLPv3 model intercepts

	Males	Females
**Age-group**	**Corresponding model intercept**	**Corresponding model intercept**
40-44	-9.84	-10.37
45-49	-8.94	-8.53
50-54	-8.09	-7.93
55-59	-7.41	-6.97
60-64	-6.75	-6.69
65-69	-6.34	-6.46
70-74	-6.09	-5.96
75-79	-5.61	-5.70
80-84	-5.46	-5.89

Description of the PLCOm2012 model

The PLCOm2012 model was developed in the control-arm of the Prostate, Lung, Colorectal and Ovarian Cancer Screening Trial (PLCO).[6] The model was estimated through multivariable logistic regression and predicts lung cancer incidence for a six-year timeframe. The model predictors include seven non-smoking variables: age in years, race/ethnicity, education (an estimator of socioeconomic circumstance), body mass index, personal history of cancer, family history of lung cancer, and chronic obstructive pulmonary disease. The model includes four smoking variables: smoking status (former vs. current), smoking intensity (cigarettes per day), smoking duration in years, and yeas since smoking cessation in former smokers. Using multivariable fractional polynomials, smoking intensity was shown to have a nonlinear relationship with lung cancer, and this nonlinear effect is incorporated into PLCOm2012. The risk-factors incorporated in the model are listed in Table S25, along with their log odds ratios and corresponding model coefficients.

Table S25: Risk-factors considered in the PLCOm2012 model

Risk-factor	Log odds ratio	Model coefficient
Age, per one-year increase (centred on age 62)	1.081	0.0778868
Race or ethnic group (self-reported)		
White (non-Hispanic)	1.00 (reference)	0.000 (reference)
Black (non-Hispanic)	1.484	0.3944778
Hispanic	0.475	−0.7434744
Asian	0.627	−0.466585
Native Hawaiian or Pacific Islander	1.00	0.000
American Indian or Alaskan Native	2.793	1.027152
Education, per increase of 1 level. Education was centred on level 4*	0.922	−0.0812744
Body-mass index, per 1-unit increase (centred on 27)	0.973	−0.0274194
Chronic obstructive pulmonary disease		
No	1.00 (reference)	0.000 (reference)
Yes	1.427	0.3553063
Personal history of cancer		
No	1.00 (reference)	0.000 (reference)
Yes	1.582	0.4589971
Family history of lung cancer		
No	1.00 (reference)	0.000 (reference)
Yes	1.799	0.587185
Smoking status		
Former	1.00 (reference)	0.000 (reference)
Current	1.297	0.2597431
Smoking intensity†	‡	−1.822606
Smoking duration in years, per 1-year increase (centred on 27 years)	1.032	0.0317321
Years since smoking cessation, per 1-year increase (centred on 10 years)	0.970	−0.0308572
Model constant		−4.532506

* Education was measured in six ordinal levels: less than high-school graduate (level one), high-school graduate (level two), some training after high school (level three), some college (level four), college graduate (level five), and postgraduate or professional degree (level six).

† For smoking intensity, the contribution of the variable to the model should be calculated by dividing the number of cigarettes per day by 10, exponentiating by the power −1, centering by subtracting 0.4021541613, and multiplying this number by the beta coefficient of the variable.

‡ Due to the transformation of smoking intensity as described above, no single odds ratio represents the entire association

C-Outcomes

C-statistics and C-for-benefit

All methodologies had similar C-statistics (medians: NELSON: 0.6939, NLST: 0.6999) and C-for-benefits (medians: NELSON: 0.4651, NLST: 0.4991) for overall LCM in their development datasets (Supplementary Table S26). External validation yielded similar C-statistics (medians: NELSON: 0.6935, NLST: 0.6995) and C-for-benefits (medians: NELSON: 0.4761, NLST: 0.4896) (Supplementary Table S29).

All methodologies generally yielded similar C-statistics (medians across histologies: NELSON: 0.6493-0.7130; NLST: 0.6856-0.7167) and C-for-benefits (medians across histologies: NELSON: 0.4191-0.4949; NLST: 0.4513-0.6923) for histology-specific LCMs (Supplementary Tables S27-S28). In external validation of histology-specific models, C-statistics generally decreased compared to their development datasets (Supplementary Tables S29-S30), while C-for-benefits changed little, with the exception of squamous-cell carcinoma.

Calibration-for-benefit

Calibration-for-relative-benefits by quintile of baseline risk for overall LCM was good in NELSON and reasonable in NLST for risk- and effect-models (Supplementary Figures C1-C2), while causal forests showed poor calibration (Supplementary Figure C3). For histology-specific benefits, risk-models and effect-models showed poor to acceptable calibration (Supplementary Figures C4-C11), while causal forests showed poor calibration (Supplementary Figures C12-C15).

Absolute benefits increased with quintile of baseline risk for all risk-prediction models (Supplementary Figures C16-C19). Risk- and effect-models showed good calibration-for-absolute-benefits for overall and histology-specific LCM (Supplementary Figures C20-C29). In contrast, causal forests showed good calibration in NELSON and poor calibration in NLST for overall LCM; calibration for histology-specific LCM was poor in both (Supplementary Figures C30-C34).

External validation yielded poor calibration for both relative (Supplementary Figures C35-C44) and absolute benefits (Supplementary Figures C45-C54) for both overall and histology-specific LCM.

Table S26: C-statistics and C-for-benefit for the risk- and effect-modelling approaches for overall LCM

	Risk modelling (first stage based on individual risk-factors)	Risk modelling (first stage based on LLPv3 model)	Risk modelling (first stage based on PLCOm2012 model)	Effect modelling	Causal forests
**Development in NELSON**					
**C-statistic for lung cancer mortality (95% CI)**	0.7025 (0.6504-0.7546)	0.6490 (0.5917-0.7063)	0.6862 (0.6322-0.7402)	0.7078 (0.6568-0.7587)	Method does not allow calculation of the C-statistic for lung cancer mortality
**NELSON C-for-benefit (95% CI)**	0.4632 (0.3987-0.5300)	0.4604 (0.3949-0.5259)	0.4674 (0.4017-0.5332)	0.4651 (0.3993-0.5310)	0.4646 (0.4008-0.5285)
**Validation in NLST**					
**C-statistic for lung cancer mortality (95% CI)**	0.7056 (0.6735-0.7378)	0.6732 (0.6406-0.7059)	0.6951 (0.6629-0.7273)	0.7109 (0.6796-0.7423)	Method does not allow external validation
**NLST C-for-benefit (95% CI)**	0.4888 (0.4475-0.5301)	0.4966 (0.4571-0.5361)	0.4876 (0.4471-0.5280)	0.4898 (0.4484-0.5311)	Method does not allow external validation
**Development in NLST**					
**C-statistic for lung cancer mortality (95% CI)**	0.7203 (0.6891-0.7516)	0.6767 (0.6441-0.7093)	0.6951 (0.6629-0.7274)	0.7244 (0.6938-0.7550)	Method does not allow calculation of the C-statistic for lung cancer mortality
**NLST C-for-benefit (95% CI)**	0.4921 (0.4501-0.5340)	0.4966 (0.4571-0.5361)	0.4853 (0.4447-0.5258)	0.4945 (0.4528-0.5362)	0.4985 (0.4562-0.5408)
**Validation in NELSON**					
**C-statistic for lung cancer mortality (95% CI)**	0.6997 (0.6474-0.7520)	0.6493 (0.5921-0.7064)	0.6862 (0.6322-0.7402)	0.7019 (0.6796-0.723)	Method does not allow external validation
**NELSON C-for-benefit (95% CI)**	0.4649 (0.3995-0.5303)	0.4604 (0.3948-0.5259)	0.4674 (0.4017-0.5332)	0.4898 (0.4484-0.5311)	Method does not allow external validation

Table S27: C-statistics and C-for-benefit for the risk- and effect-modelling approaches for histology specific mortality developed in NELSON

Adenocarcinoma					
	**Risk modelling (individual risk-factors)**	**Risk modelling (LLPv3 model)**	**Risk modelling (PLCOm2012 model)**	**Effect modelling**	**Causal forests**
**C-statistic for lung cancer mortality (95% CI)**	0.7153 (0.6439-0.7886)	0.6588 (0.5750-0.7427)	0.6976 (0.6225-0.7727)	0.7177 (0.6470-0.7884)	Method does not allow calculation of the C-statistic for lung cancer mortality
**C-for-benefit (95% CI)**	0.4916 (0.3951-0.5881)	0.4858 (0.3865-0.5850)	0.4959 (0.4006-0.5912)	0.4912 (0.4949-0.5875)	0.4949 (0.3952-0.5947)
**Squamous cell carcinoma**					
	**Risk modelling (individual risk-factors)**	**Risk modelling (LLPv3 model)**	**Risk modelling (PLCOm2012 model)**	**Effect modelling**	**Causal forests**
**C-statistic for lung cancer mortality (95% CI)**	0.7198 (0.6126-0.8270)	0.6624 (0.5528-0.7720)	0.6964 (0.5865-0.8064)	0.7452 (0.6477-0.8426)	Method does not allow calculation of the C-statistic for lung cancer mortality
**C-for-benefit (95% CI)**	0.4081 (0.2780-0.5382)	0.4404 (0.3148-0.5659)	0.4145 (0.2827-0.5463)	0.4074 (0.2774-0.5373)	0.4913 (0.3649-0.6176)
**Other lung cancers**					
	**Risk modelling (individual risk-factors)**	**Risk modelling (LLPv3 model)**	**Risk modelling (PLCOm2012 model)**	**Effect modelling**	**Causal forests**
**C-statistic for lung cancer mortality (95% CI)**	0.6642 (0.4939-0.8345)	0.6426 (0.4561-0.8291)	0.6679 (0.4982-0.8376)	0.6632 (0.4935-0.8329)	Method does not allow calculation of the C-statistic for lung cancer mortality
**C-for-benefit (95% CI)**	0.4649 (0.2795-0.6502)	0.4299 (0.2349-0.6248)	0.4976 (0.2827-0.6765)	0.4606 (0.2743-0.6470)	0.4484 (0.2252-0.6415)
**Small cell carcinoma**					
	**Risk modelling (individual risk-factors)**	**Risk modelling (LLPv3 model)**	**Risk modelling (PLCOm2012 model)**	**Effect modelling**	**Causal forests**
**C-statistic for lung cancer mortality (95% CI)**	0.7063 (0.5937-0.8190)	0.5986 (0.4672-0.7300)	0.6448 (0.5194-0.7702)	0.7084 (0.5971-0.8197)	Method does not allow calculation of the C-statistic for lung cancer mortality
**C-for-benefit (95% CI)**	0.4801 (0.3382-0.6219)	0.4534 (0.3172-0.5895)	0.4706 (0.3321-0.6091)	0.4784 (0.3368-0.6199)	0.4919 (0.3613-0.6225)

Table S28: C-statistics and C-for-benefit for the risk- and effect-modelling approaches for histology specific mortality developed in NLST

Adenocarcinoma					
	**Risk modelling (individual risk-factors)**	**Risk modelling (LLPv3 model)**	**Risk modelling (PLCOm2012 model)**	**Effect modelling**	**Causal forests**
**C-statistic for lung cancer mortality (95% CI)**	0.6974 (0.6474-0.7473)	0.6699 (0.6191-0.7208)	0.6847 (0.6345-0.7349)	0.7056 (0.6578-0.7533)	Method does not allow calculation of the C-statistic for lung cancer mortality
**C-for-benefit (95% CI)**	0.4976 (0.4349-0.5603)	0.4803 (0.4185-0.5420)	0.4940 (0.4323-0.5556)	0.5017 (0.4392-0.5643)	0.5023 (0.4387-0.5659)
**Squamous cell carcinoma**					
	**Risk modelling (individual risk-factors)**	**Risk modelling (LLPv3 model)**	**Risk modelling (PLCOm2012 model)**	**Effect modelling**	**Causal forests**
**C-statistic for lung cancer mortality (95% CI)**	0.7384 (0.6656-0.8112)	0.6429 (0.5673-0.7184)	0.6795 (0.6038-0.7552)	0.7498 (0.6802-0.8194)	Method does not allow calculation of the C-statistic for lung cancer mortality
**C-for-benefit (95% CI)**	0.4549 (0.3556-0.5541)	0.4835 (0.3969-0.5702)	0.4725 (0.3816-0.5634)	0.4559 (0.3564-0.5554)	0.4404 (0.3424-0.5385)
**Other lung cancers**					
	**Risk modelling (individual risk-factors)**	**Risk modelling (LLPv3 model)**	**Risk modelling (PLCOm2012 model)**	**Effect modelling**	**Causal forests**
**C-statistic for lung cancer mortality (95% CI)**	0.7252 (0.6509-0.7995)	0.6822 (0.6006-0.7638)	0.6888 (0.6073-0.7703)	0.7479 (0.6782-0.8176)	Method does not allow calculation of the C-statistic for lung cancer mortality
**C-for-benefit (95% CI)**	0.4513 (0.3515-0.5510)	0.4541 (0.3586-0.5496)	0.4361 (0.3406-0.5316)	0.4522 (0.3525-0.5519)	0.4736 (0.3775-0.5698)
**Small cell carcinoma**					
	**Risk modelling (individual risk-factors)**	**Risk modelling (LLPv3 model)**	**Risk modelling (PLCOm2012 model)**	**Effect modelling**	**Causal forests**
**C-statistic for lung cancer mortality (95% CI)**	0.7445 (0.6825-0.8065)	0.6786 (0.6125-0.7447)	0.6998 (0.6346-0.7650)	0.7497 (0.6902-08091)	Method does not allow calculation of the C-statistic for lung cancer mortality
**C-for-benefit (95% CI)**	0.4797 (0.3901-0.5693)	0.4758 (0.3944-0.5571)	0.4756 (0.3916-0.5596)	0.4804 (0.3905-0.5703)	0.5501 (0.4567-0.6434)

Table S29: C-statistics and C-for-benefit for the risk- and effect-modelling approaches for histology specific mortality developed in NELSON and validated in NLST

Adenocarcinoma					
	**Risk modelling (individual risk-factors)**	**Risk modelling (LLPv3 model)**	**Risk modelling (PLCOm2012 model)**	**Effect modelling**	**Causal forests**
**C-statistic for lung cancer mortality (95% CI)**	0.6794 (0.6299-0.7289)	0.6800 (0.6310-0.7290)	0.6944 (0.6463-0.7425)	0.6785 (0.6284-0.7286)	Method does not allow external validation
**C-for-benefit (95% CI)**	0.4960 (0.4347-0.5573)	0.5179 (0.4569-0.5789)	0.4967 (0.4356-0.5579)	0.4971 (0.4360-0.5582)	Method does not allow external validation
**Squamous cell carcinoma**					
	**Risk modelling (individual risk-factors)**	**Risk modelling (LLPv3 model)**	**Risk modelling (PLCOm2012 model)**	**Effect modelling**	**Causal forests**
**C-statistic for lung cancer mortality (95% CI)**	0.6581 (0.5748-0.7414)	0.5933 (0.5105-0.6761)	0.6432 (0.5608-0.7256)	0.7015 (0.6279-0.7752)	Method does not allow external validation
**C-for-benefit (95% CI)**	0.5262 (0.4298-0.6226)	0.5165 (0.4299-0.6031)	0.5306 (0.4394-0.6218)	0.5261 (0.4297-0.6225)	Method does not allow external validation
**Other lung cancers**					
	**Risk modelling (individual risk-factors)**	**Risk modelling (LLPv3 model)**	**Risk modelling (PLCOm2012 model)**	**Effect modelling**	**Causal forests**
**C-statistic for lung cancer mortality (95% CI)**	0.7044 (0.6285-0.7803)	0.6932 (0.6149-0.7714)	0.7026 (0.6248-0.7804)	0.7019 (0.6248-0.7790)	Method does not allow external validation
**C-for-benefit (95% CI)**	0.4448 (0.3507-0.5390)	0.4541 (0.3586-0.5496)	0.4361 (0.3407-0.5316)	0.4450 (0.3517-0.5383)	Method does not allow external validation
**Small cell carcinoma**					
	**Risk modelling (individual risk-factors)**	**Risk modelling (LLPv3 model)**	**Risk modelling (PLCOm2012 model)**	**Effect modelling**	**Causal forests**
**C-statistic for lung cancer mortality (95% CI)**	0.7254 (0.6593-0.7915)	0.6783 (0.6122-0.7445)	0.7058 (0.6427-0.7689)	0.7267 (0.6612-0.7921)	Method does not allow external validation
**C-for-benefit (95% CI)**	0.4817 (0.3920-0.5714)	0.4758 (0.3944-0.5571)	0.4755 (0.3915-0.5595)	0.4817 (0.3920-0.5714)	Method does not allow external validation

Table S30: C-statistics and C-for-benefit for the risk- and effect-modelling approaches for histology specific mortality developed in NLST and validated in NELSON

Adenocarcinoma					
	**Risk modelling (individual risk-factors)**	**Risk modelling (LLPv3 model)**	**Risk modelling (PLCOm2012 model)**	**Effect modelling**	**Causal forests**
**C-statistic for lung cancer mortality (95% CI)**	0.6695 (0.5894-0.7497)	0.6283 (0.5423-0.7144)	0.6709 (0.5914-0.7504)	0.7003 (0.6270-0.7737)	Method does not allow external validation
**C-for-benefit (95% CI)**	0.4890 (0.3943-0.5837)	0.5175 (04188-0.6162)	0.4958 (0.4000-0.5915)	0.4902 (0.3951-0.5852)	Method does not allow external validation
**Squamous cell carcinoma**					
	**Risk modelling (individual risk-factors)**	**Risk modelling (LLPv3 model)**	**Risk modelling (PLCOm2012 model)**	**Effect modelling**	**Causal forests**
**C-statistic for lung cancer mortality (95% CI)**	0.6499 (0.5389-0.7609)	0.5777 (0.4541-0.7014)	0.6128 (0.4951-0.7305)	0.6824 (0.5777-0.7870)	Method does not allow external validation
**C-for-benefit (95% CI)**	0.5965 (0.4681)	0.5596 (0.4341-0.6852)	0.5867 (0.4548-0.7187)	0.5956 (0.4671-0.7242)	Method does not allow external validation
**Other lung cancers**					
	**Risk modelling (individual risk-factors)**	**Risk modelling (LLPv3 model)**	**Risk modelling (PLCOm2012 model)**	**Effect modelling**	**Causal forests**
**C-statistic for lung cancer mortality (95% CI)**	0.6439 (0.4742-0.8137)	0.6357 (0.4479-0.8235)	0.6522 (0.4814-0.8230)	0.6652 (0.4948-0.8355)	Method does not allow external validation
**C-for-benefit (95% CI)**	0.4855 (0.2888-0.6821)	0.4299 (0.2349-0.6249)	0.4796 (0.2827-0.6765)	0.4860 (0.2876-0.6843)	Method does not allow external validation
**Small cell carcinoma**					
	**Risk modelling (individual risk-factors)**	**Risk modelling (LLPv3 model)**	**Risk modelling (PLCOm2012 model)**	**Effect modelling**	**Causal forests**
**C-statistic for lung cancer mortality (95% CI)**	0.6785 (0.5542-0.8027)	0.5990 (0.4677-0.7302)	0.6406 (0.5136-0.767)	0.6847 (0.5654-0.8040)	Method does not allow external validation

Figure S17: Calibration for relative benefit (overall LCM) for risk- and effect-models developed in NELSON

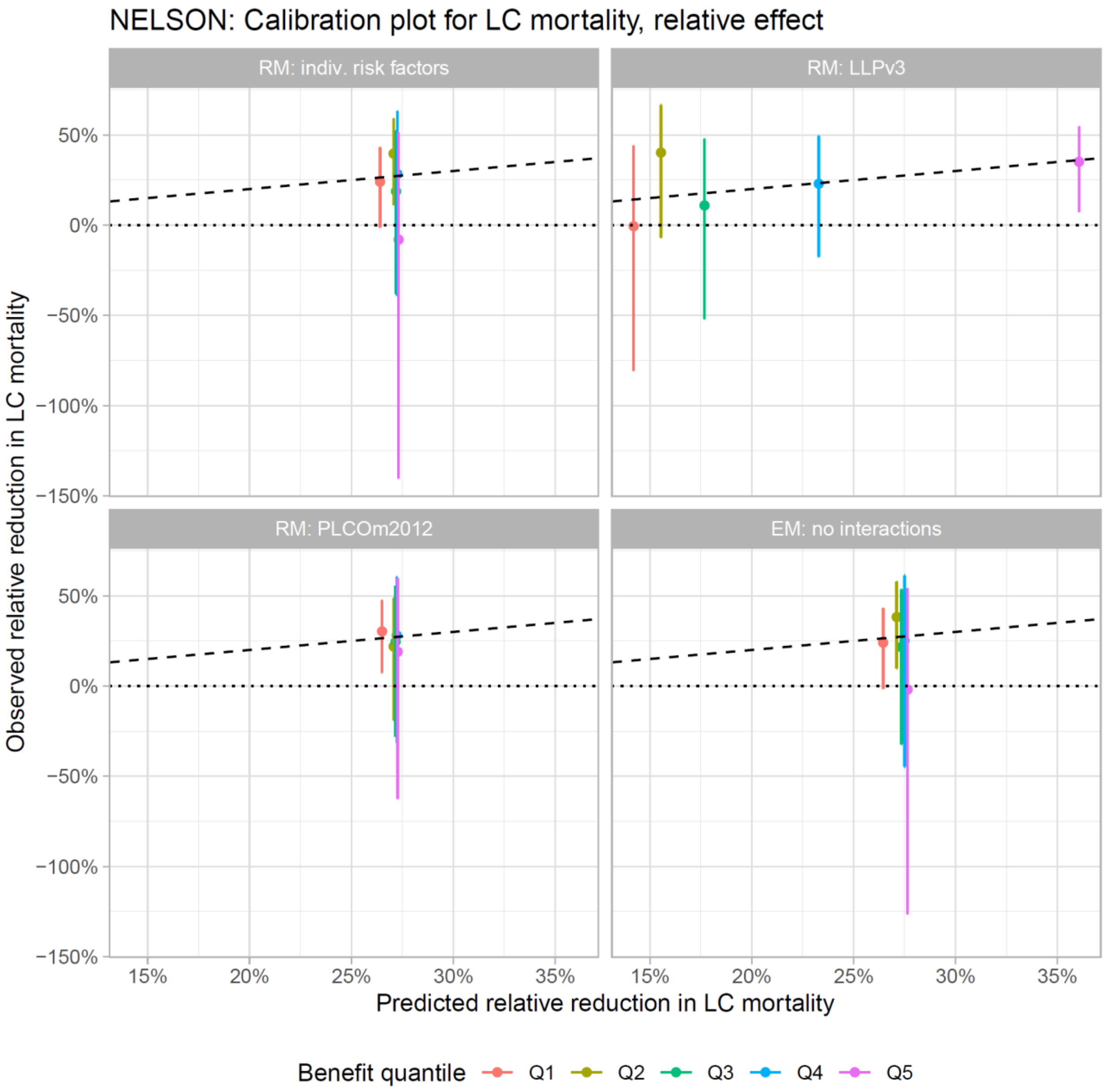

Figure notes: Based on N = 400 lung cancer deaths in NELSON. The error bars represent the 95% confidence intervals.

**Quintile thresholds**	**Q1**	**Q2**	**Q3**	**Q4**	**Q5**
**Risk modeling (individual risk-factors)**	<27.00%	27.00% - 27.10%	27.10% - 27.20%	27.20% - 27.30%	≥27.30%
**Risk modeling (LLPv3 model)**	<14.90%	14.90% - 16.30%	16.30% - 19.50%	19.50% - 28.20%	≥28.20%
**Risk modeling (PLCOm2012 model)**	<26.99%	26.99% - 27.13%	27.13% - 27.20%	27.20% - 27.25%	≥27.25%
**Effect modeling**	<26.90%	26.90% - 27.20%	27.20% - 27.40%	27.40% - 27.60%	≥27.60%

Figure S18: Calibration for relative benefit (overall LCM) for risk- and effect- models developed in NLST

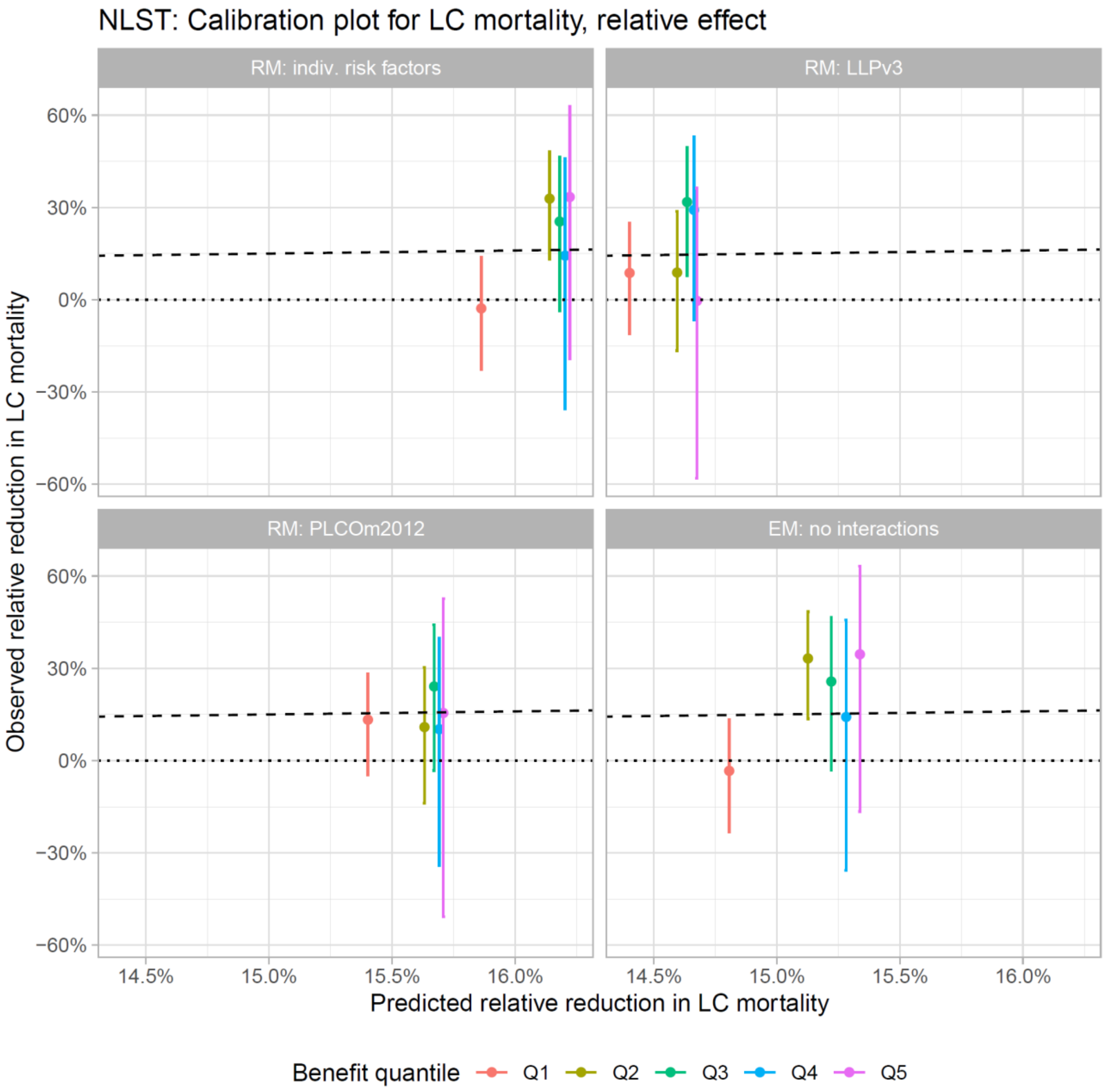

Figure notes: Based on N = 977 lung cancer deaths in NLST. The error bars represent the 95% confidence intervals.

**Quintile thresholds**	**Q1**	**Q2**	**Q3**	**Q4**	**Q5**
**Risk modeling (individual risk-factors)**	<16.10%	16.10% - 16.17%	16.17% - 16.19%	16.19% - 16.21%	≥16.21%
**Risk modeling (LLPv3 model)**	<14.56%	14.56% - 14.62%	14.62% - 14.65%	14.65% - 14.67%	≥14.67%
**Risk modeling (PLCOm2012 model)**	<15.60%	15.60% - 15.66%	15.66% - 15.68%	15.68% - 15.70%	≥15.70%
**Effect modeling**	<15.05%	15.05% - 15.18%	15.18% - 15.25%	15.25% - 15.31%	≥15.31%

Figure S19: Calibration for relative benefit (overall LCM) for causal forests developed in NELSON and NLST

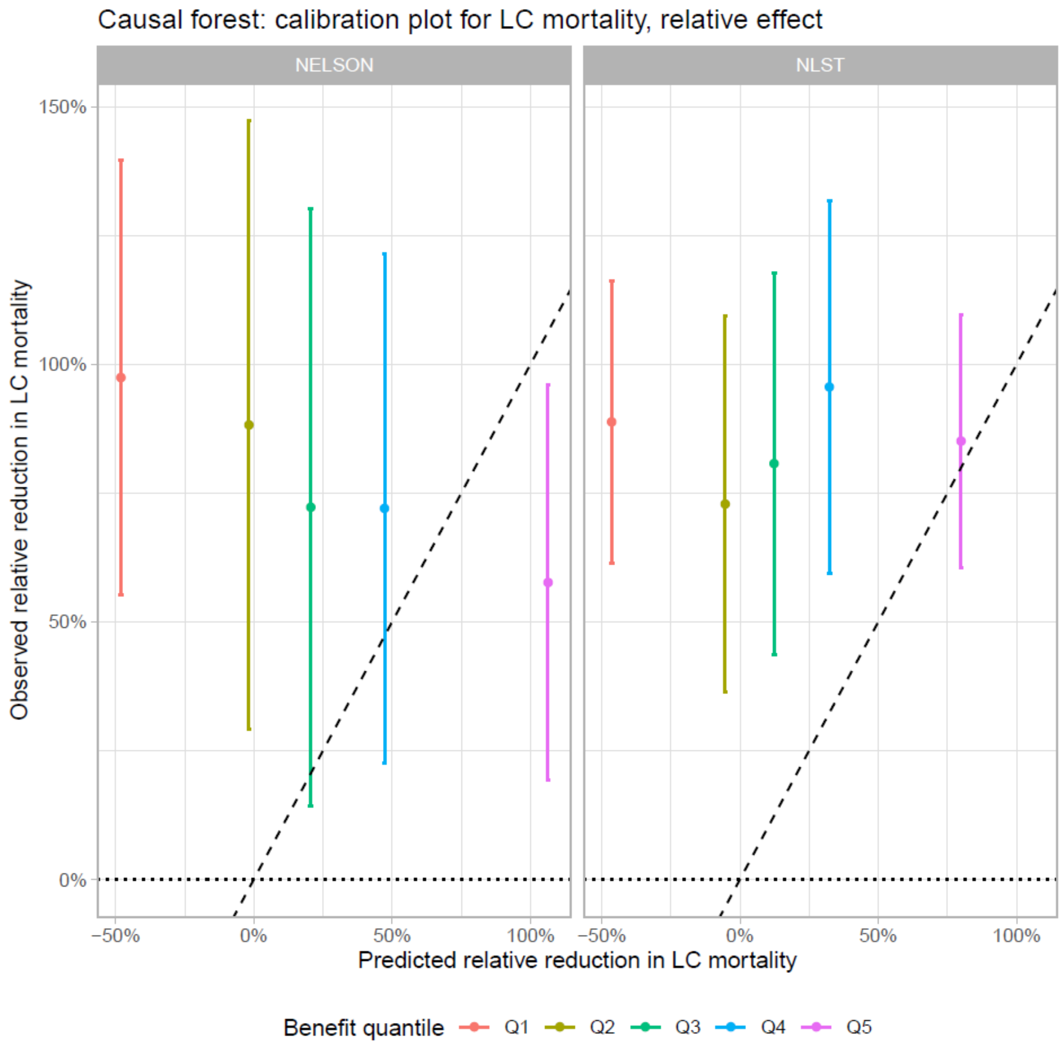

Figure notes: Based on N = 400 lung cancer deaths in NELSON and N = 977 lung cancer deaths in NLST. The error bars represent the 95% confidence intervals.

Quintile thresholds	Q1	Q2	Q3	Q4	Q5
NELSON	< -14.6%	-14.6%-9.71%	9.71%-32.2%	32.25-65.0%	>65.0%
NLST	< -15.4%	-15.4%-4.11%	4.11%-20.9%	20.9%-45.0%	>45.0%

Figure S20: Calibration for relative benefit (adenocarcinoma-specific mortality) for risk- and effect- models developed in NELSON

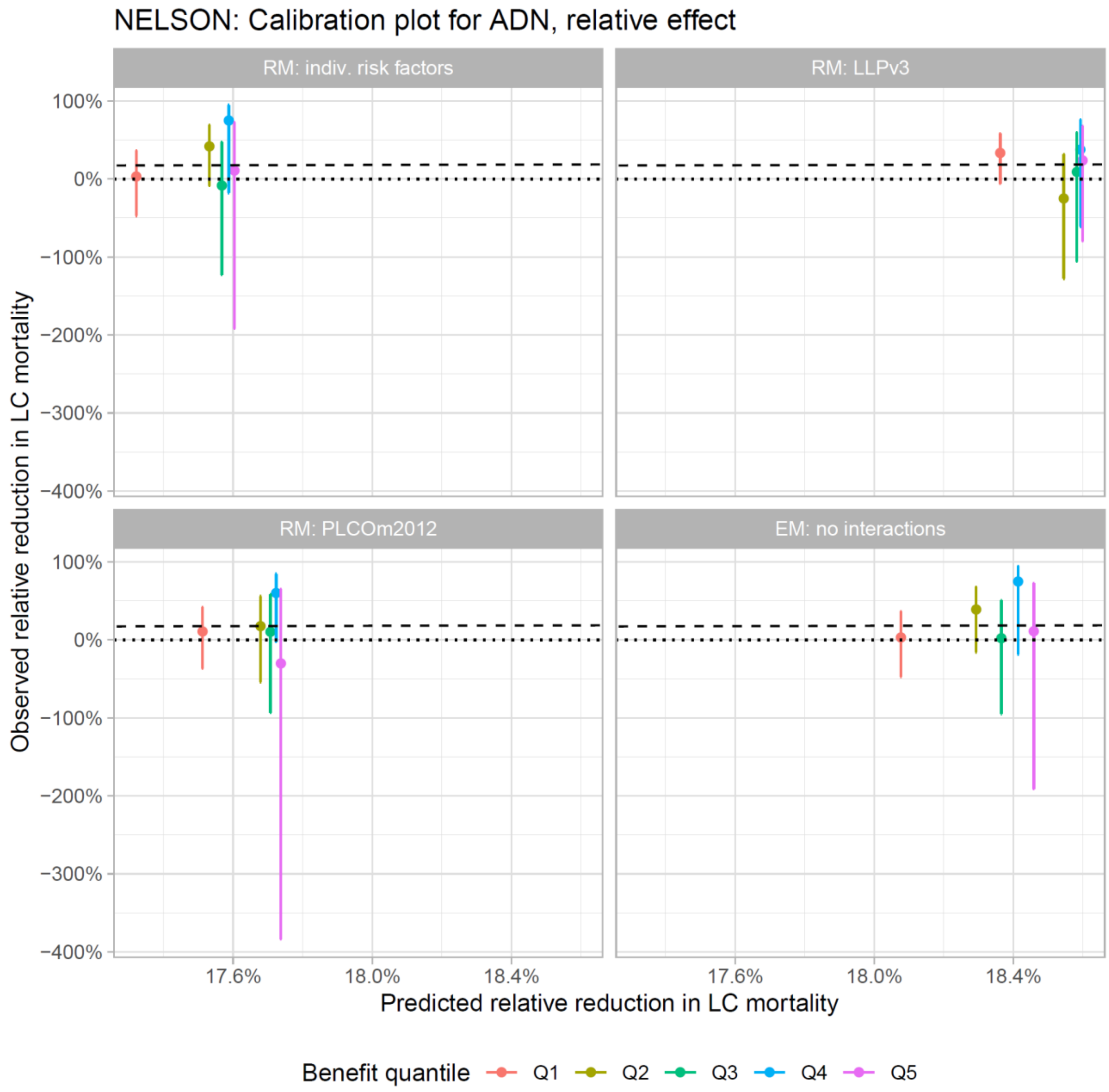

Figure notes: Based on N = 178 Adenocarcinoma deaths in NELSON. The error bars represent the 95% confidence intervals.

Quintile thresholds	Q1	Q2	Q3	Q4	Q5
**Risk modeling (individual risk-factors)**	<17.50%	17.50% - 17.55%	17.55% - 17.58%	17.58% - 17.60%	≥17.60%
**Risk modeling (LLPv3 model)**	<18.51%	18.51% - 18.57%	18.57% - 18.59%	18.59% - 18.60%	≥18.60%
**Risk modeling (PLCOm2012 model)**	<17.66%	17.66% - 17.70%	17.70% - 17.72%	17.72% - 17.73%	≥17.73%
**Effect modeling**	<18.24%	18.24% - 18.34%	18.34% - 18.39%	18.39% - 18.44%	≥18.44%

Figure S21: Calibration for relative benefit (adenocarcinoma-specific mortality) for risk- and effect- models developed in NLST

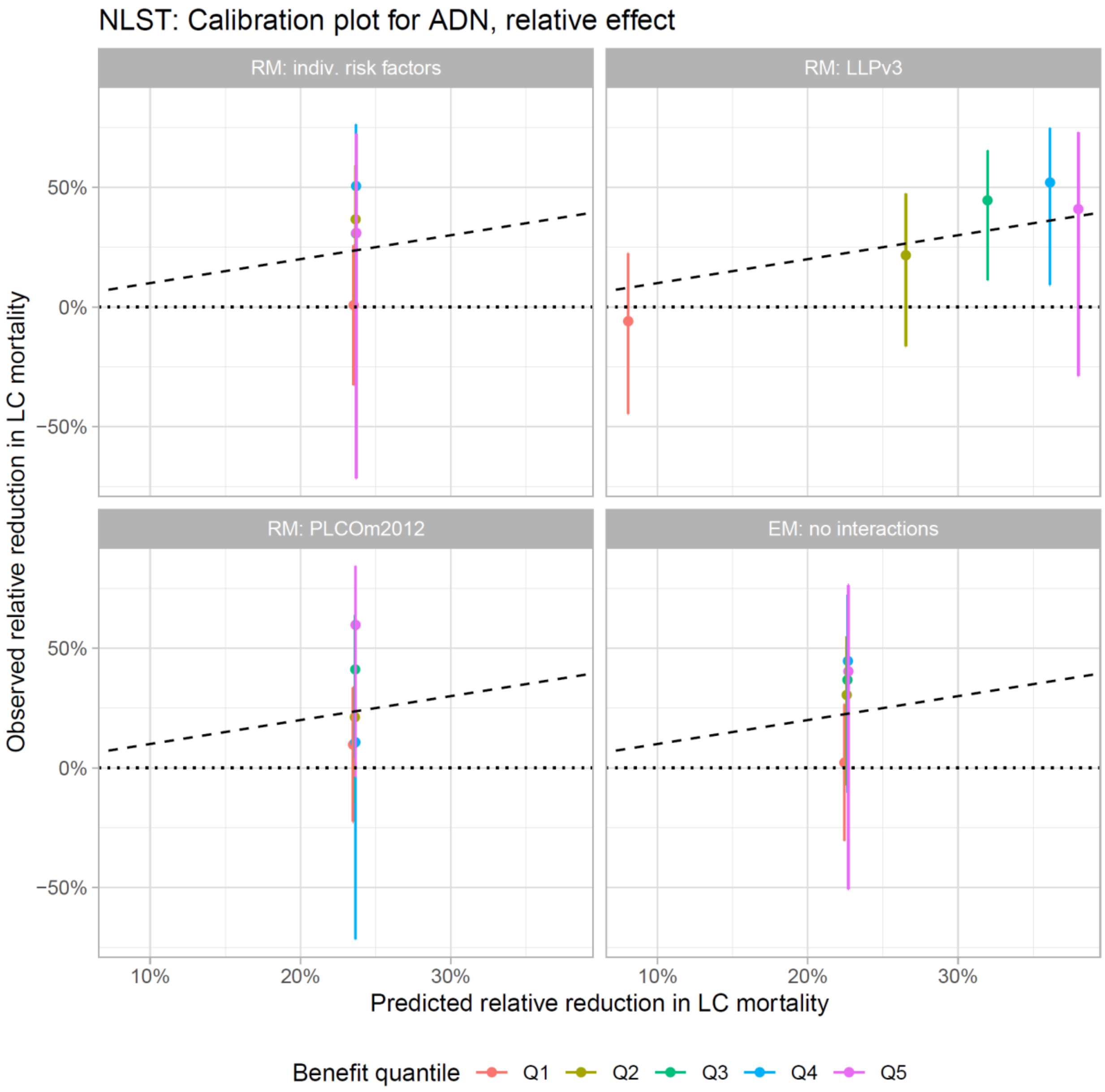

Figure notes: Based on N = 393 Adenocarcinoma deaths in NLST. The error bars represent the 95% confidence intervals.

**Quintile thresholds**	**Q1**	**Q2**	**Q3**	**Q4**	**Q5**
**Risk modeling (individual risk-factors)**	<23.66%	23.66% - 23.69%	23.69% - 23.70%	23.70% - 23.71%	≥23.71%
**Risk modeling (LLPv3 model)**	<22.50%	22.50% - 30.10%	30.10% - 34.40%	34.40% - 37.30%	≥37.30%
**Risk modeling (PLCOm2012 model)**	<23.61%	23.61% - 23.64%	23.64% - 23.66%	23.66% - 23.67%	≥23.67%
**Effect modeling**	<22.56%	22.56% - 22.62%	22.62% - 22.66%	22.66% - 22.69%	≥22.69%

Figure S22: Calibration for relative benefit (squamous cell carcinoma-specific mortality) for risk- and effect- models developed in NELSON

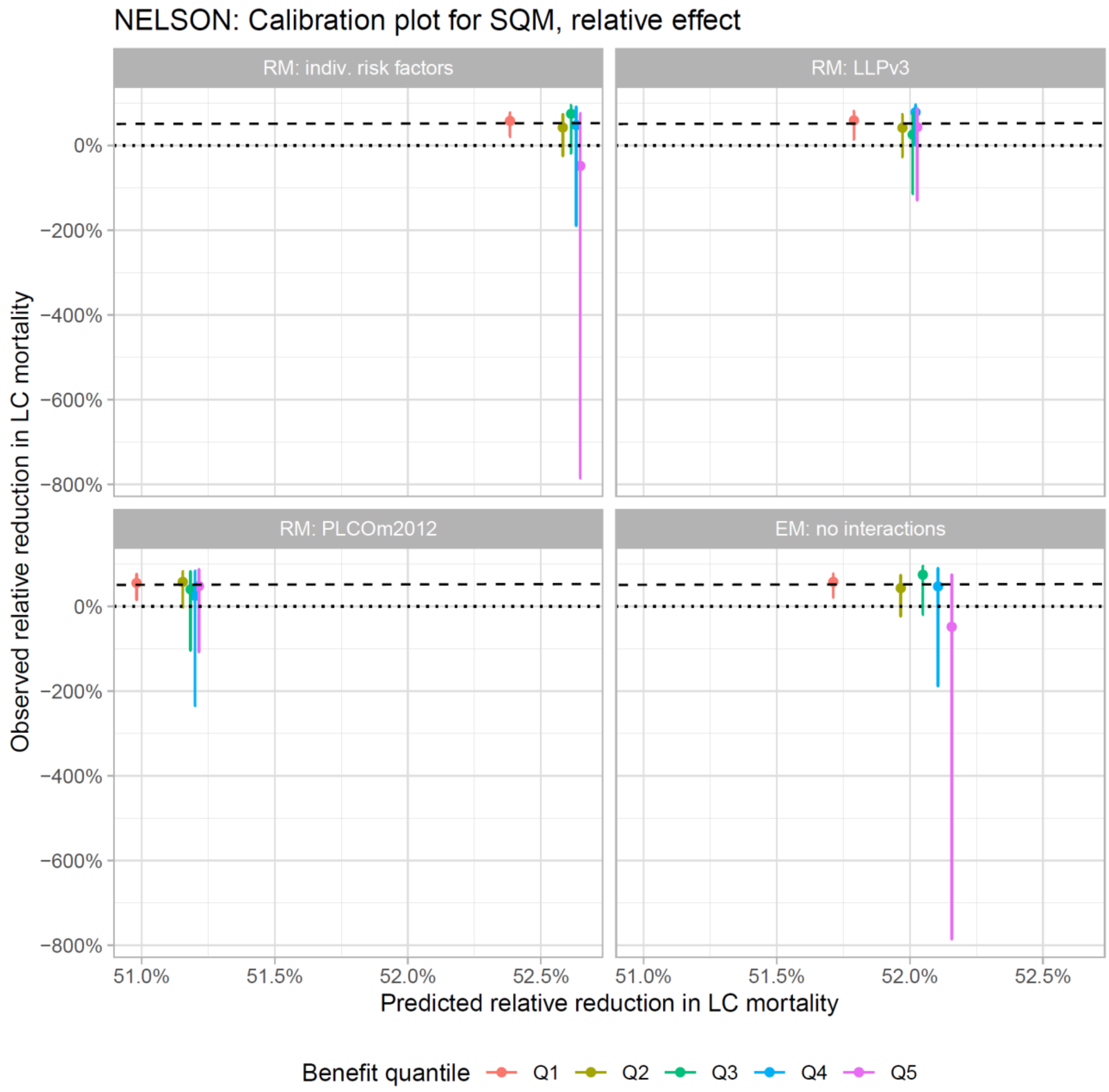

Figure notes: Based on N = 94 Squamous-cell carcinoma deaths in NELSON. The error bars represent the 95% confidence intervals.

**Quintile thresholds**	**Q1**	**Q2**	**Q3**	**Q4**	**Q5**
**Risk modeling (individual risk-factors)**	<52.56%	52.56% - 52.60%	52.60% - 52.62%	52.62% - 52.64%	≥52.64%
**Risk modeling (LLPv3 model)**	<51.94%	51.94% - 52.00%	52.00% - 52.02%	52.02% - 52.03%	≥52.03%
**Risk modeling (PLCOm2012 model)**	<51.13%	51.13% - 51.17%	51.17% - 51.19%	51.19% - 51.21%	≥51.21%
**Effect modeling**	<51.90%	51.90% - 52.02%	52.02% - 52.08%	52.08% - 52.13%	≥52.13%

Figure S23: Calibration for relative benefit (squamous cell carcinoma-specific mortality) for risk- and effect- models developed in NLST

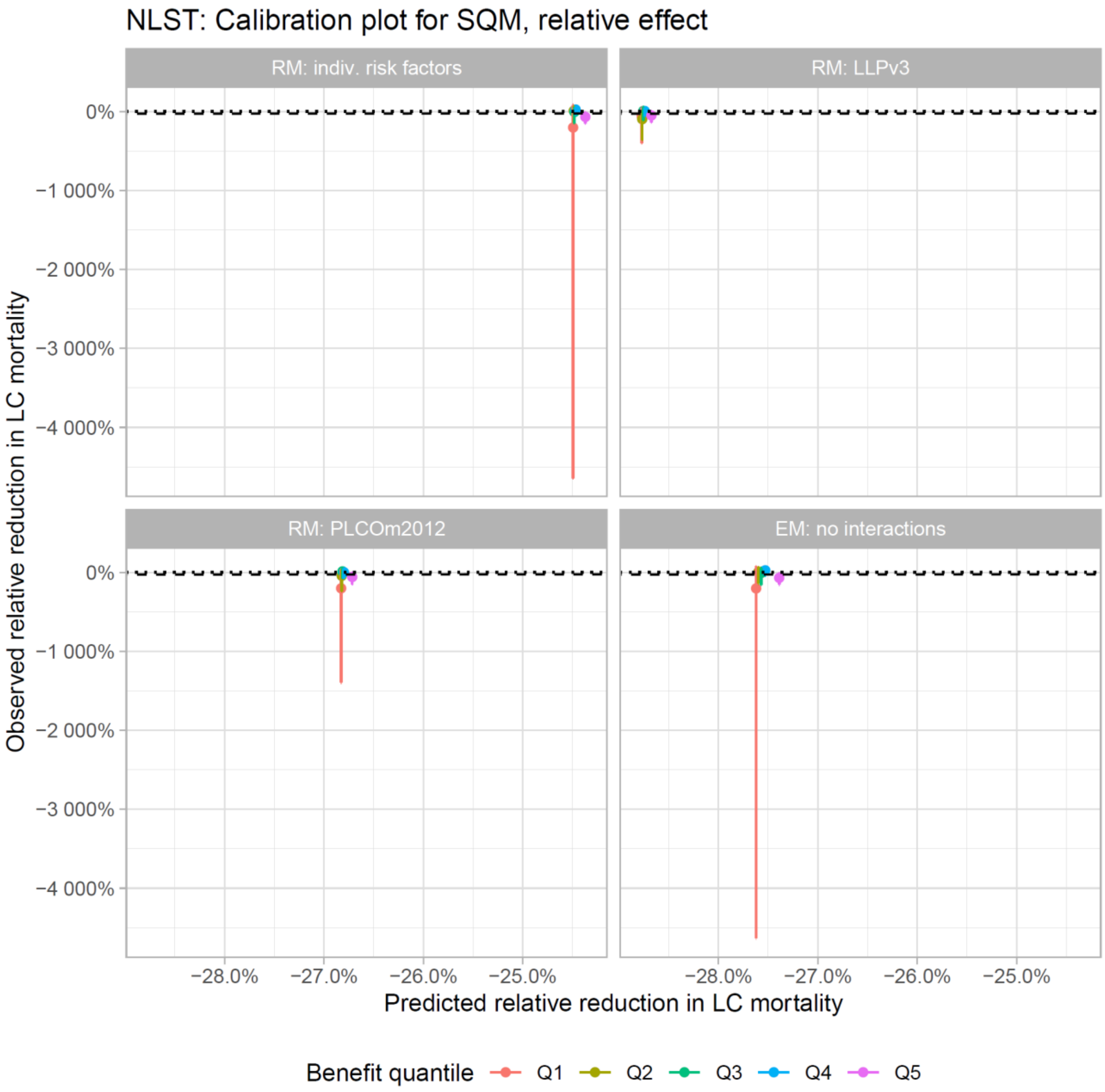

Figure notes: Based on N = 461 Squamous-cell carcinoma deaths in NLST. The error bars represent the 95% confidence intervals.

**Quintile thresholds**	**Q1**	**Q2**	**Q3**	**Q4**	**Q5**
**Risk modeling (individual risk-factors)**	<-24.49%	-24.49% - -24.48%	-24.48% - -24.47%	-24.47% - -24.46%	≥-24.46%
**Risk modeling (LLPv3 model)**	<-28.77%	-28.77% - -28.76%	-28.76% - -28.75%	-28.75% - -28.73%	≥-28.73%
**Risk modeling (PLCOm2012 model)**	<-26.82%	-26.82% - -26.82%	-26.82% - -26.81%	-26.81% - -26.79%	≥-26.79%
**Effect modeling**	<-27.61%	-27.61% - -27.58%	-27.58% - -27.55%	-27.55% - -27.50%	≥-27.50%

Figure S24: Calibration for relative benefit (Others and non-small cell carcinoma not otherwise specified -specific mortality) for risk- and effect-models developed in NELSON

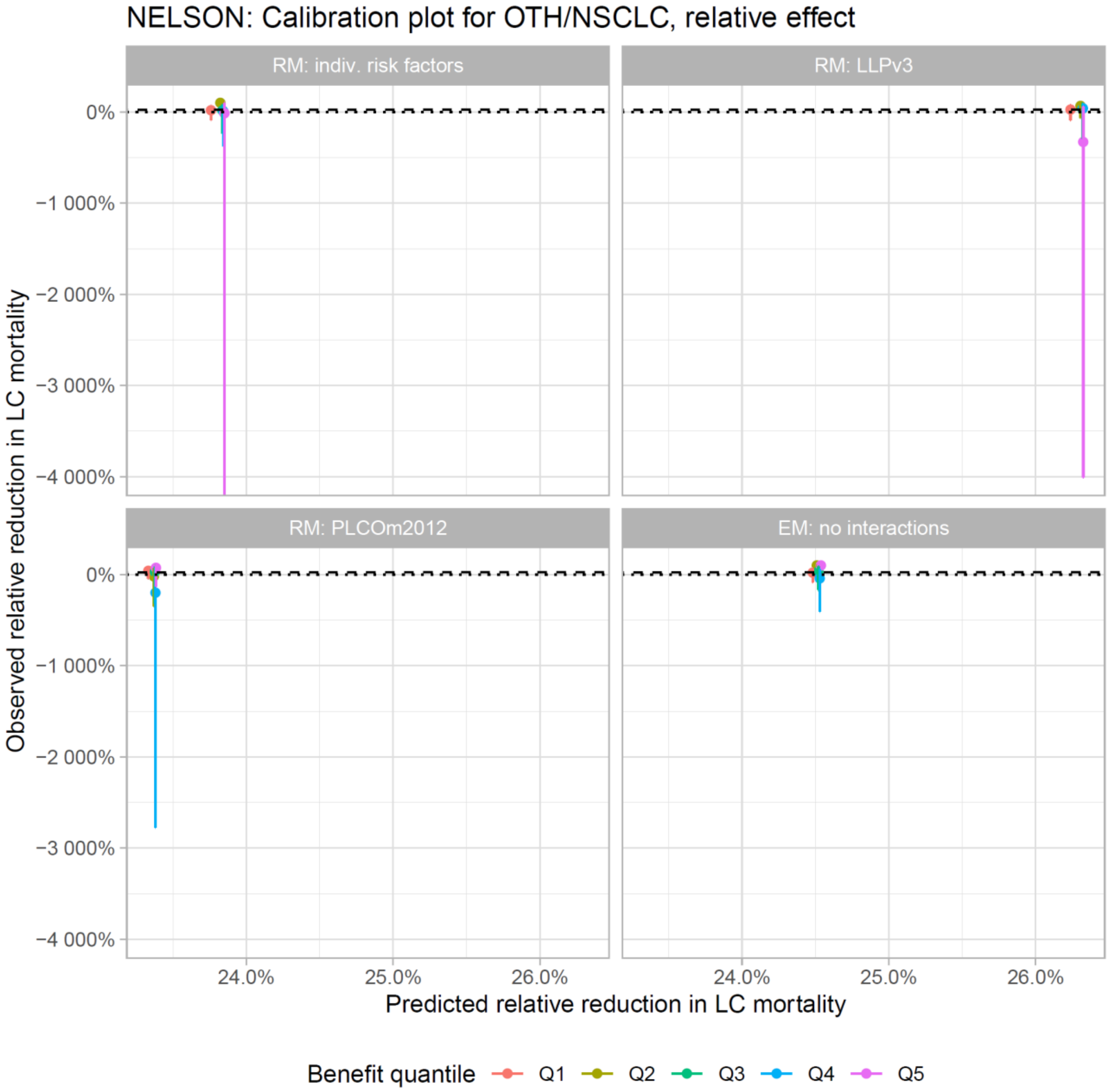

Figure notes: Based on N = 43 Other lung cancer deaths in NELSON. The error bars represent the 95% confidence intervals.

**Quintile thresholds**	**Q1**	**Q2**	**Q3**	**Q4**	**Q5**
**Risk modeling (individual risk-factors)**	<23.81%	23.81% - 23.83%	23.83% - 23.84%	23.84% - 23.85%	≥23.85%
**Risk modeling (LLPv3 model)**	<26.29%	26.29% - 26.31%	26.31% - 26.32%	26.32% - 26.32%	≥26.32%
**Risk modeling (PLCOm2012 model)**	<23.36%	23.36% - 23.37%	23.37% - 23.38%	23.38% - 23.38%	≥23.38%
**Effect modeling**	<24.50%	24.50% - 24.52%	24.52% - 24.53%	24.53% - 24.53%	≥24.53%

Figure S25: Calibration for relative benefit (Others and non-small cell carcinoma not otherwise specified -specific mortality) for risk- and effect- models developed in NLST

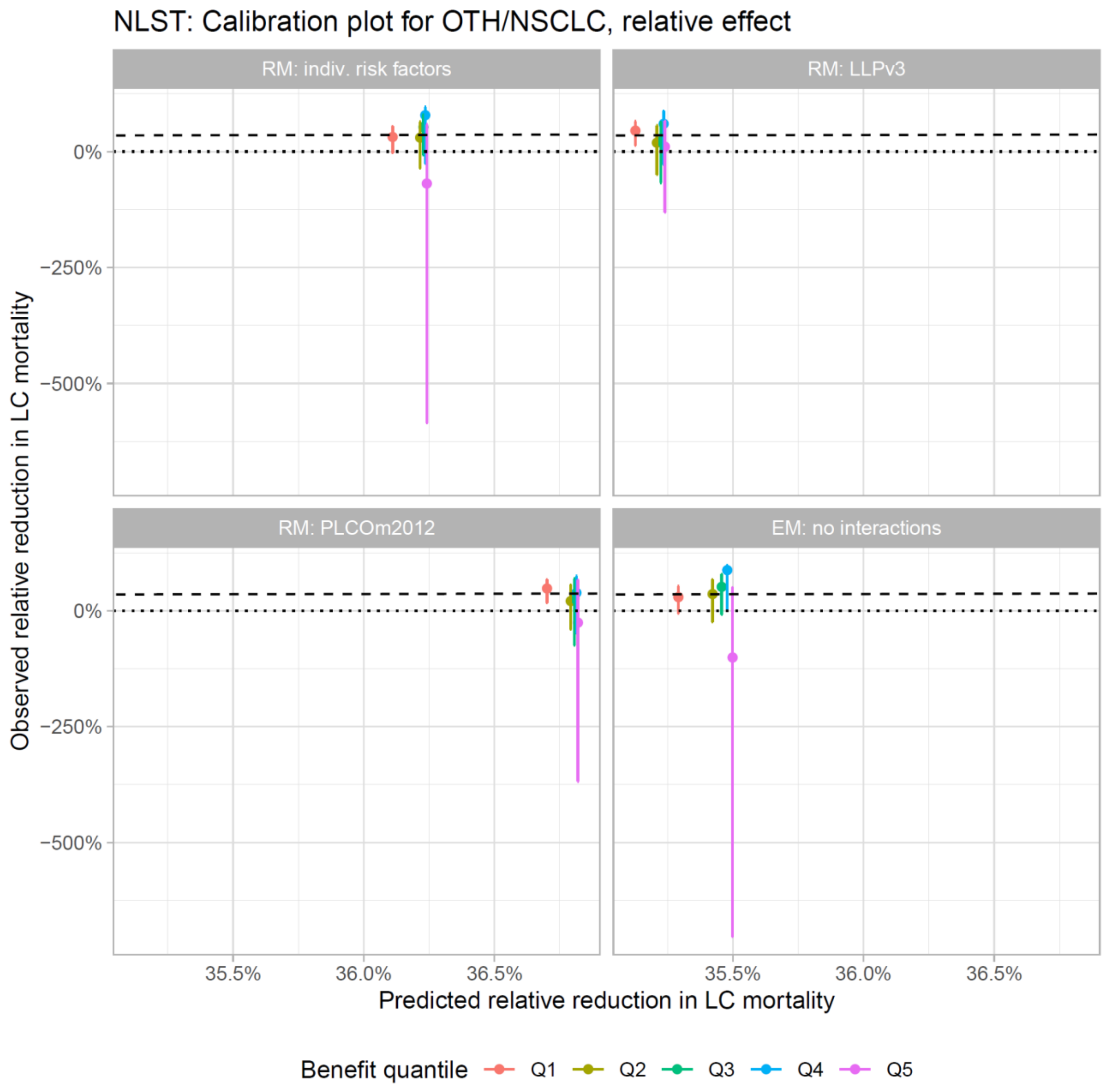

Figure notes: Based on N = 176 Other lung cancer deaths in NLST. The error bars represent the 95% confidence intervals.

**Quintile thresholds**	**Q1**	**Q2**	**Q3**	**Q4**	**Q5**
**Risk modeling (individual risk-factors)**	<36.20%	36.20% - 36.22%	36.22% - 36.23%	36.23% - 36.24%	≥36.24%
**Risk modeling (LLPv3 model)**	<35.20%	35.20% - 35.22%	35.22% - 35.23%	35.23% - 35.24%	≥35.24%
**Risk modeling (PLCOm2012 model)**	<36.78%	36.78% - 36.80%	36.80% - 36.81%	36.81% - 36.82%	≥36.82%
**Effect modeling**	<35.39%	35.39% - 35.44%	35.44% - 35.47%	35.47% - 35.49%	≥35.49%

Figure S26: Calibration for relative benefit (Small cell carcinoma- specific mortality) for risk- and effect- models developed in NELSON

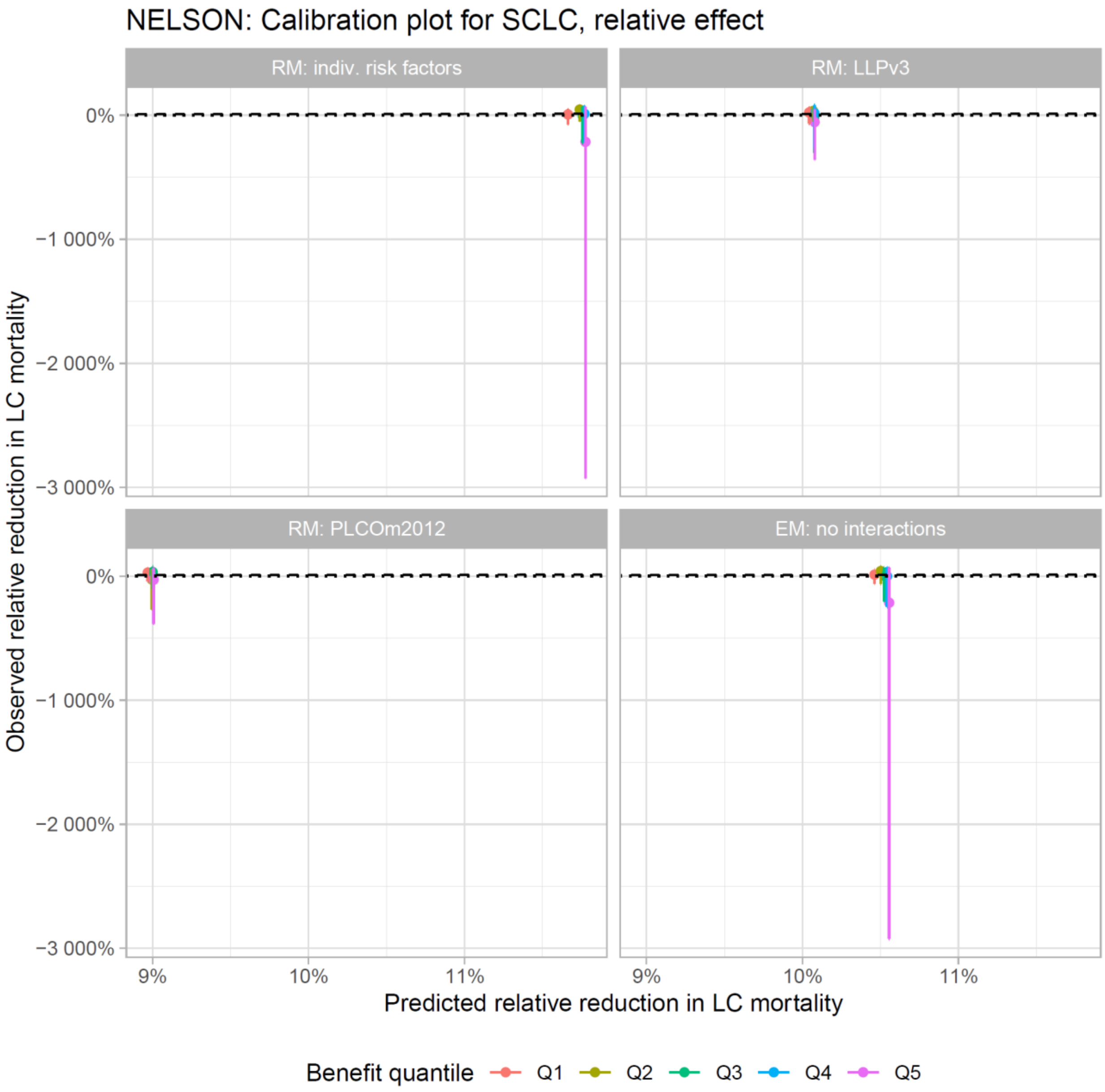

Figure notes: Based on N = 84 Small-cell carcinoma deaths in NELSON. The error bars represent the 95% confidence intervals.

**Quintile thresholds**	**Q1**	**Q2**	**Q3**	**Q4**	**Q5**
**Risk modeling (individual risk-factors)**	<11.72%	11.72% - 11.75%	11.75% - 11.76%	11.76% - 11.77%	≥11.77%
**Risk modeling (LLPv3 model)**	<10.06%	10.06% - 10.07%	10.07% - 10.08%	10.08% - 10.08%	≥10.08%
**Risk modeling (PLCOm2012 model)**	<8.99%	8.99% - 9.00%	9.00% - 9.00%	9.00% - 9.01%	≥9.01%
**Effect modeling**	<10.49%	10.49% - 10.51%	10.51% - 10.53%	10.53% - 10.55%	≥10.55%

Figure S27: Calibration for relative benefit (Small cell carcinoma- specific mortality) for risk- and effect- models developed in NLST

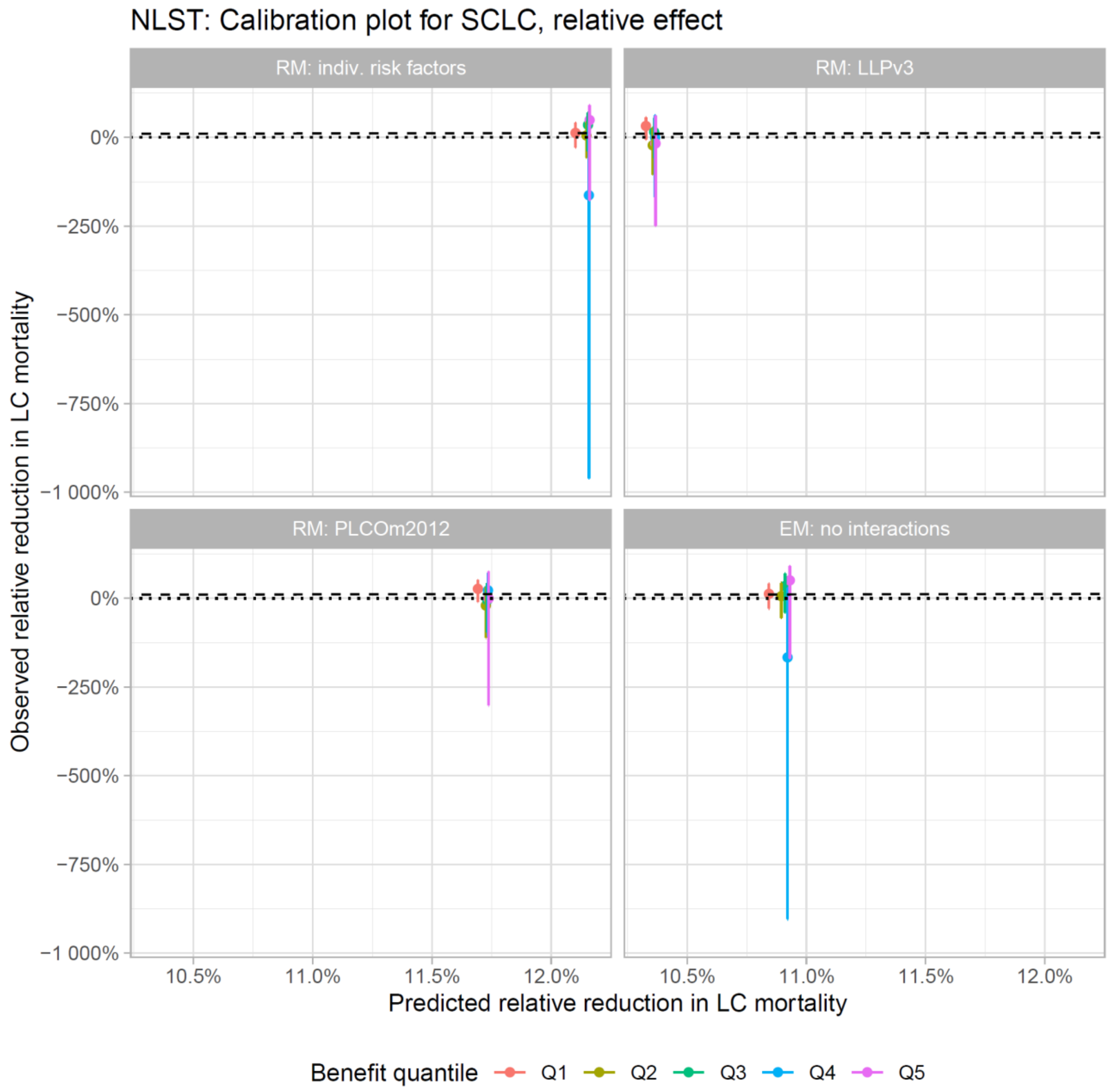

Figure notes: Based on N = 287 Small-cell carcinoma deaths in NLST. The error bars represent the 95% confidence intervals.

**Quintile thresholds**	**Q1**	**Q2**	**Q3**	**Q4**	**Q5**
**Risk modeling (individual risk-factors)**	<12.14%	12.14% - 12.15%	12.15% - 12.16%	12.16% - 12.16%	≥12.16%
**Risk modeling (LLPv3 model)**	<10.35%	10.35% - 10.36%	10.36% - 10.36%	10.36% - 10.37%	≥10.37%
**Risk modeling (PLCOm2012 model)**	<11.72%	11.72% - 11.73%	11.73% - 11.73%	11.73% - 11.74%	≥11.74%
**Effect modeling**	<10.88%	10.88% - 10.90%	10.90% - 10.92%	10.92% - 10.93%	≥10.93%

Figure S28: Calibration for relative benefit (adenocarcinoma-specific mortality) for causal forests developed in NELSON and NLST

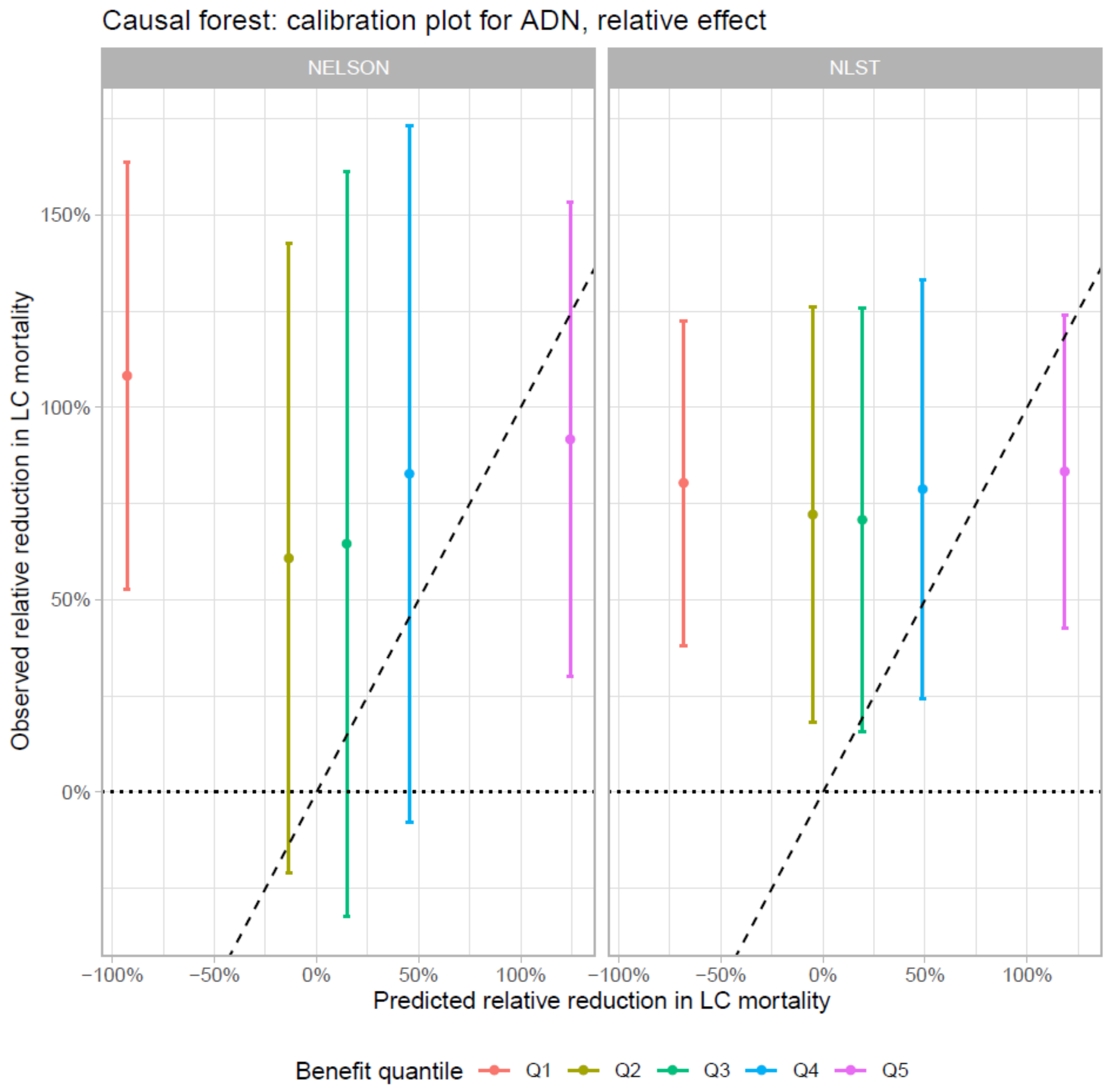

Figure notes: Based on N = 178 Adenocarcinoma deaths in NELSON and N = 393 Adenocarcinoma deaths in NLST. The error bars represent the 95% confidence intervals.

Quintile thresholds	Q1	Q2	Q3	Q4	Q5
NELSON	<-32.8%	-32.8%-2.2%	2.25%-28.1%	28.1%-65.9%	>65.9%
NLST	-19.6%	-19.6%-7.73%	7.73%-31.7%	31.7%-67.9%	>67.9%

Figure S29: Calibration for relative benefit (squamous cell-specific mortality) for causal forests developed in NELSON and NLST

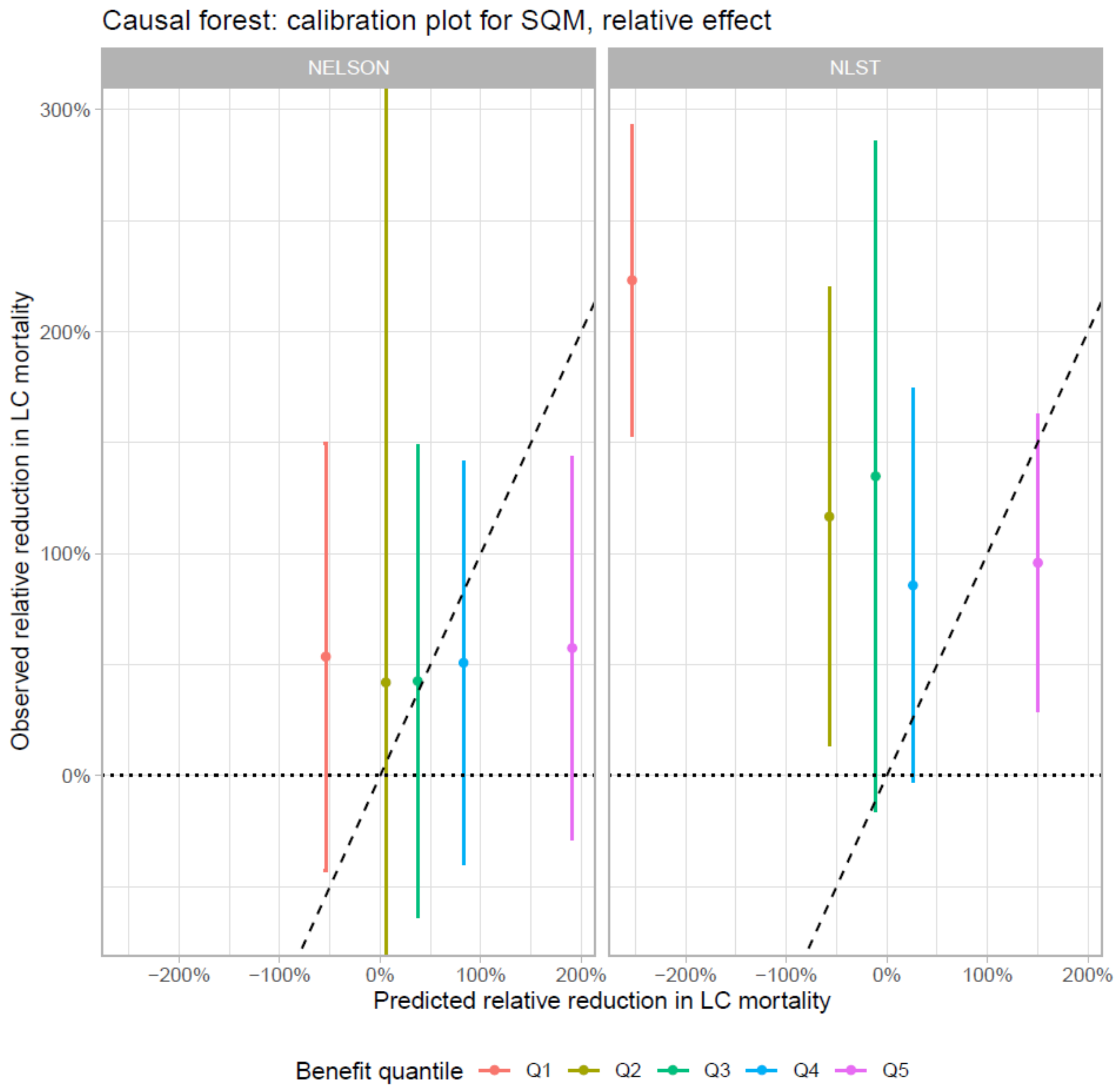

Figure notes: Based on N = 94 Squamous-cell carcinoma deaths in NELSON and N = 184 Squamous-cell carcinoma deaths in NLST. The error bars represent the 95% confidence intervals.

Quintile thresholds	Q1	Q2	Q3	Q4	Q5
NELSON	<-7.32%	-7.32%-19.5%	19.5%-58.1%	58.1%-113.0%	>113.0%
NLST	-95.2%	-95.2% - -27.2%	-27.2% - 3.77%	3.77%-52.4%	>52.4%

Figure S30: Calibration for relative benefit (Others and non-small cell carcinoma not otherwise specified -specific mortality) for causal forests developed in NELSON and NLST

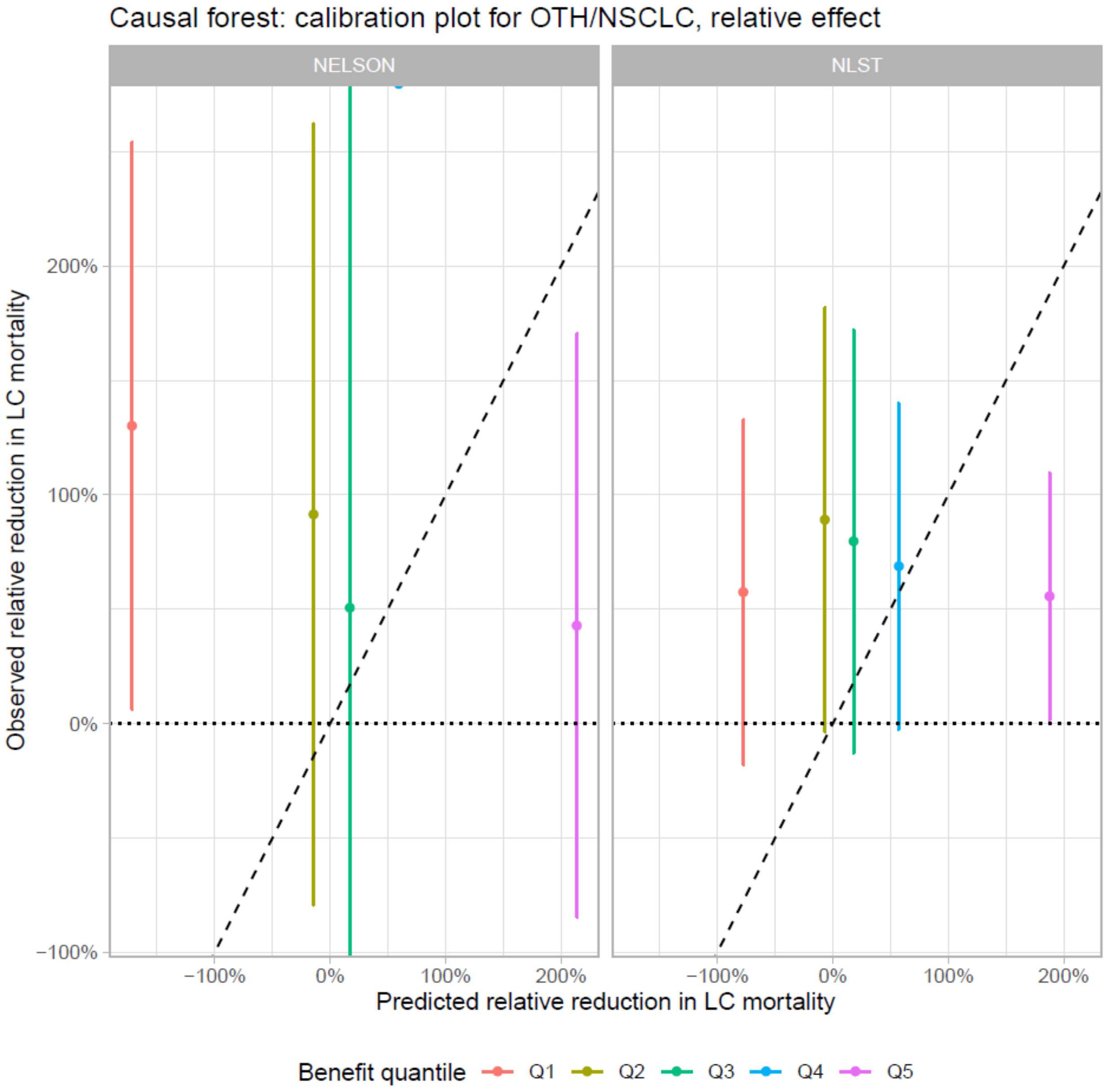

Figure notes: Based on N = 43 Other lung cancer deaths in NELSON and N = 176 Other lung cancer deaths in NLST. The error bars represent the 95% confidence intervals.

Quintile thresholds	Q1	Q2	Q3	Q4	Q5
NELSON	< -42.0%	-42.0%-4.01%	4.01%-33.2%	33.2%-92.4%	>92.4%
NLST	< -19.6%	-19.6%-5.36%	5.36%-32.7%	32.7%-87.5%	>87.5%

Figure S31: Calibration for relative benefit (small cell-specific mortality) for causal forests developed in NELSON and NLST

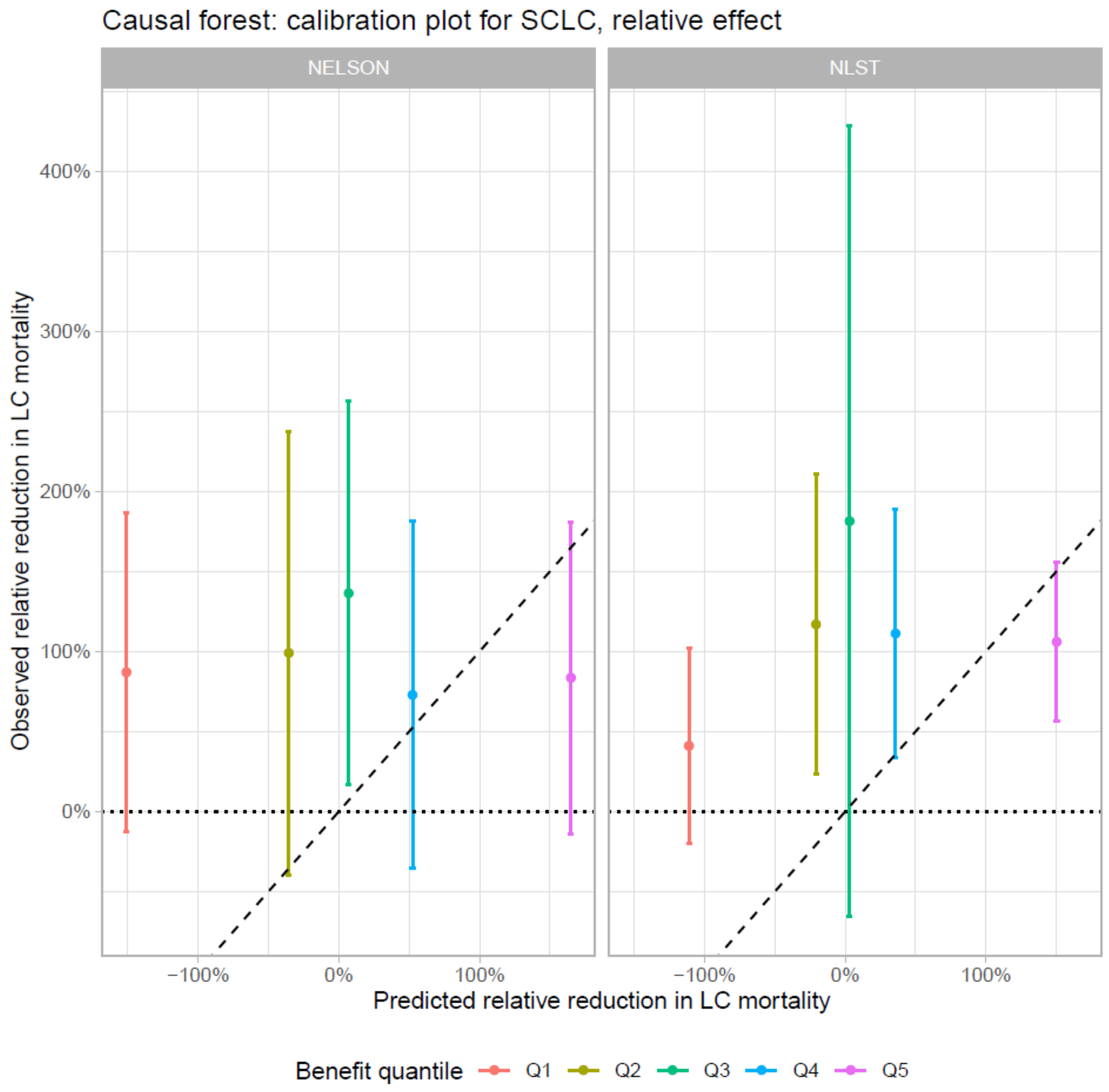

Figure notes: Based on N = 84 Small-cell carcinoma deaths in NELSON and N = 209 Small-cell carcinoma deaths in NLST. The error bars represent the 95% confidence intervals.

Quintile thresholds	Q1	Q2	Q3	Q4	Q5
NELSON	< -63.7%	-63.7%-26.4%	-11.3%-26.4%	26.4%-83.2%	>83.2%
NLST	< -39.2%	-39.2% - -5.03%	-5.03%-13.1%	13.1%-64.1%	>64.1%

Figure S32: Lung cancer deaths prevented by risk-prediction model quintiles based on NELSON in NELSON

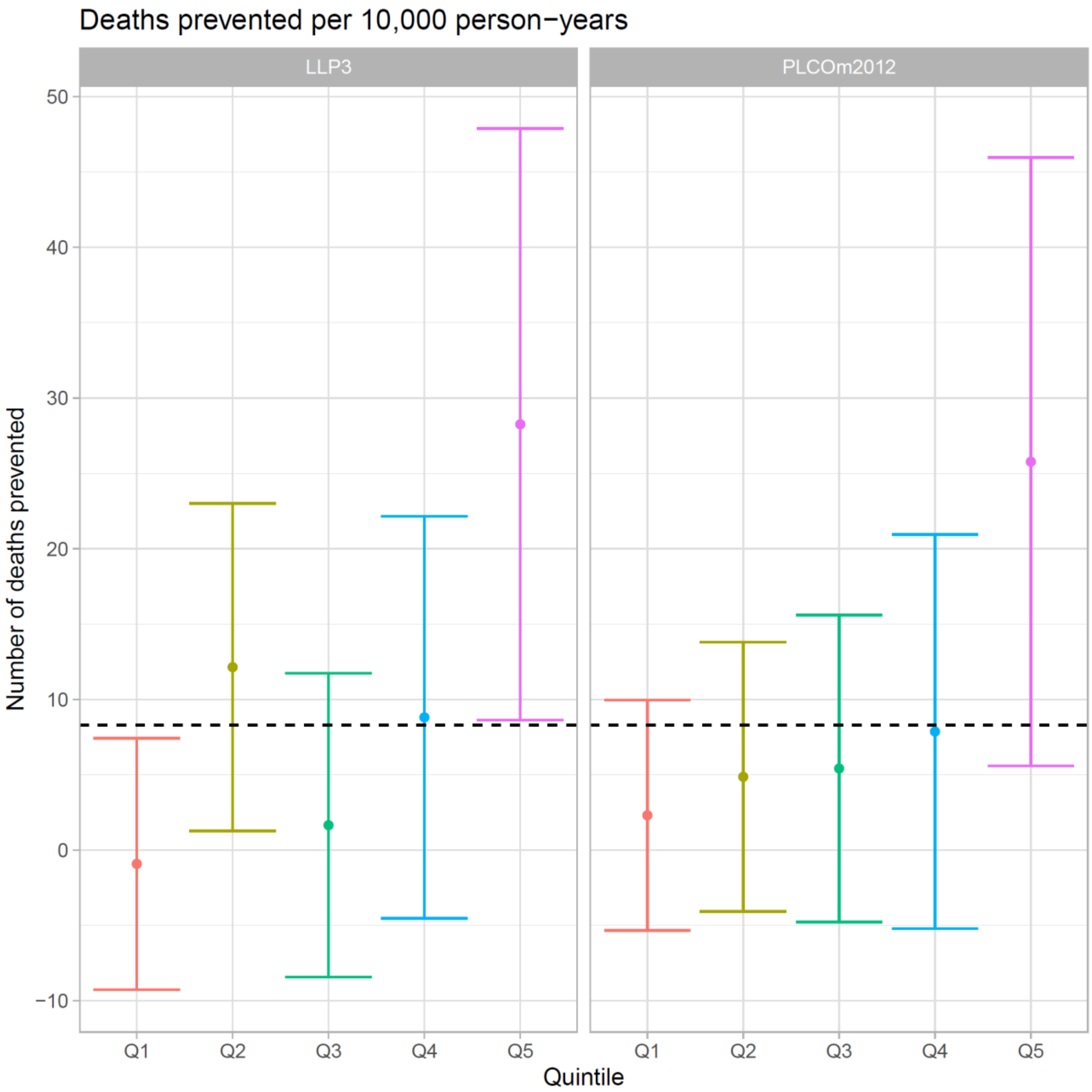

Figure notes: Based on N = 400 lung cancer deaths in NELSON. The error bars represent the 95% confidence intervals.

Quintile thresholds	Q1	Q2	Q3	Q4	Q5
Risk modelling (LLPv3 model)	<0.27%	0.27% - 0.41%	0.41% - 0.75%	0.75% - 1.73%	>1.73%
Risk modelling (PLCOm2012 model)	<0.96%	0.96% - 1.42%	1.42% - 2.01%	2.01% - 3.02%	>3.02%

Figure S33: Lung cancer deaths prevented by risk-prediction model quintile based on NLST in NLST

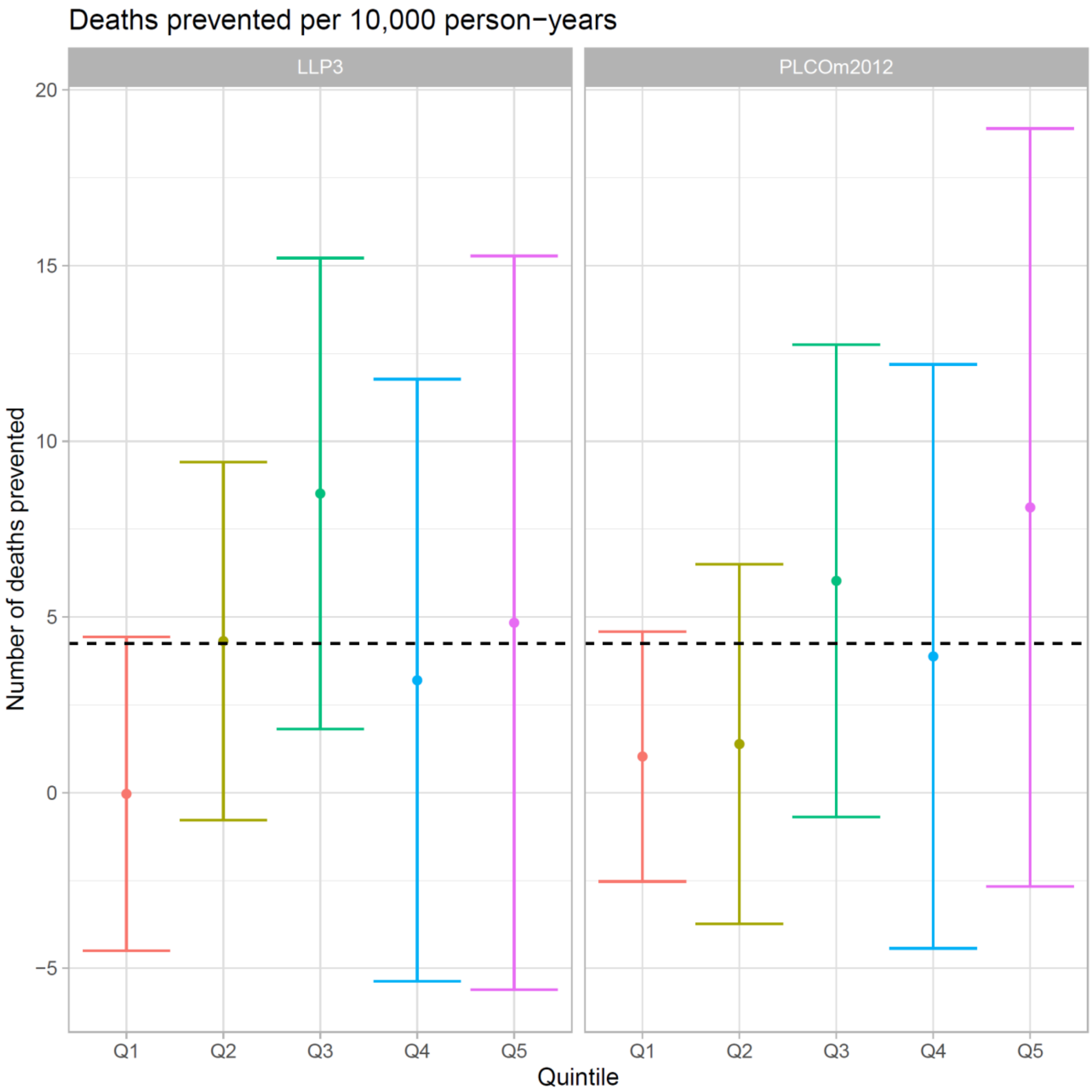

Figure notes: Based on N = 977 lung cancer deaths in NLST. The error bars represent the 95% confidence intervals.

**Quintile thresholds**	**Q1**	**Q2**	**Q3**	**Q4**	**Q5**
**Risk modelling (LLPv3 model)**	<0.51%	0.51% - 0.98%	0.98% - 1.61%	1.61% - 2.65%	>2.65%
**Risk modelling (PLCOm2012 model)**	<1.52%	1.52% - 2.25%	2.25% - 3.25%	3.25% - 5.12%	>5.12%

Figure S34: Lung cancer deaths prevented by risk-prediction model quintile based on NELSON in NLST

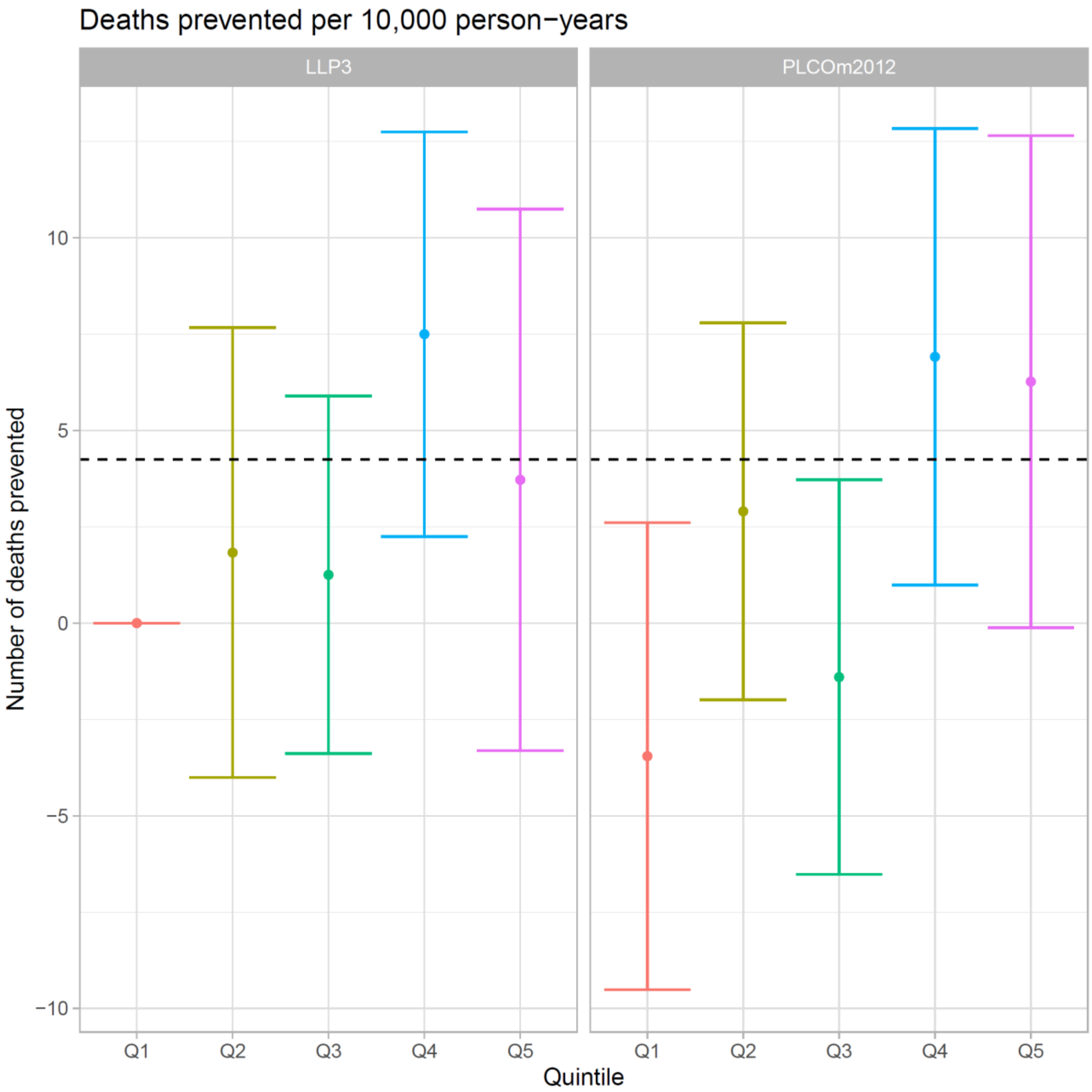

Figure notes: Based on N = 977 lung cancer deaths in NLST. The error bars represent the 95% confidence intervals.

Quintile thresholds	Q1	Q2	Q3	Q4	Q5
Risk modelling (LLPv3 model)	<0.27%	0.27% - 0.41%	0.41% - 0.75%	0.75% - 1.73%	>1.73%
Risk modelling (PLCOm2012 model)	<0.96%	0.96% - 1.42%	1.42% - 2.01%	2.01% - 3.02%	>3.02%

Figure S35: Lung cancer deaths prevented by risk-prediction model quintile based on NLST in NELSON

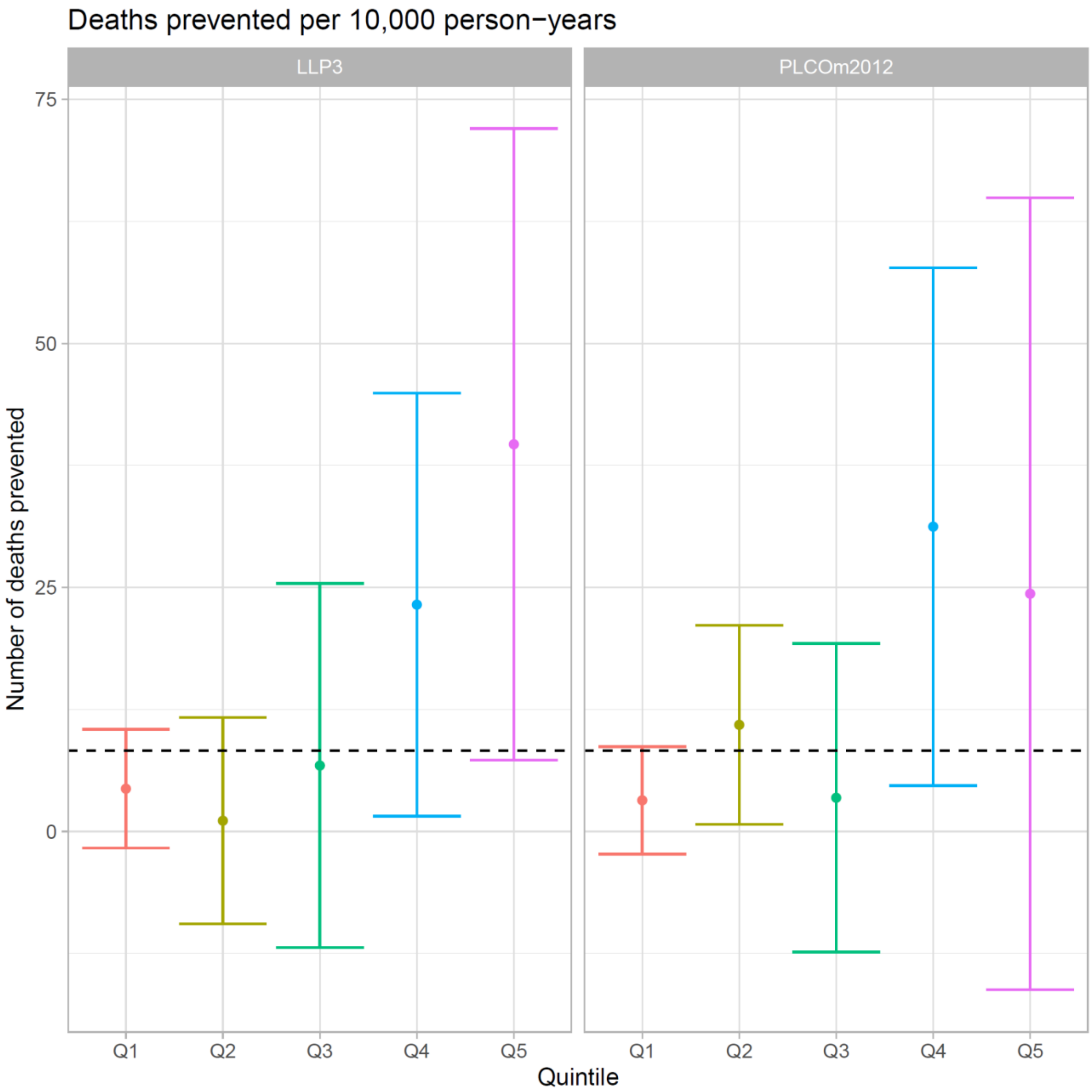

Figure notes: Based on N = 400 lung cancer deaths in NELSON. The error bars represent the 95% confidence intervals.

**Quintile thresholds**	**Q1**	**Q2**	**Q3**	**Q4**	**Q5**
**Risk modelling (LLPv3 model)**	<0.51%	0.51% - 0.98%	0.98% - 1.61%	1.61% - 2.65%	>2.65%
**Risk modelling (PLCOm2012 model)**	<1.52%	1.52% - 2.25%	2.25% - 3.25%	3.25% - 5.12%	>5.12%

Figure S36: Calibration for absolute benefit (overall LCM) for risk- and effect- models developed in NELSON

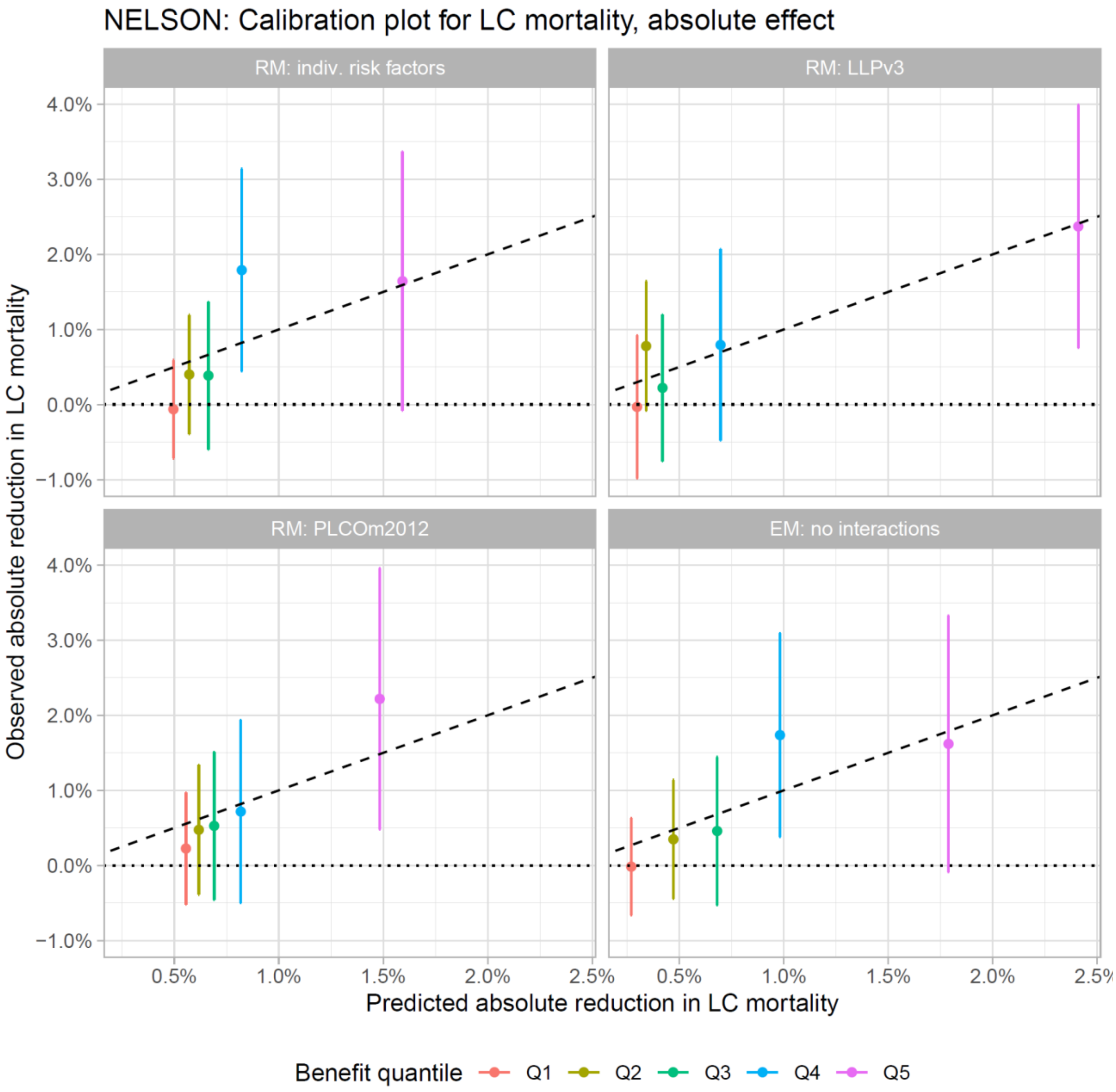

Figure notes: Based on N = 400 lung cancer deaths in NELSON. The error bars represent the 95% confidence intervals.

**Quintile thresholds**	**Q1**	**Q2**	**Q3**	**Q4**	**Q5**
**Risk modeling (individual risk-factors)**	<0.53%	0.53% - 0.61%	0.61% - 0.72%	0.72% - 0.96%	≥0.96%
**Risk modeling (LLPv3 model)**	<0.32%	0.32% - 0.37%	0.37% - 0.50%	0.50% - 1.02%	≥1.02%
**Risk modeling (PLCOm2012 model)**	<0.59%	0.59% - 0.65%	0.65% - 0.74%	0.74% - 0.92%	≥0.92%
**Effect modeling**	<0.38%	0.38% - 0.57%	0.57% - 0.81%	0.81% - 1.20%	≥1.20%

Figure S37: Calibration for absolute benefit (overall LCM) for risk and effect models developed in NLST

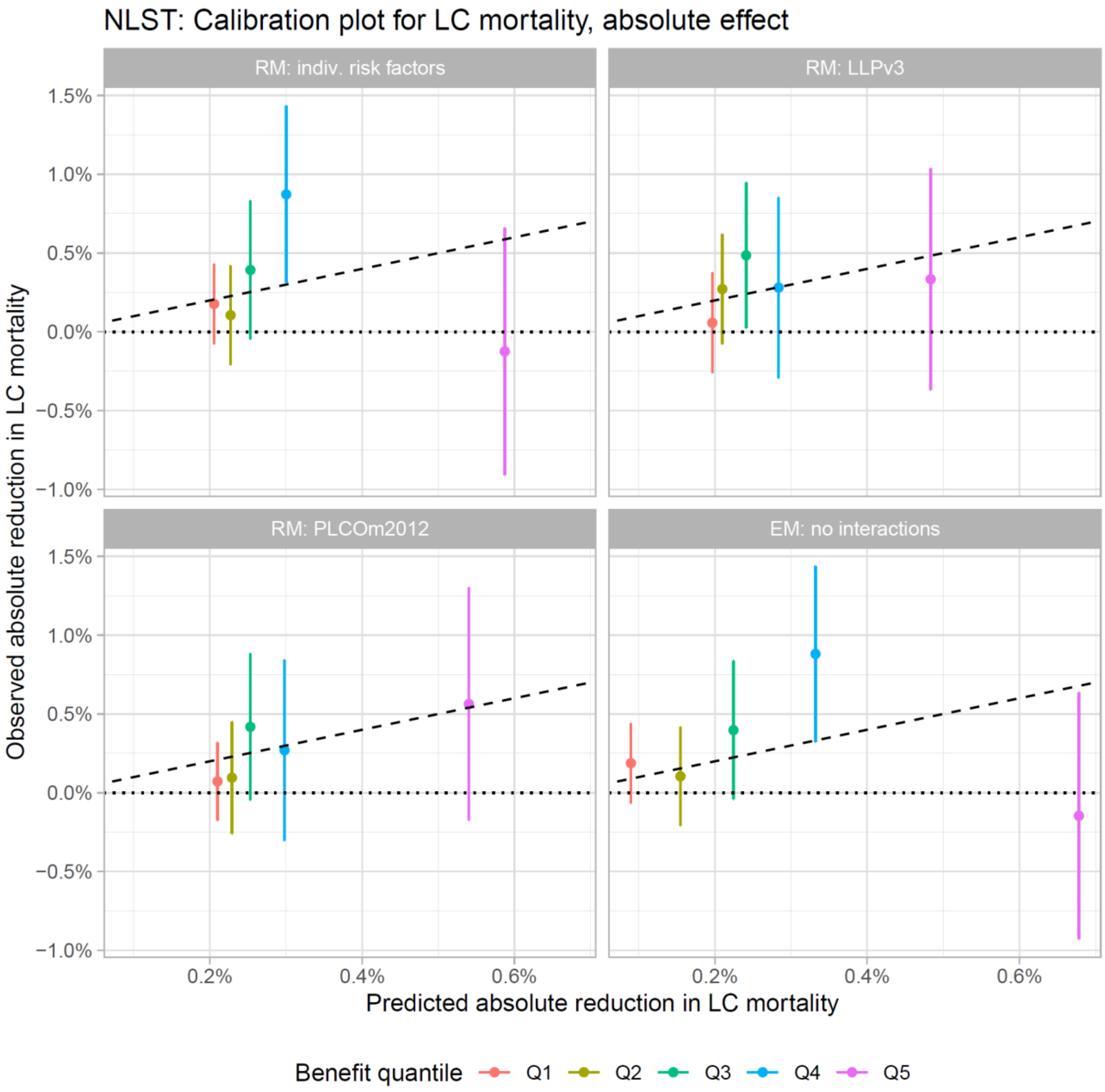

Figure notes: Based on N = 977 lung cancer deaths in NLST. The error bars represent the 95% confidence intervals.

**Quintile thresholds**	**Q1**	**Q2**	**Q3**	**Q4**	**Q5**
**Risk modeling (individual risk-factors)**	<0.22%	0.22% - 0.24%	0.24% - 0.27%	0.27% - 0.34%	≥0.34%
**Risk modeling (LLPv3 model)**	<0.20%	0.20% - 0.22%	0.22% - 0.26%	0.26% - 0.32%	≥0.32%
**Risk modeling (PLCOm2012 model)**	<0.22%	0.22% - 0.24%	0.24% - 0.27%	0.27% - 0.34%	≥0.34%
**Effect modeling**	<0.12%	0.12% - 0.19%	0.19% - 0.27%	0.27% - 0.42%	≥0.42%

Figure S38: Calibration for absolute benefit (adenocarcinoma-specific mortality) for risk- and effect- models developed in NELSON

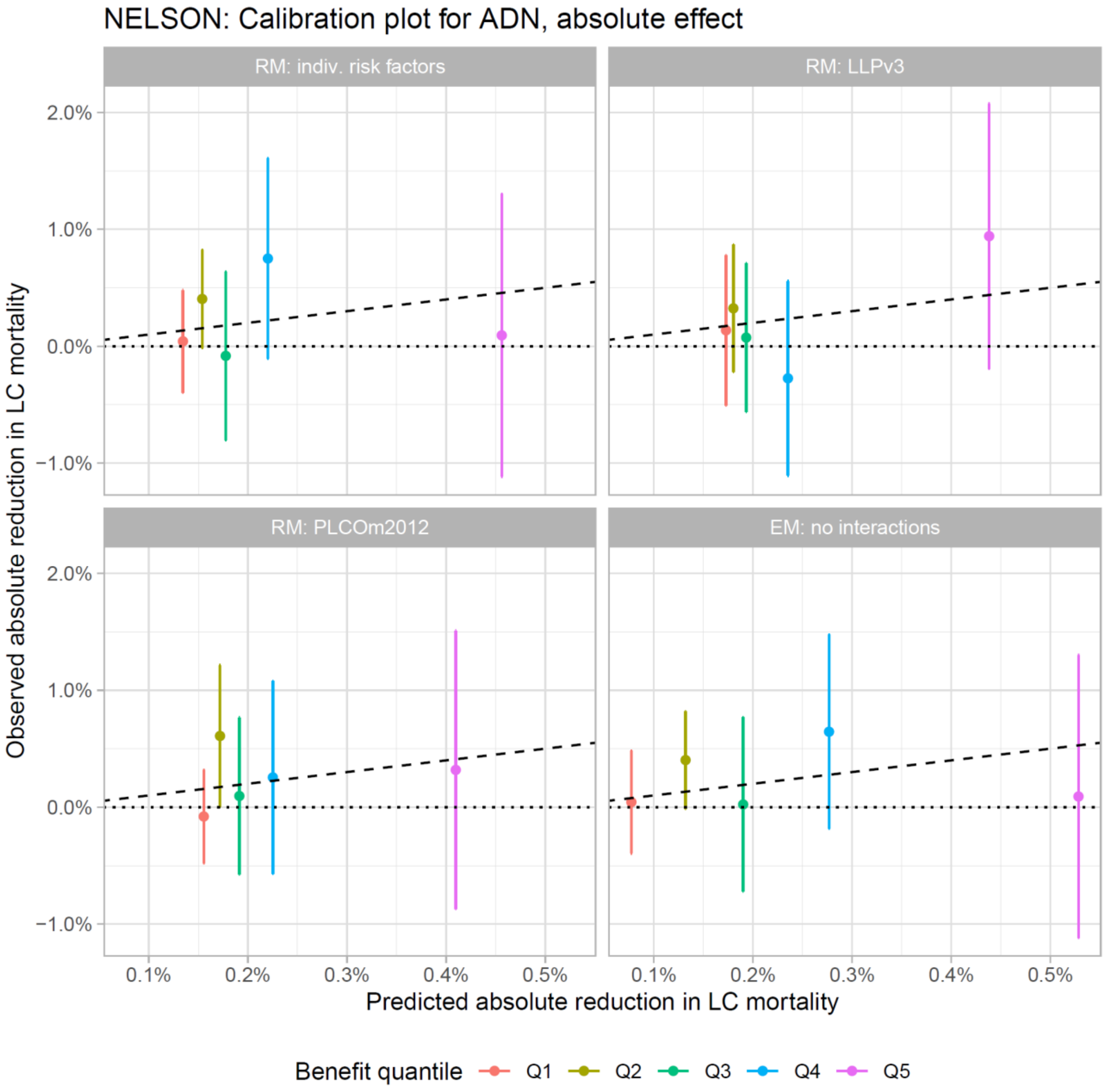

Figure notes: Based on N = 178 Adenocarcinoma deaths in NELSON. The error bars represent the 95% confidence intervals.

Quintile thresholds	Q1	Q2	Q3	Q4	Q5
Risk modeling (individual risk-factors)	<0.14%	0.14% - 0.16%	0.16% - 0.19%	0.19% - 0.26%	≥0.26%
Risk modeling (LLPv3 model)	<0.18%	0.18% - 0.18%	0.18% - 0.21%	0.21% - 0.28%	≥0.28%
Risk modeling (PLCOm2012 model)	<0.16%	0.16% - 0.18%	0.18% - 0.20%	0.20% - 0.25%	≥0.25%
Effect modeling	<0.11%	0.11% - 0.16%	0.16% - 0.23%	0.23% - 0.34%	≥0.34%

Figure S39: Calibration for absolute benefit (adenocarcinoma-specific mortality) for risk- and effect- models developed in NLST

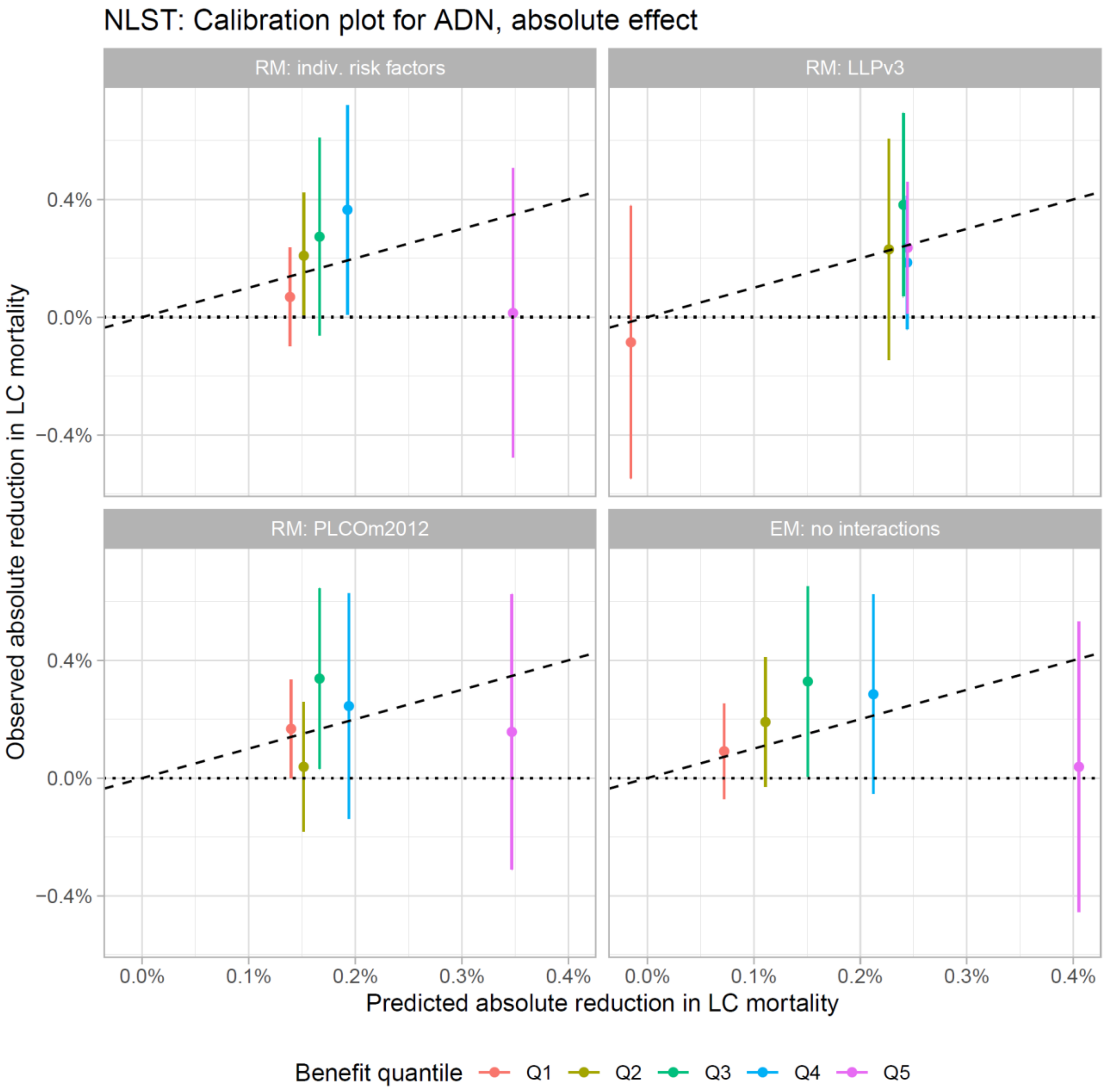

Figure notes: Based on N = 393 Adenocarcinoma deaths in NLST. The error bars represent the 95% confidence intervals.

Quintile thresholds	Q1	Q2	Q3	Q4	Q5
Risk modeling (individual risk-factors)	<0.15%	0.15% - 0.16%	0.16% - 0.18%	0.18% - 0.22%	≥0.22%
Risk modeling (LLPv3 model)	<0.21%	0.21% - 0.24%	0.24% - 0.24%	0.24% - 0.24%	≥0.24%
Risk modeling (PLCOm2012 model)	<0.15%	0.15% - 0.16%	0.16% - 0.18%	0.18% - 0.22%	≥0.22%
Effect modeling	<0.09%	0.09% - 0.13%	0.13% - 0.18%	0.18% - 0.26%	≥0.26%

Figure S40: Calibration for absolute benefit (squamous cell carcinoma-specific mortality) for risk- and effect- models developed in NELSON

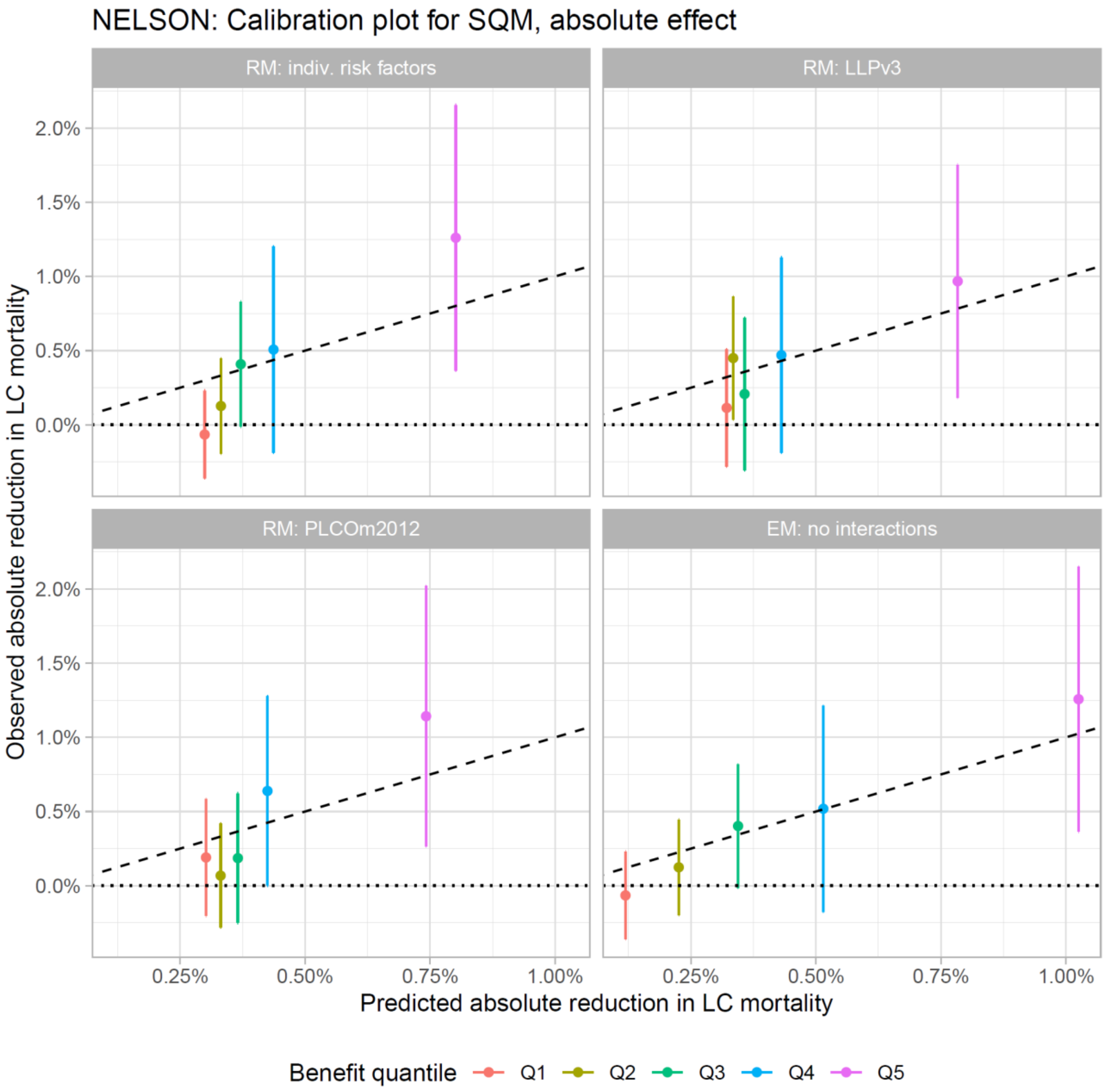

Figure notes: Based on N = 94 Squamous-cell carcinoma deaths in NELSON. The error bars represent the 95% confidence intervals.

Quintile thresholds	Q1	Q2	Q3	Q4	Q5
**Risk modeling (individual risk-factors)**	<0.31%	0.31% - 0.35%	0.35% - 0.40%	0.40% - 0.49%	≥0.49%
**Risk modeling (LLPv3 model)**	<0.33%	0.33% - 0.34%	0.34% - 0.38%	0.38% - 0.51%	≥0.51%
**Risk modeling (PLCOm2012 model)**	<0.32%	0.32% - 0.35%	0.35% - 0.39%	0.39% - 0.47%	≥0.47%
**Effect modeling**	<0.17%	0.17% - 0.28%	0.28% - 0.41%	0.41% - 0.64%	≥0.64%

Figure S41: Calibration for absolute benefit (squamous cell carcinoma-specific mortality) for risk- and effect- models developed in NLST

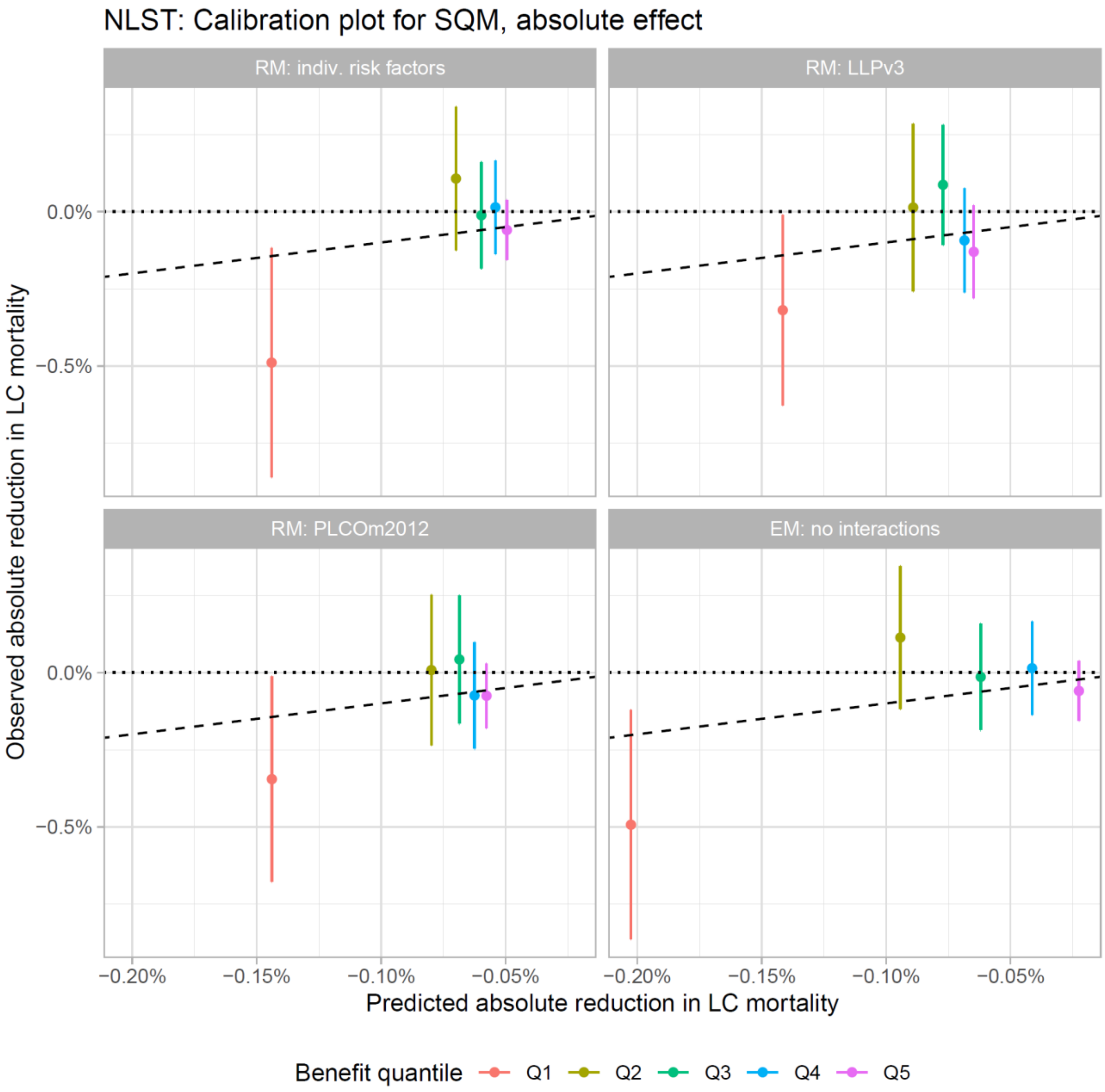

Figure notes: Based on N = 184 Squamous-cell carcinoma deaths in NLST. The error bars represent the 95% confidence intervals.

Quintile thresholds	Q1	Q2	Q3	Q4	Q5
**Risk modeling (individual risk-factors)**	<-0.08%	-0.08% - -0.06%	-0.06% - -0.06%	-0.06% - -0.05%	≥-0.05%
**Risk modeling (LLPv3 model)**	<-0.10%	-0.10% - -0.08%	-0.08% - -0.07%	-0.07% - -0.07%	≥-0.07%
**Risk modeling (PLCOm2012 model)**	<-0.09%	-0.09% - -0.07%	-0.07% - -0.07%	-0.07% - -0.06%	≥-0.06%
**Effect modeling**	<-0.12%	-0.12% - -0.07%	-0.07% - -0.05%	-0.05% - -0.03%	≥-0.03%

Figure S42: Calibration for absolute benefit (Others and non-small cell carcinoma not otherwise specified-specific mortality) for risk- and effect-models developed in NELSON

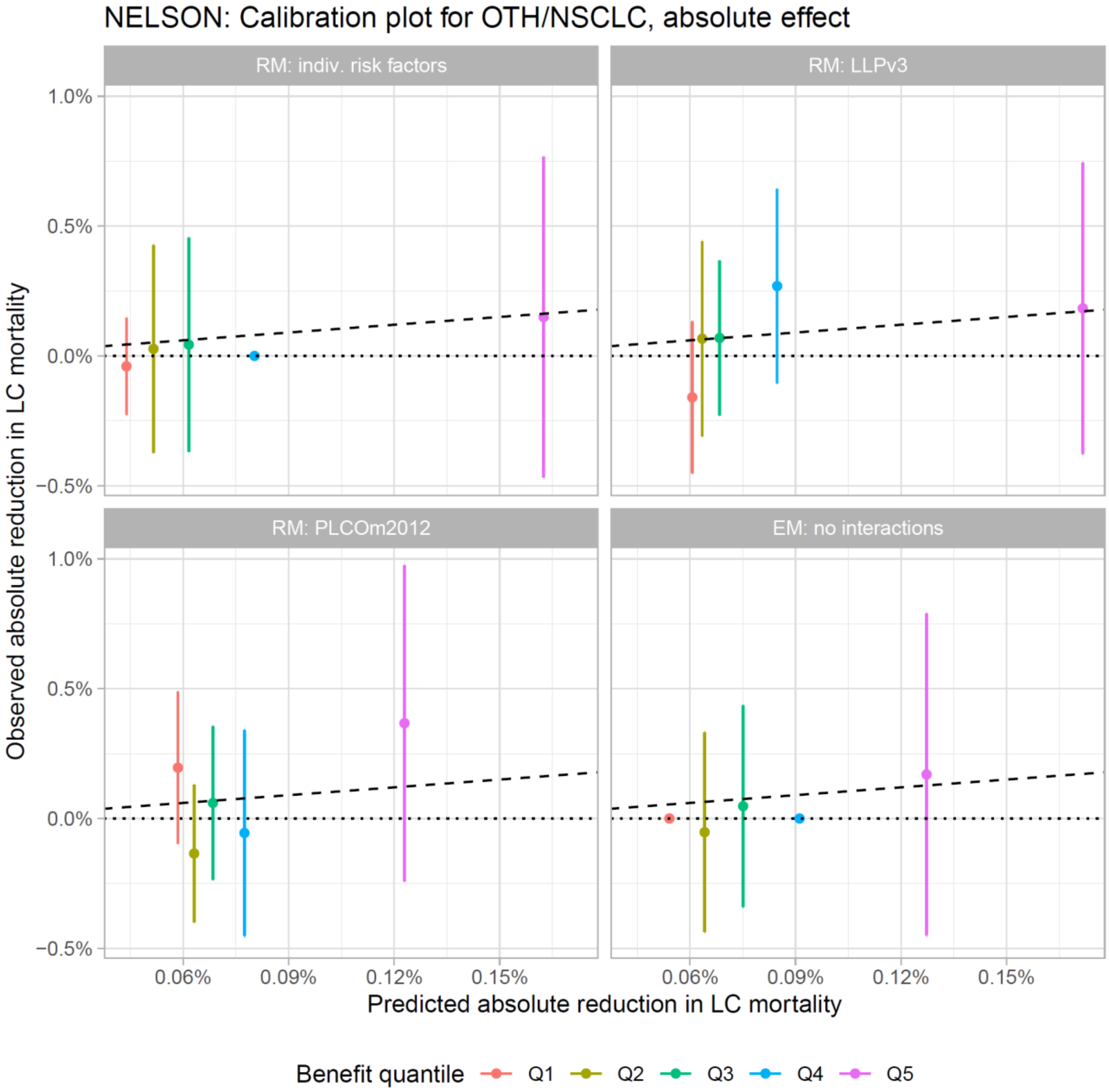

Figure notes: Based on N = 43 Other lung cancer deaths in NELSON. The error bars represent the 95% confidence intervals.

Quintile thresholds	Q1	Q2	Q3	Q4	Q5
**Risk modeling (individual risk-factors)**	<0.05%	0.05% - 0.06%	0.06% - 0.07%	0.07% - 0.10%	≥0.10%
**Risk modeling (LLPv3 model)**	<0.06%	0.06% - 0.07%	0.07% - 0.07%	0.07% - 0.10%	≥0.10%
**Risk modeling (PLCOm2012 model)**	<0.06%	0.06% - 0.07%	0.07% - 0.07%	0.07% - 0.08%	≥0.08%
**Effect modeling**	<0.06%	0.06% - 0.07%	0.07% - 0.08%	0.08% - 0.10%	≥0.10%

Figure S43: Calibration for absolute benefit (Others and non-small cell carcinoma not otherwise specified-specific mortality) for risk- and effect- models developed in NLST

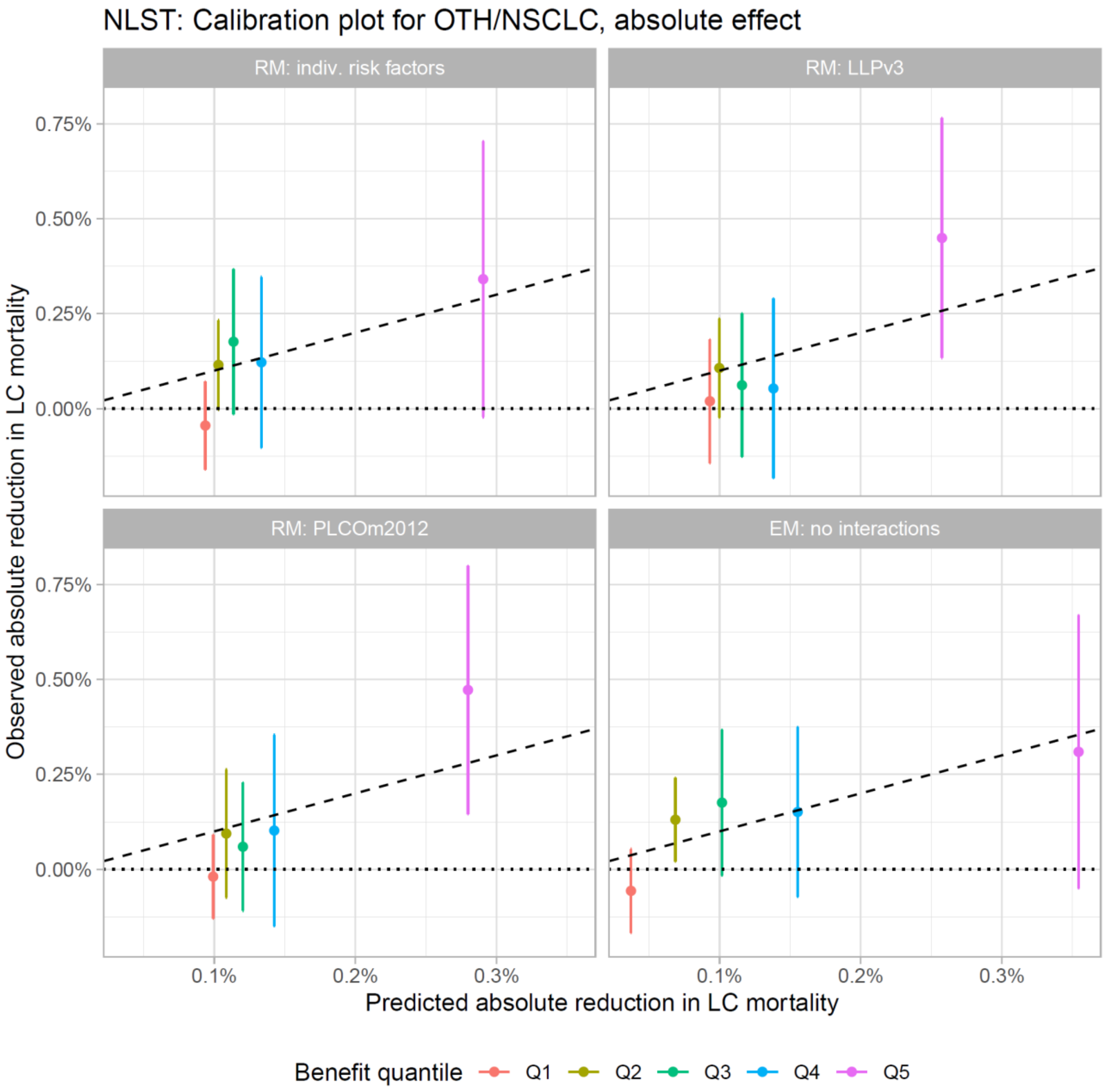

Figure notes: Based on N = 176 Other lung cancer deaths in NLST. The error bars represent the 95% confidence intervals.

Quintile thresholds	Q1	Q2	Q3	Q4	Q5
Risk modeling (individual risk-factors)	<0.10%	0.10% - 0.11%	0.11% - 0.12%	0.12% - 0.15%	≥0.15%
Risk modeling (LLPv3 model)	<0.10%	0.10% - 0.11%	0.11% - 0.12%	0.12% - 0.16%	≥0.16%
Risk modeling (PLCOm2012 model)	<0.10%	0.10% - 0.11%	0.11% - 0.13%	0.13% - 0.16%	≥0.16%
Effect modeling	<0.05%	0.05% - 0.08%	0.08% - 0.12%	0.12% - 0.20%	≥0.20%

Figure S44: Calibration for absolute benefit (Small cell carcinoma- specific mortality) for risk- and effect- models developed in NELSON

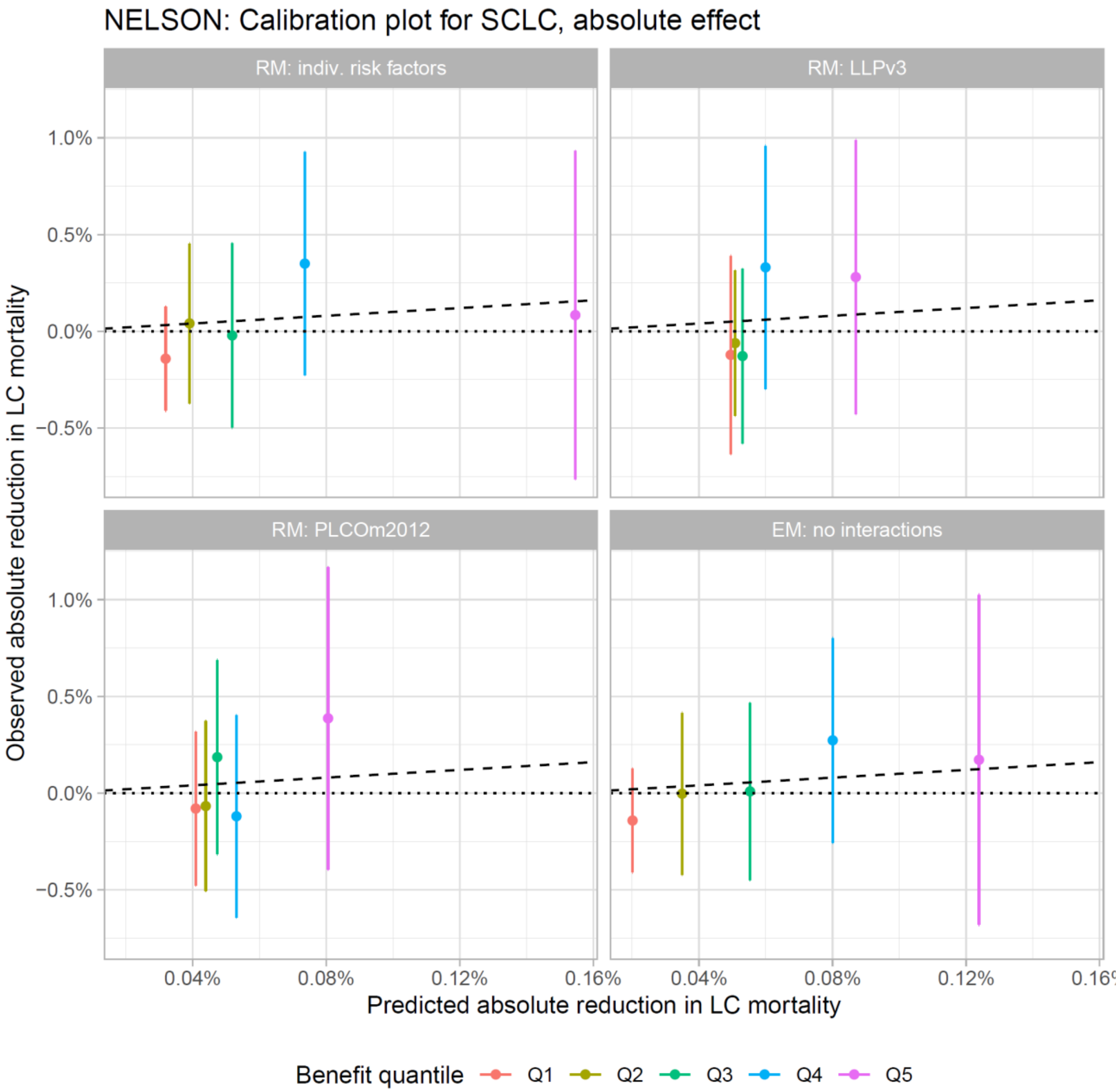

Figure notes: Based on N = 84 Small-cell carcinoma deaths in NELSON. The error bars represent the 95% confidence intervals.

Quintile thresholds	Q1	Q2	Q3	Q4	Q5
Risk modeling (individual risk-factors)	<0.04%	0.04% - 0.04%	0.04% - 0.06%	0.06% - 0.09%	≥0.09%
Risk modeling (LLPv3 model)	<0.05%	0.05% - 0.05%	0.05% - 0.06%	0.06% - 0.07%	≥0.07%
Risk modeling (PLCOm2012 model)	<0.04%	0.04% - 0.05%	0.05% - 0.05%	0.05% - 0.06%	≥0.06%
Effect modeling	<0.03%	0.03% - 0.04%	0.04% - 0.07%	0.07% - 0.09%	≥0.09%

Figure S45: Calibration for absolute benefit (Small cell carcinoma- specific mortality) for risk- and effect- models developed in NLST

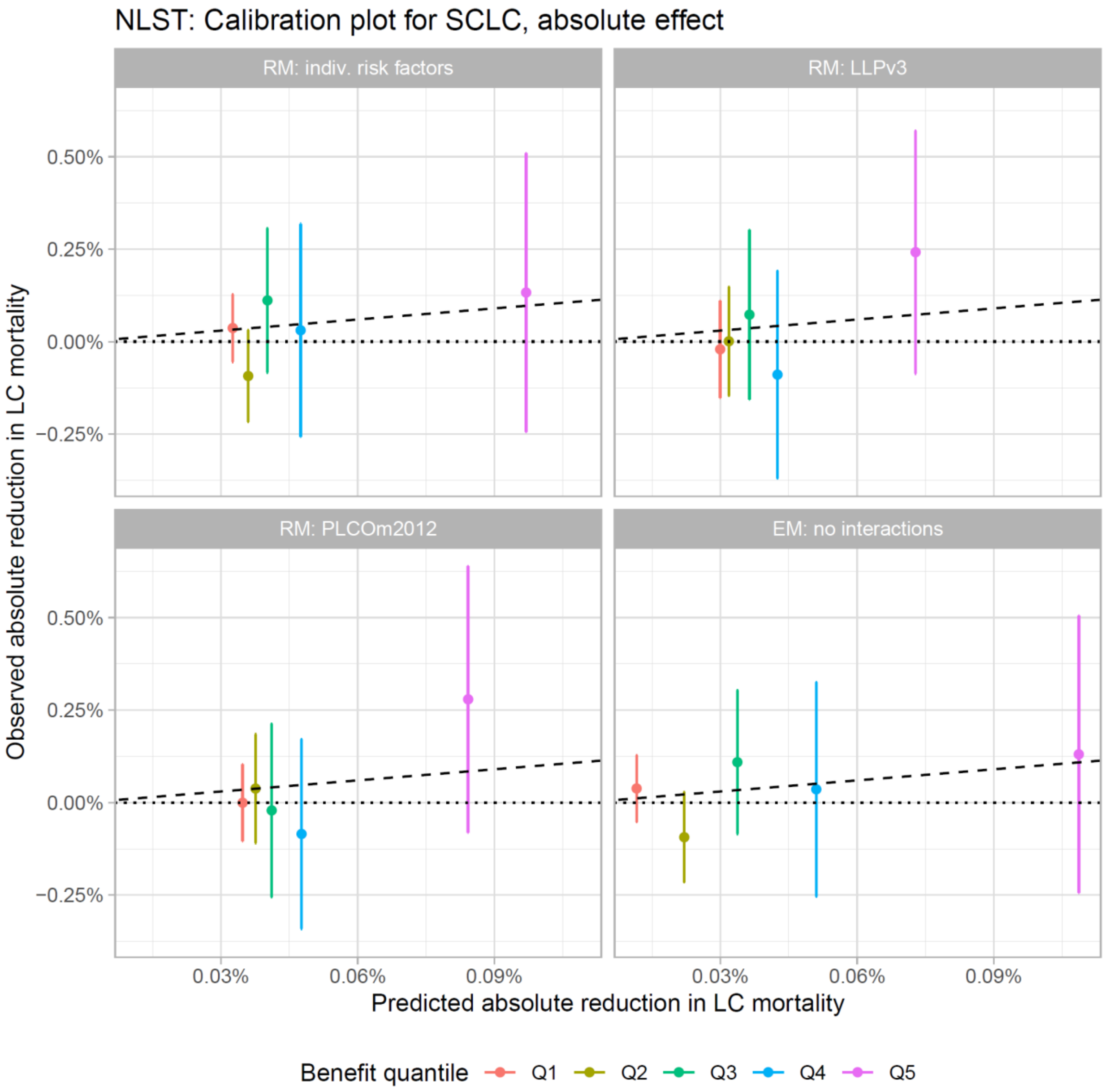

Figure notes: Based on N = 209 Small-cell carcinoma deaths in NLST. The error bars represent the 95% confidence intervals.

**Quintile thresholds**	**Q1**	**Q2**	**Q3**	**Q4**	**Q5**
**Risk modeling (individual risk-factors)**	<0.03%	0.03% - 0.04%	0.04% - 0.04%	0.04% - 0.05%	≥0.05%
**Risk modeling (LLPv3 model)**	<0.03%	0.03% - 0.03%	0.03% - 0.04%	0.04% - 0.05%	≥0.05%
**Risk modeling (PLCOm2012 model)**	<0.04%	0.04% - 0.04%	0.04% - 0.04%	0.04% - 0.05%	≥0.05%
**Effect modeling**	<0.02%	0.02% - 0.03%	0.03% - 0.04%	0.04% - 0.06%	≥0.06%

Figure S46: Calibration for absolute benefit (overall LCM) for causal forests developed in NELSON and NLST

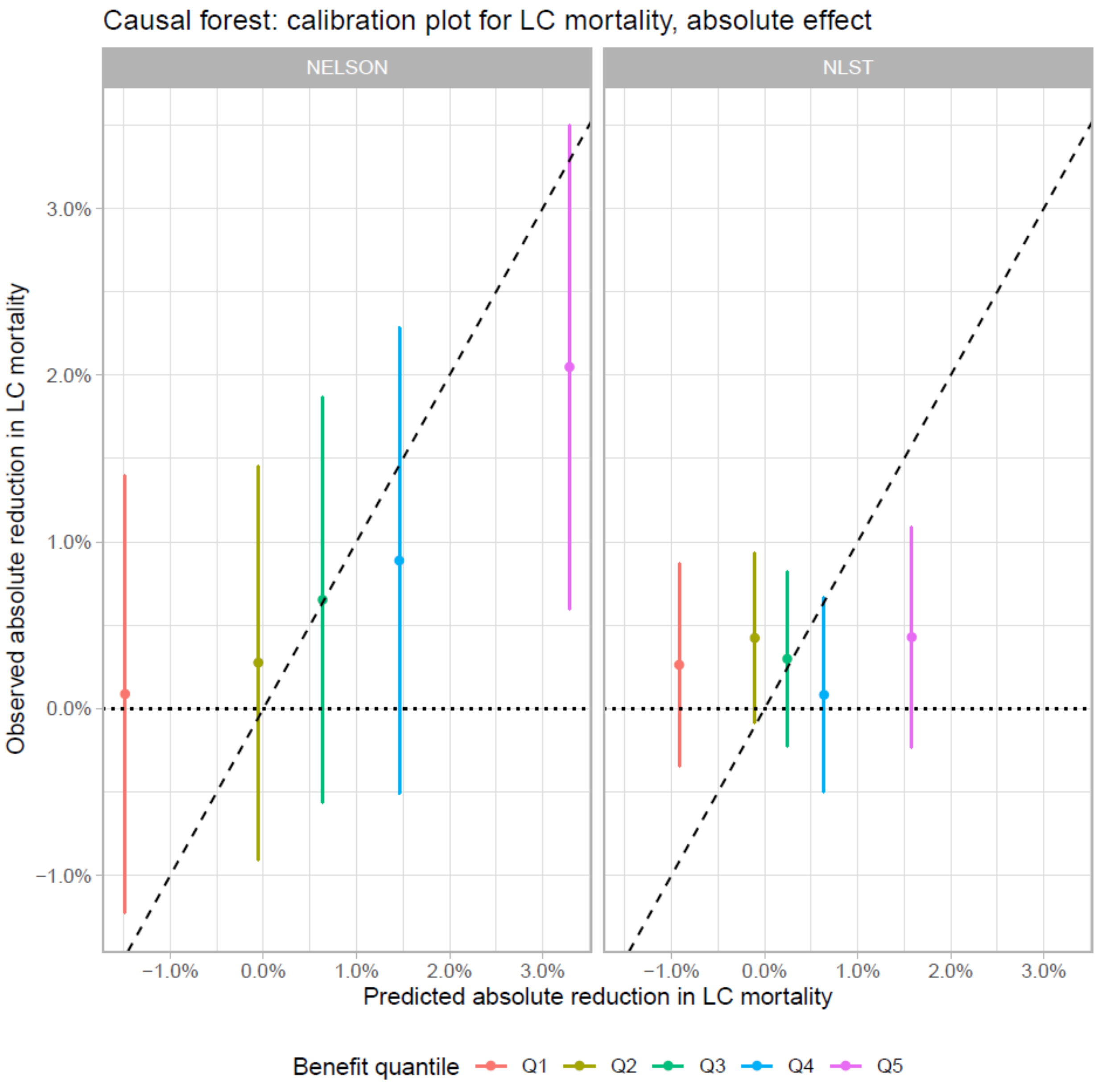

Figure notes: Based on N = 400 lung cancer deaths in NELSON and N = 977 lung cancer deaths in NLST. The error bars represent the 95% confidence intervals.

**Quintile thresholds**	**Q1**	**Q2**	**Q3**	**Q4**	**Q5**
**NELSON**	< -0.45%	-0.45%-0.30%	0.30%-1.00%	1.00%-2.01%	>2.01%
**NLST**	< -0.31%	-0.31%-0.08%	0.08%-0.41%	0.41%-0.89%	>0.89%

Figure S47: Calibration for absolute benefit (adenocarcinoma-specific mortality) for causal forests developed in NELSON and NLST

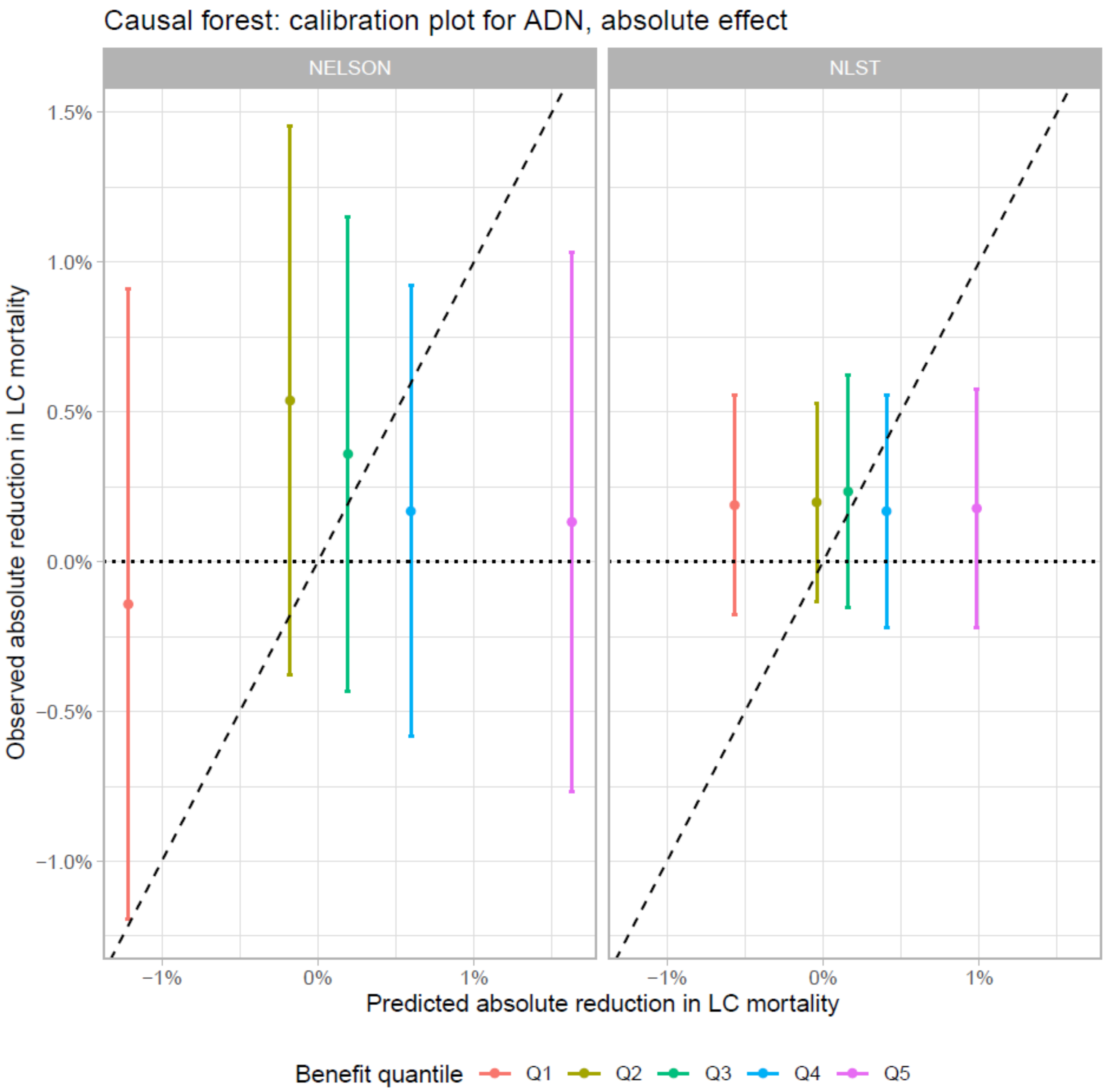

Figure notes: Based on N = 178 Adenocarcinoma deaths in NELSON and N = 393 Adenocarcinoma deaths in NLST. The error bars represent the 95% confidence intervals.

**Quintile thresholds**	**Q1**	**Q2**	**Q3**	**Q4**	**Q5**
**NELSON**	< -0.43%	-0.43%-0.03%	0.03%-0.37%	0.37%-0.86%	>0.86%
**NLST**	< -0.16%	-0.16%-0.06%	0.06%-0.26%	0.26%-0.56%	>0.56%

Figure S48: Calibration for absolute benefit (squamous cell-specific mortality) for causal forests developed in NELSON and NLST

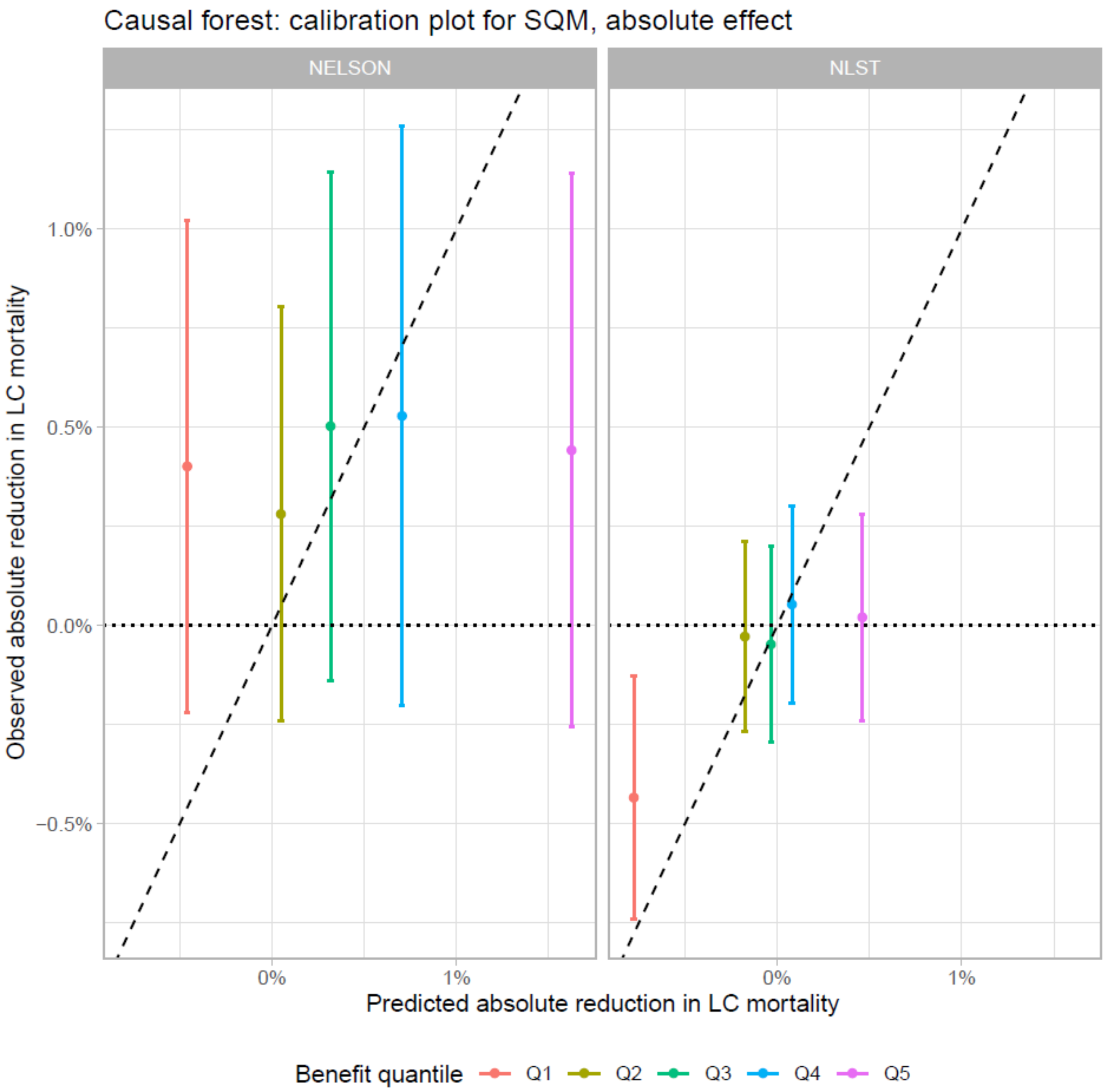

Figure notes: Based on N = 94 Squamous-cell carcinoma deaths in NELSON and N = 184 Squamous-cell carcinoma deaths in NLST.

The error bars represent the 95% confidence intervals.

Quintile thresholds	Q1	Q2	Q3	Q4	Q5
NELSON	< -0.06%	-0.06%-0.17%	0.17%-0.49%	0.49%-0.97%	>0.97%
NLST	< -0.29%	-0.29% - -0.08%	-0.08% - 0.01%	0.01%-0.16%	>0.16%

Figure S49: Calibration for absolute benefit (Others and non-small cell carcinoma not otherwise specified-specific mortality) for causal forests developed in NELSON and NLST

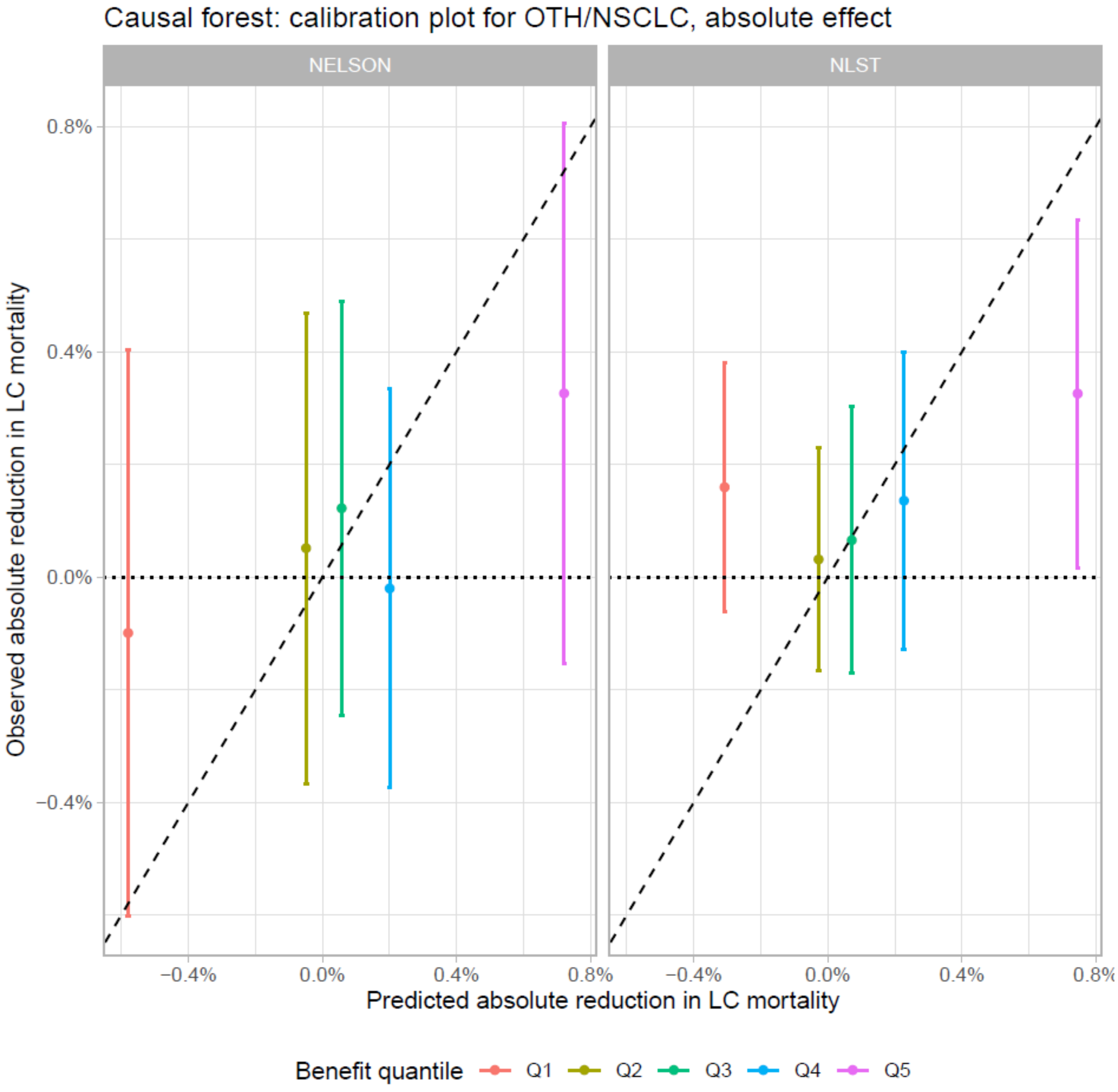

Figure notes: Based on N = 43 Other lung cancer deaths in NELSON and N = 176 Other lung cancer deaths in NLST. The error bars represent the 95% confidence intervals.

Quintile thresholds	Q1	Q2	Q3	Q4	Q5
NELSON	< -0.14%	-0.14%-0.01%	0.01%-0.11%	0.11%-0.31%	>0.31%
NLST	< -0.08%	-0.08%-0.02%	0.02%-0.13%	0.13%-0.35%	>0.35%

Figure S50: Calibration for absolute benefit (small cell-specific mortality) for causal forests developed in NELSON and NLST

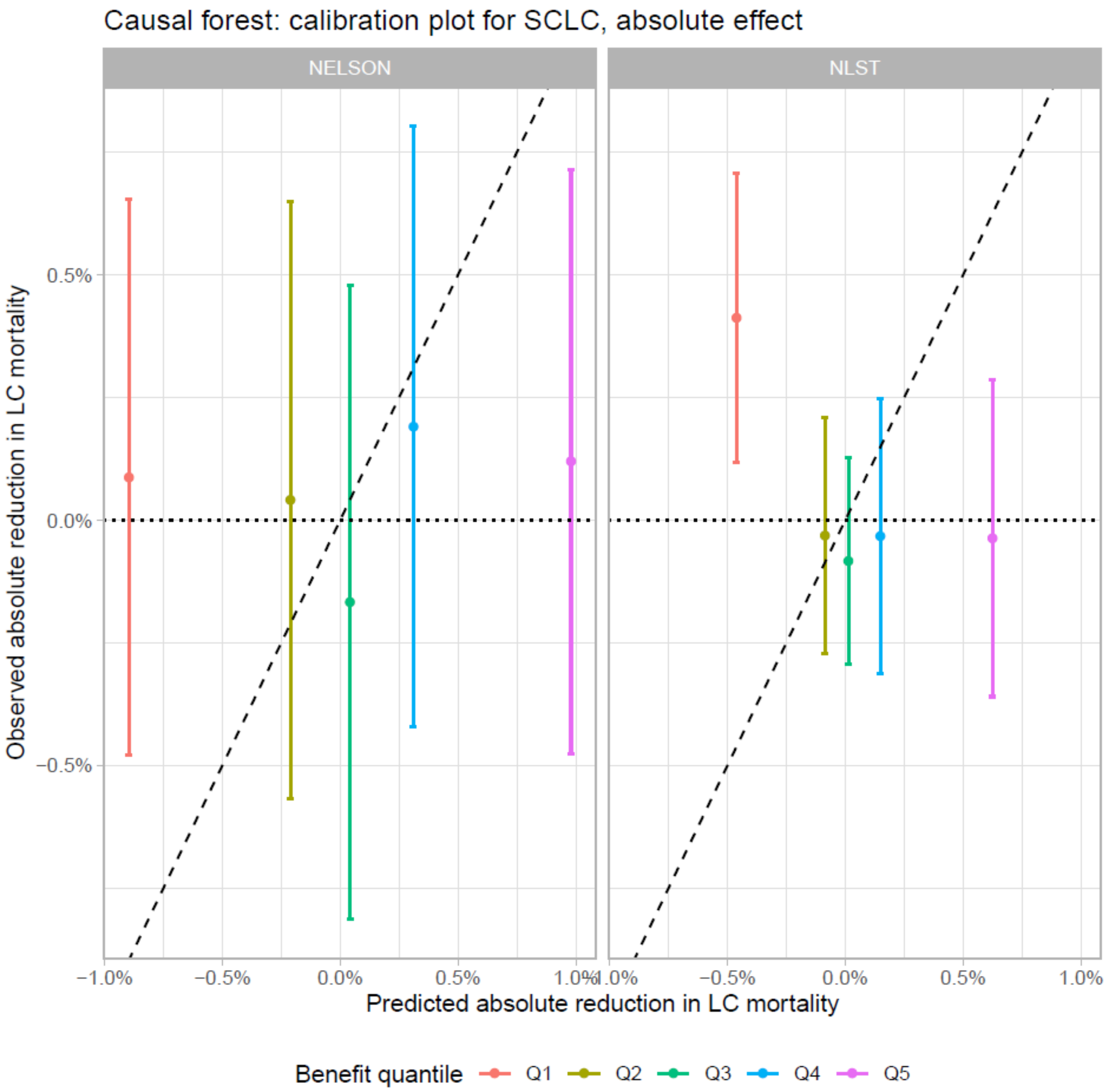

Figure notes: Based on N = 84 Small-cell carcinoma deaths in NELSON and N = 209 Small-cell carcinoma deaths in NLST.The error bars represent the 95% confidence intervals.

**Quintile thresholds**	**Q1**	**Q2**	**Q3**	**Q4**	**Q5**
**NELSON**	< -0.38%	-0.38% - -0.07%	-0.07%-0.16%	0.16%-0.50%	>0.50%
**NLST**	< -0.16%	-0.16% - 0.02%	-0.02%- 0.05%	0.05%-0.27%	>0.27%

Figure S51: Calibration for relative benefit (overall LCM) for risk- and effect-models developed in NELSON and validation in NLST

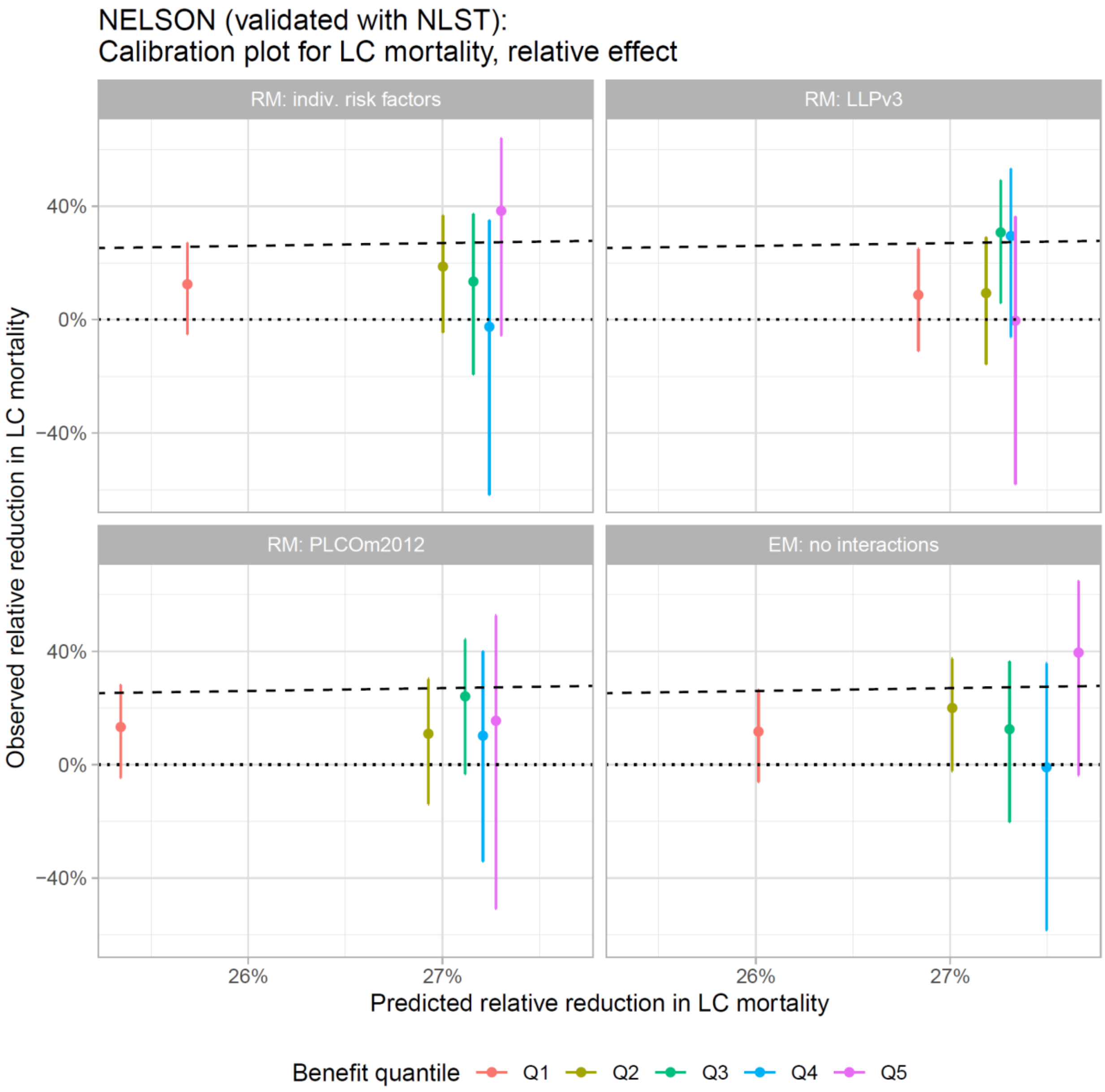

Figure notes: Based on N = 977 lung cancer deaths in NLST. The error bars represent the 95% confidence intervals.

**Quintile thresholds**	**Q1**	**Q2**	**Q3**	**Q4**	**Q5**
**Risk modeling (individual risk-factors)**	<26.90%	26.90% - 27.10%	27.10% - 27.20%	27.20% - 27.30%	≥27.30%
**Risk modeling (LLPv3 model)**	<27.12%	27.12% - 27.23%	27.23% - 27.29%	27.29% - 27.33%	≥27.33%
**Risk modeling (PLCOm2012 model)**	<26.74%	26.74% - 27.05%	27.05% - 27.17%	27.17% - 27.24%	≥27.24%
**Effect modeling**	<26.80%	26.80% - 27.20%	27.20% - 27.40%	27.40% - 27.60%	≥27.60%

Figure S52: Calibration for relative benefit (overall LCM) for risk- and effect-models developed in NLST and validation in NELSON

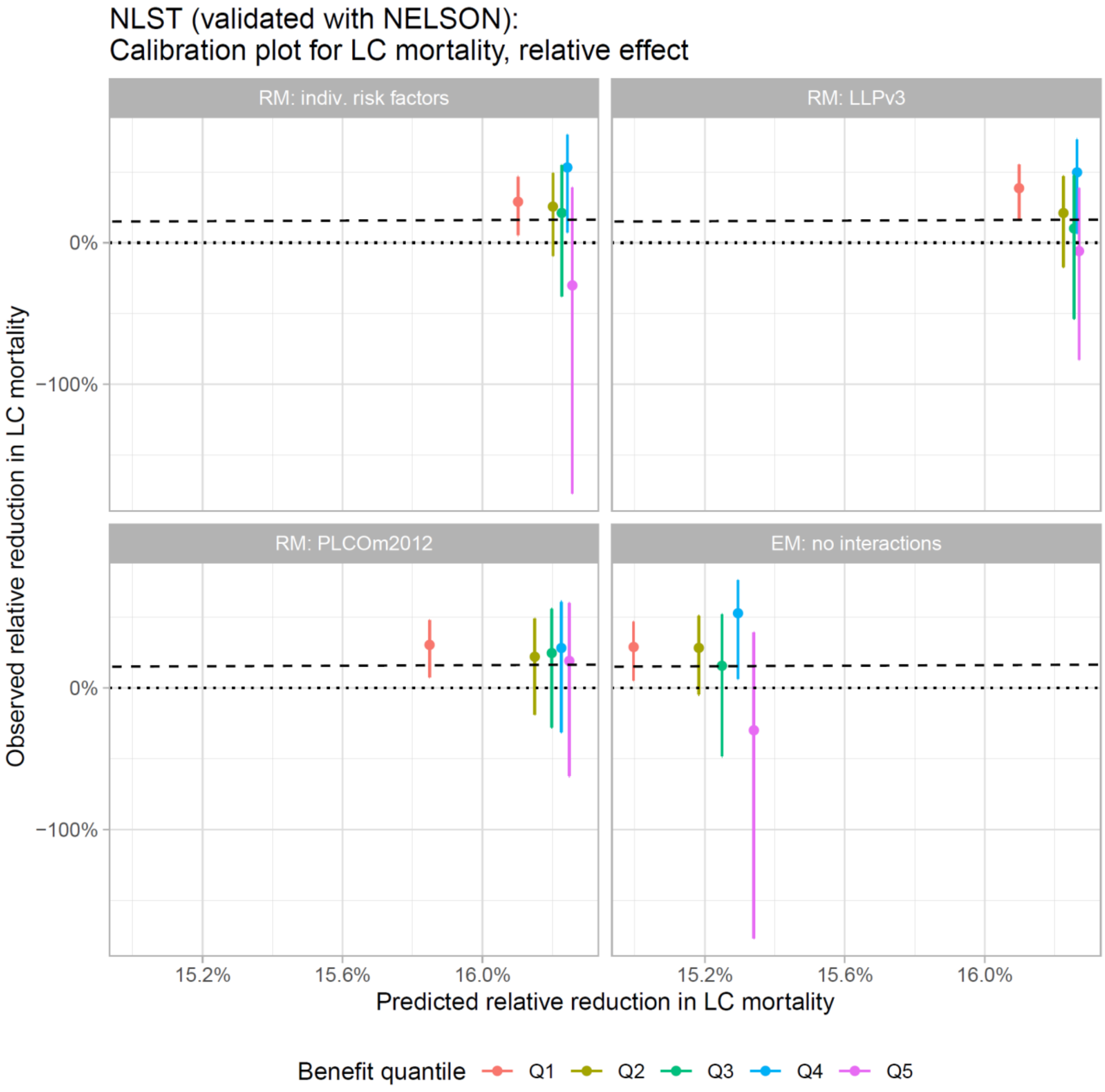

Figure notes: Based on N = 400 lung cancer deaths in NELSON. The error bars represent the 95% confidence intervals.

**Quintile thresholds**	**Q1**	**Q2**	**Q3**	**Q4**	**Q5**
**Risk modeling (individual risk-factors)**	<16.18%	16.18% - 16.22%	16.22% - 16.24%	16.24% - 16.25%	≥16.25%
**Risk modeling (LLPv3 model)**	<16.20%	16.20% - 16.25%	16.25% - 16.26%	16.26% - 16.27%	≥16.27%
**Risk modeling (PLCOm2012 model)**	<16.11%	16.11% - 16.18%	16.18% - 16.21%	16.21% - 16.24%	≥16.24%
**Effect modeling**	<15.13%	15.13% - 15.22%	15.22% - 15.27%	15.27% - 15.32%	≥15.32%

Figure S53: Calibration for relative benefit (adenocarcinoma-specific mortality) for risk- and effect-models developed in NELSON and validation in NLST

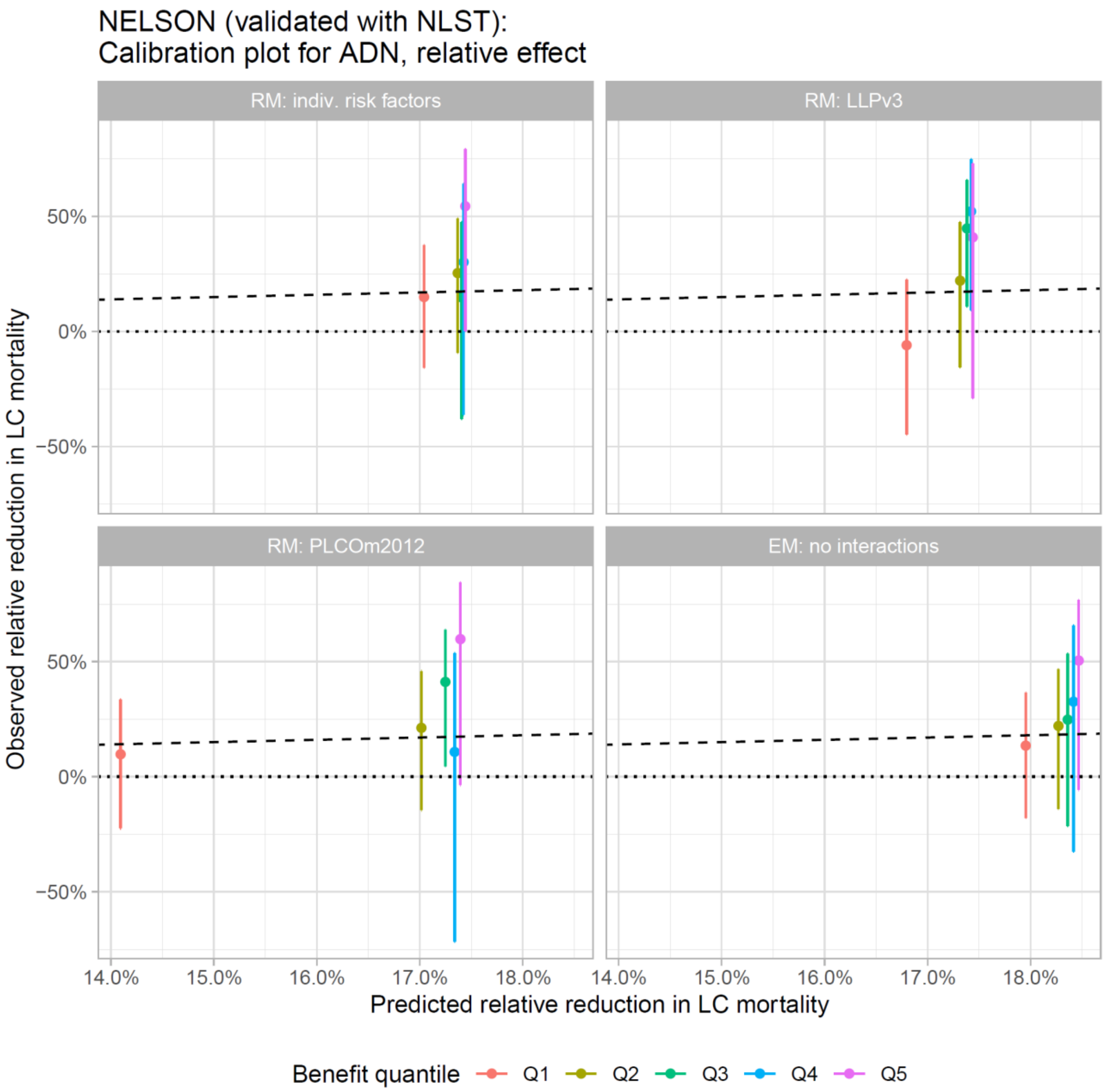

Figure notes: Based on N = 393 Adenocarcinoma deaths in NLST. The error bars represent the 95% confidence intervals.

Quintile thresholds	Q1	Q2	Q3	Q4	Q5
**Risk modeling (individual risk-factors)**	<17.33%	17.33% - 17.39%	17.39% - 17.42%	17.42% - 17.43%	≥17.43%
**Risk modeling (LLPv3 model)**	<17.26%	17.26% - 17.36%	17.36% - 17.41%	17.41% - 17.43%	≥17.43%
**Risk modeling (PLCOm2012 model)**	<16.70%	16.70% - 17.20%	17.20% - 17.30%	17.30% - 17.40%	≥17.40%
**Effect modeling**	<18.20%	18.20% - 18.32%	18.32% - 18.39%	18.39% - 18.44%	≥18.44%

Figure S54: Calibration for relative benefit (adenocarcinoma-specific mortality) for risk- and effect-models developed in NLST and validation in NELSON

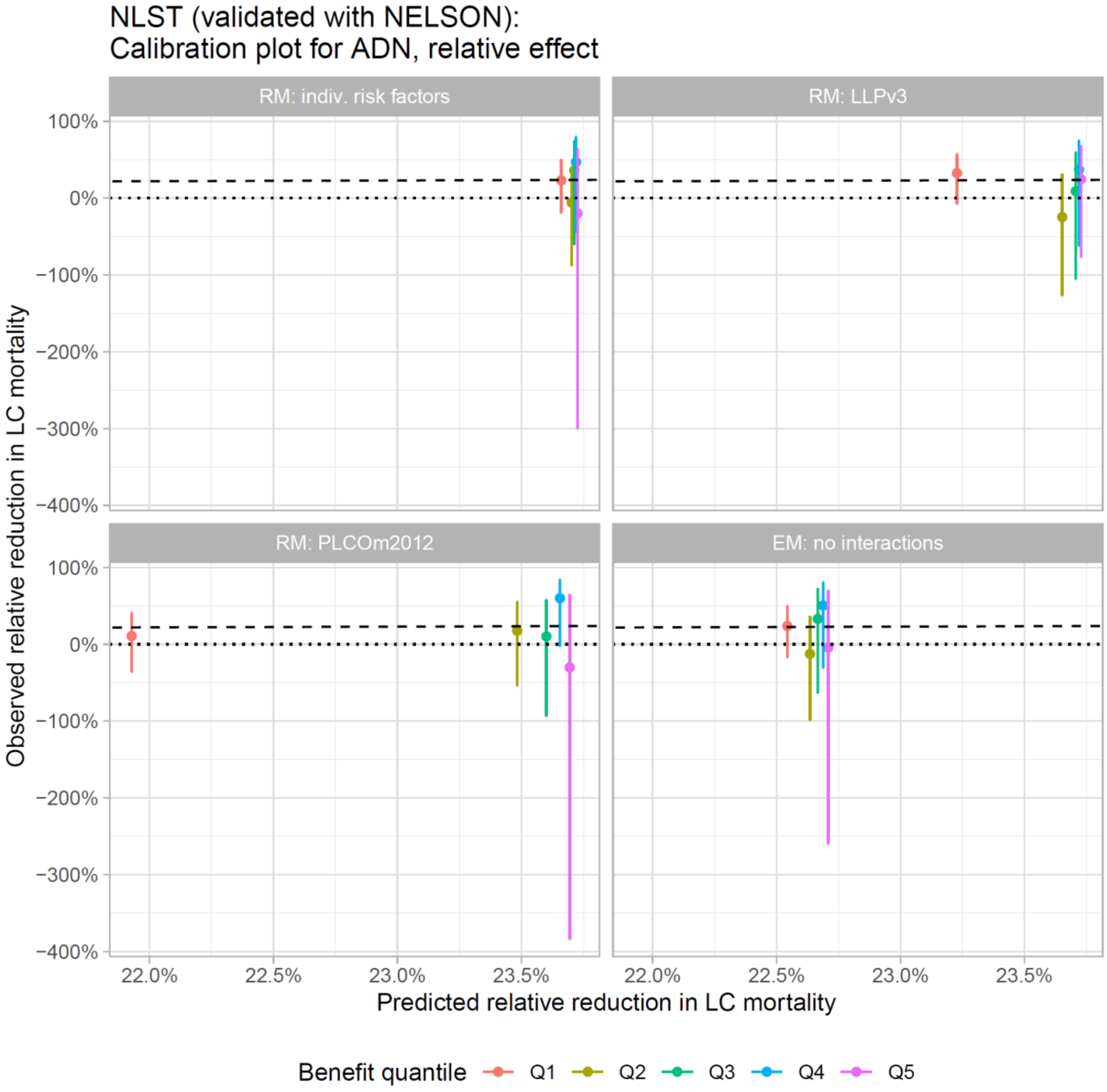

Figure notes: Based on N = 178 Adenocarcinoma deaths in NELSON. The error bars represent the 95% confidence intervals.

Quintile thresholds	Q1	Q2	Q3	Q4	Q5
**Risk modeling (individual risk-factors)**	<23.70%	23.70% - 23.71%	23.71% - 23.72%	23.72% - 23.72%	≥23.72%
**Risk modeling (LLPv3 model)**	<23.60%	23.60% - 23.69%	23.69% - 23.72%	23.72% - 23.72%	≥23.72%
**Risk modeling (PLCOm2012 model)**	<23.37%	23.37% - 23.56%	23.56% - 23.63%	23.63% - 23.68%	≥23.68%
**Effect modeling**	<22.61%	22.61% - 22.65%	22.65% - 22.68%	22.68% - 22.70%	≥22.70%

Figure S55: Calibration for relative benefit (squamous cell carcinoma-specific mortality) for risk- and effect-models developed in NELSON and validation in NLST

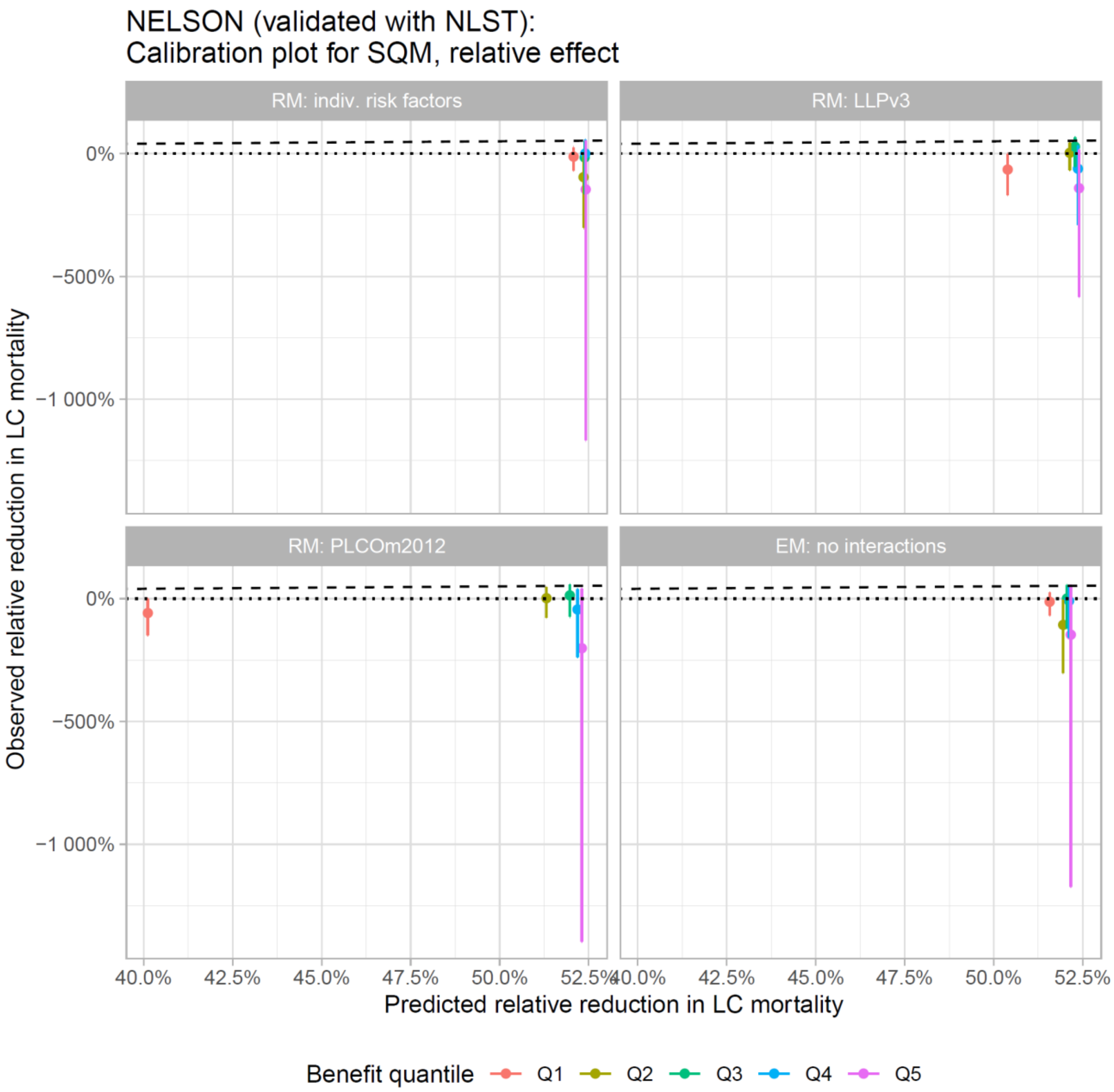

Figure notes: Based on N = 184 Squamous-cell carcinoma deaths in NLST. The error bars represent the 95% confidence intervals.

**Quintile thresholds**	**Q1**	**Q2**	**Q3**	**Q4**	**Q5**
**Risk modeling (individual risk-factors)**	<52.33%	52.33% - 52.38%	52.38% - 52.40%	52.40% - 52.41%	≥52.41%
**Risk modeling (LLPv3 model)**	<52.00%	52.00% - 52.20%	52.20% - 52.30%	52.30% - 52.40%	≥52.40%
**Risk modeling (PLCOm2012 model)**	<50.50%	50.50% - 51.80%	51.80% - 52.10%	52.10% - 52.30%	≥52.30%
**Effect modeling**	<51.87%	51.87% - 52.01%	52.01% - 52.09%	52.09% - 52.14%	≥52.14%

Figure S56: Calibration for relative benefit (squamous cell carcinoma-specific mortality) for risk- and effect-models developed in NLST and validation in NELSON

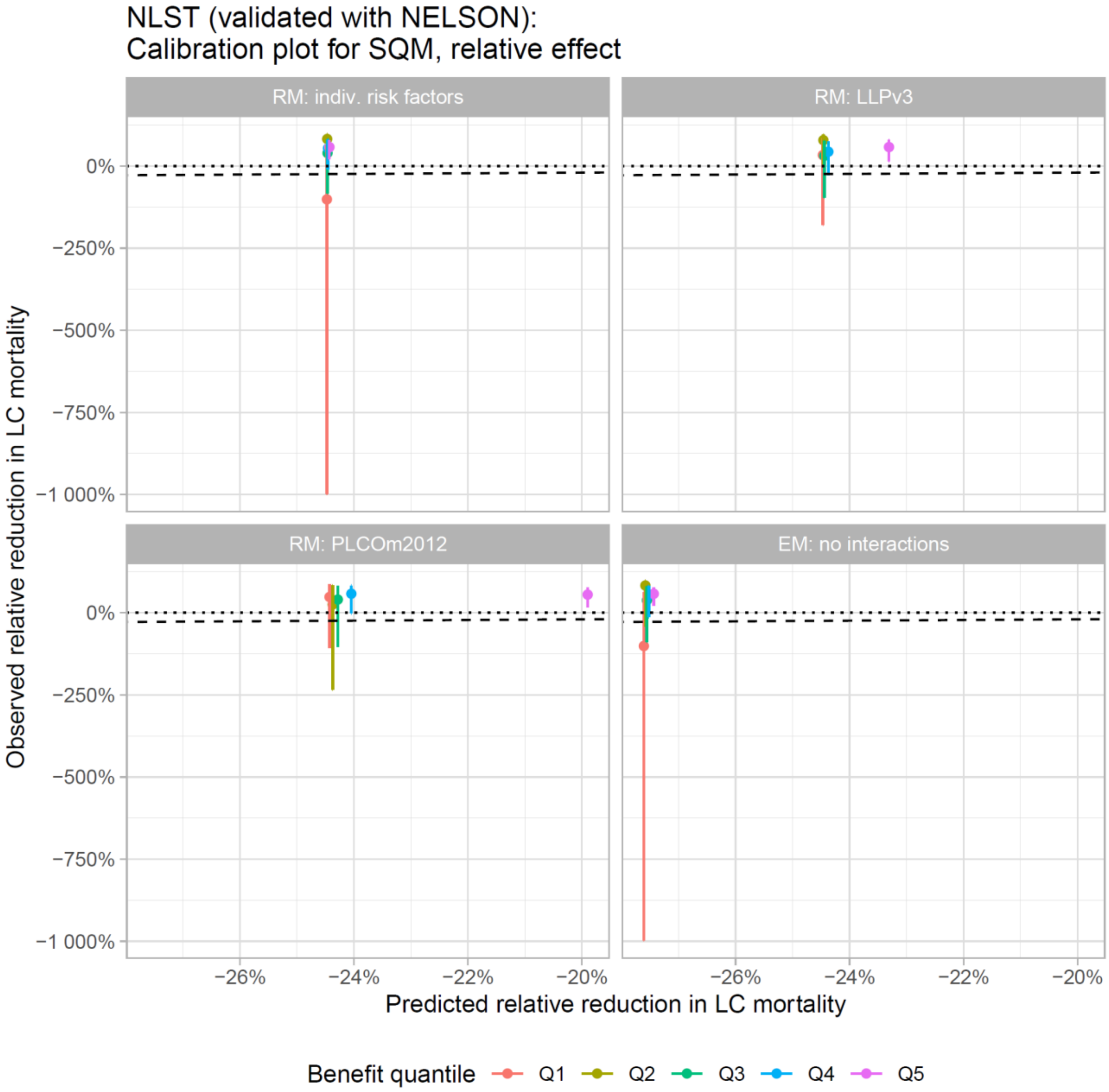

Figure notes: Based on N = 94 Squamous-cell carcinoma deaths in NELSON. The error bars represent the 95% confidence intervals.

**Quintile thresholds**	**Q1**	**Q2**	**Q3**	**Q4**	**Q5**
**Risk modeling (individual risk-factors)**	<-24.47%	-24.47% - -24.47%	-24.47% - -24.46%	-24.46% - -24.45%	≥-24.45%
**Risk modeling (LLPv3 model)**	<-24.46%	-24.46% - -24.45%	-24.45% - -24.43%	-24.43% - -24.28%	≥-24.28%
**Risk modeling (PLCOm2012 model)**	<-24.40%	-24.40% - -24.30%	-24.30% - -24.20%	-24.20% - -23.80%	≥-23.80%
**Effect modeling**	<-27.60%	-27.60% - -27.57%	-27.57% - -27.54%	-27.54% - -27.50%	≥-27.50%

Figure S57: Calibration for relative benefit (Others and non-small cell carcinoma not otherwise specified-specific mortality) for risk- and effect-models developed in NELSON and validation in NLST

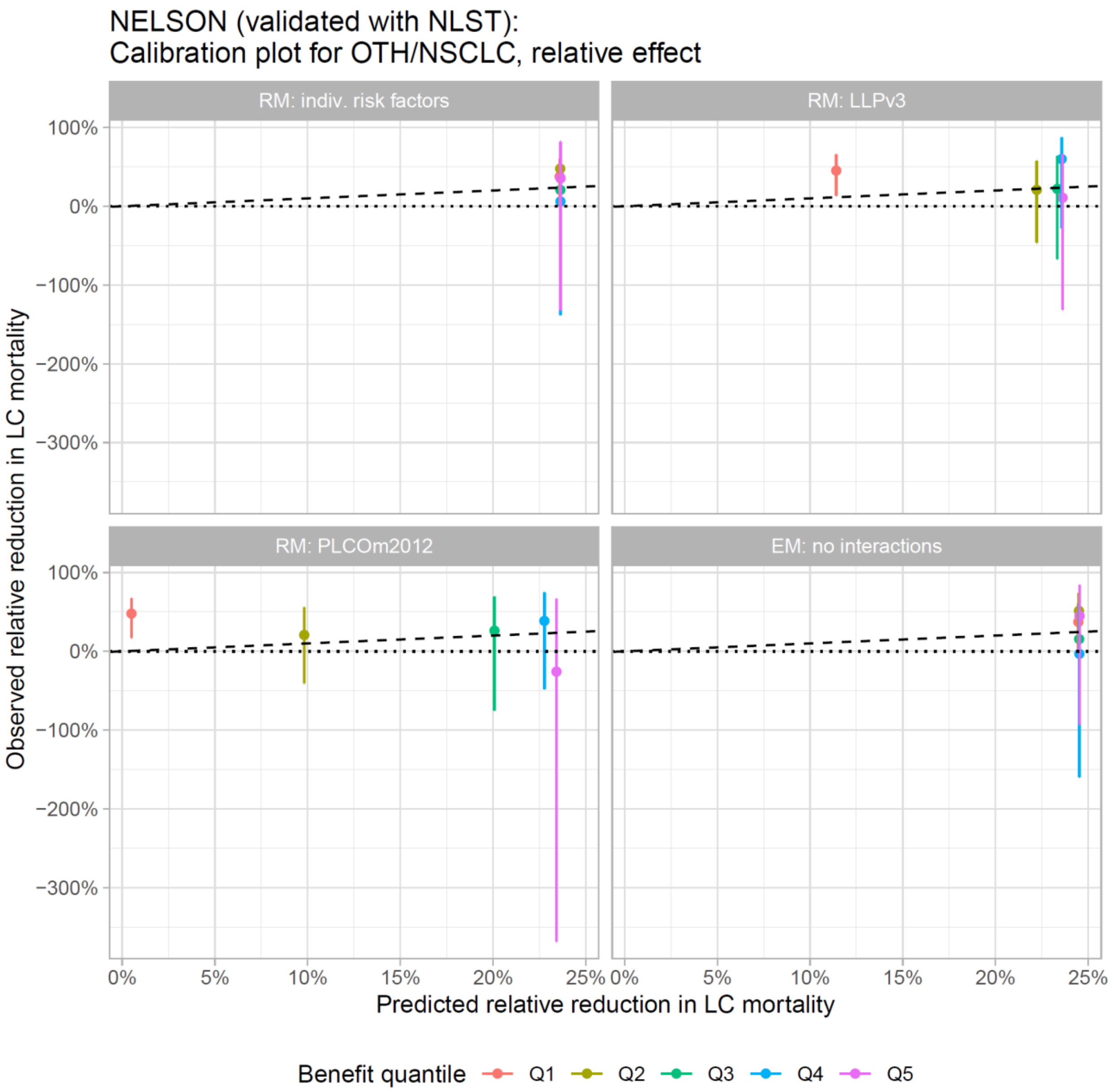

Figure notes: Based on N = 176 Other lung cancer deaths in NLST. The error bars represent the 95% confidence intervals.

Quintile thresholds	Q1	Q2	Q3	Q4	Q5
Risk modeling (individual risk-factors)	<23.61%	23.61% - 23.62%	23.62% - 23.63%	23.63% - 23.64%	≥23.64%
Risk modeling (LLPv3 model)	<20.60%	20.60% - 23.10%	23.10% - 23.50%	23.50% - 23.60%	≥23.60%
Risk modeling (PLCOm2012 model)	<2.66%	2.66% - 16.70%	16.70% - 22.10%	22.10% - 23.20%	≥23.20%
Effect modeling	<24.49%	24.49% - 24.51%	24.51% - 24.52%	24.52% - 24.53%	≥24.53%

Figure S58: Calibration for relative benefit (Others and non-small cell carcinoma not otherwise specified-specific mortality) for risk- and effect-models developed in NLST and validation in NELSON

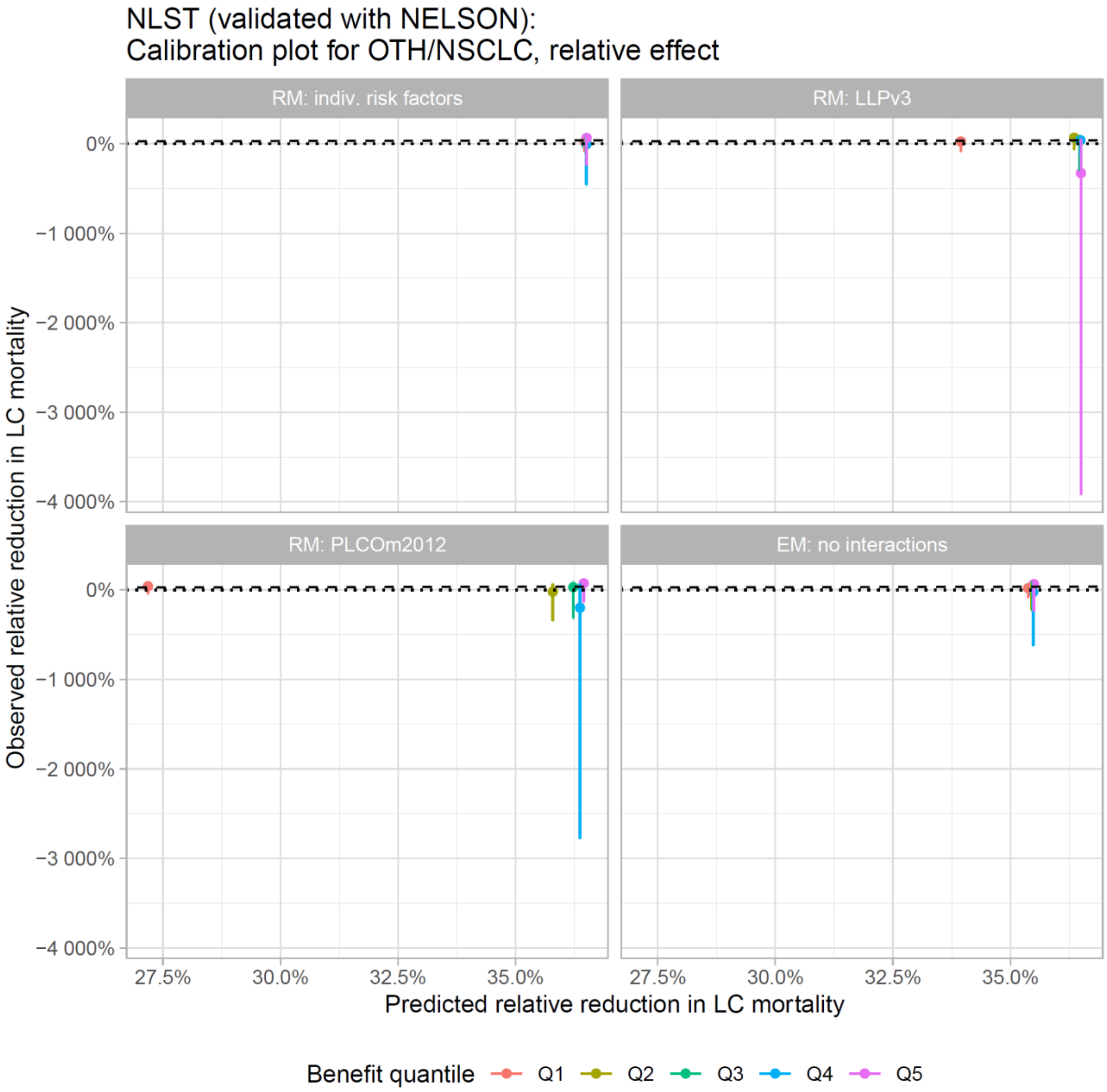

Figure notes: Based on N = 43 Other lung cancer deaths in NELSON. The error bars represent the 95% confidence intervals.

**Quintile thresholds**	**Q1**	**Q2**	**Q3**	**Q4**	**Q5**
**Risk modeling (individual risk-factors)**	<36.49%	36.49% - 36.50%	36.50% - 36.50%	36.50% - 36.51%	≥36.51%
**Risk modeling (LLPv3 model)**	<36.22%	36.22% - 36.44%	36.44% - 36.48%	36.48% - 36.49%	≥36.49%
**Risk modeling (PLCOm2012 model)**	<35.20%	35.20% - 36.10%	36.10% - 36.30%	36.30% - 36.40%	≥36.40%
**Effect modeling**	<35.43%	35.43% - 35.46%	35.46% - 35.48%	35.48% - 35.49%	≥35.49%

Figure S59: Calibration for relative benefit (Small cell carcinoma- specific mortality) for risk- and effect-models developed in NELSON and validation in NLST

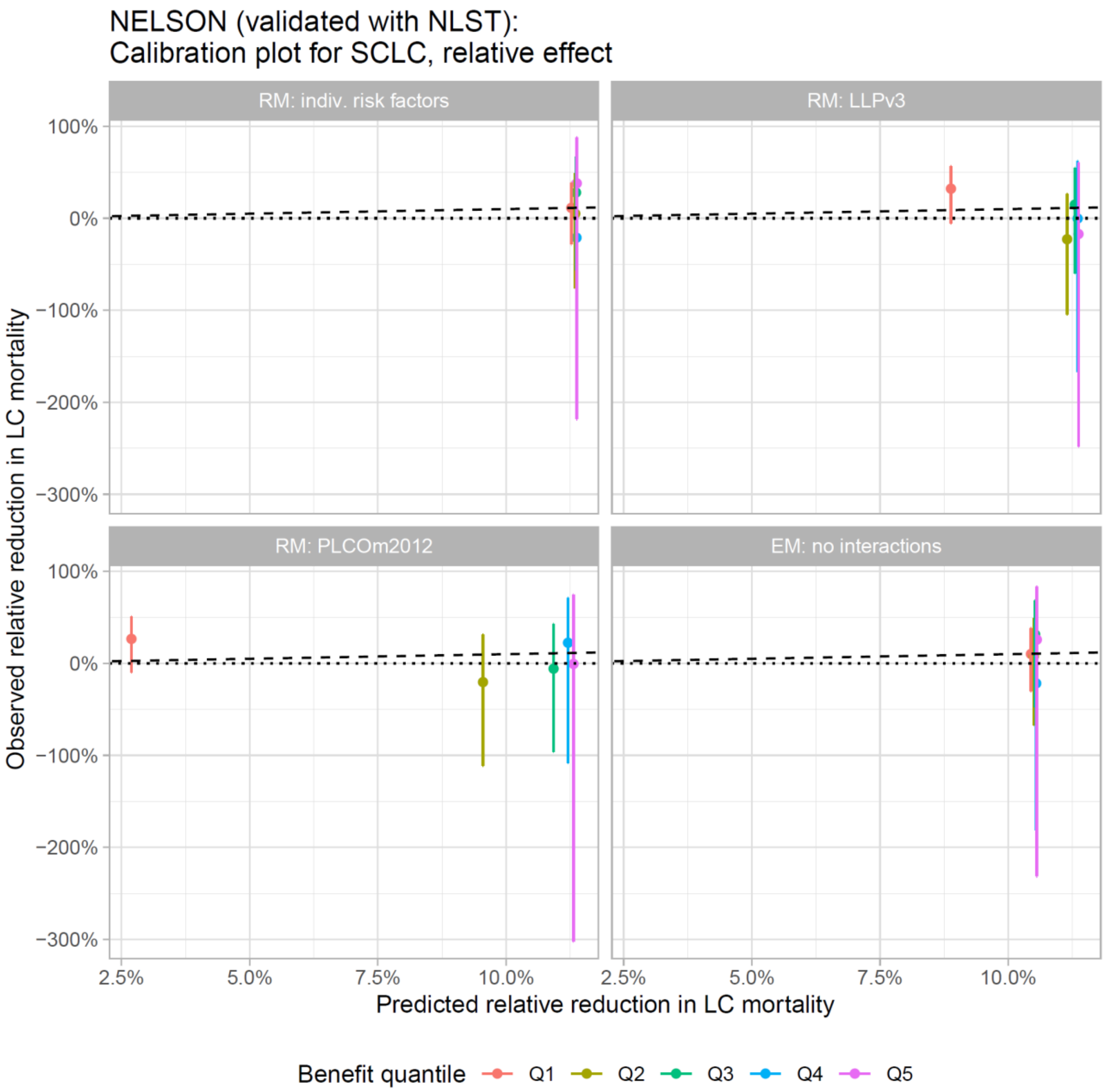

Figure notes: Based on N = 209 Small-cell carcinoma deaths in NLST. The error bars represent the 95% confidence intervals.

**Quintile thresholds**	**Q1**	**Q2**	**Q3**	**Q4**	**Q5**
**Risk modeling (individual risk-factors)**	<11.34%	11.34% - 11.36%	11.36% - 11.37%	11.37% - 11.38%	≥11.38%
**Risk modeling (LLPv3 model)**	<10.97%	10.97% - 11.26%	11.26% - 11.33%	11.33% - 11.36%	≥11.36%
**Risk modeling (PLCOm2012 model)**	<7.43%	7.43% - 10.60%	10.60% - 11.10%	11.10% - 11.30%	≥11.30%
**Effect modeling**	<10.48%	10.48% - 10.51%	10.51% - 10.54%	10.54% - 10.55%	≥10.55%

Figure S60: Calibration for relative benefit (Small cell carcinoma- specific mortality) for risk- and effect-models developed in NLST and validation in NELSON

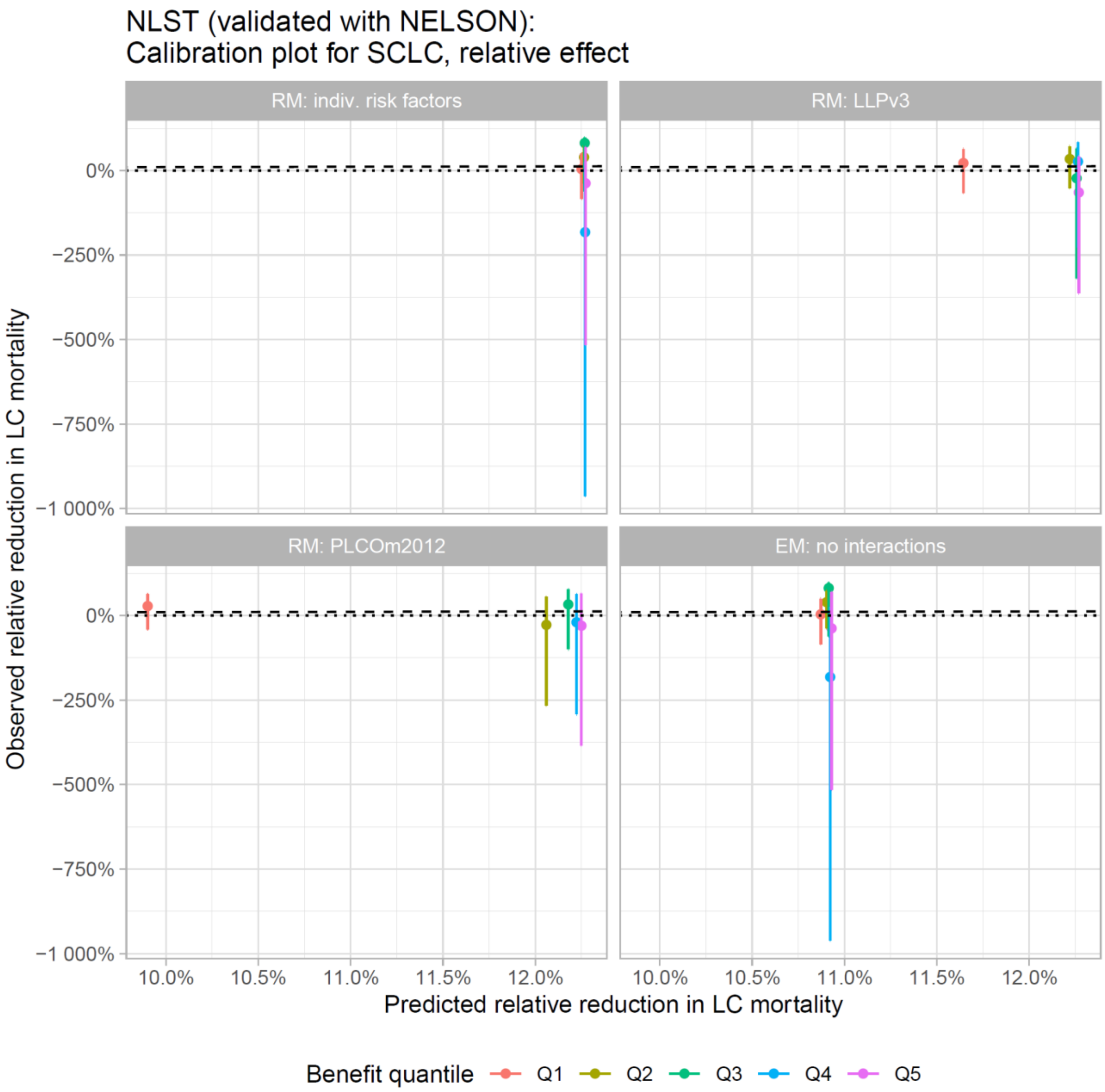

Figure notes: Based on N = 84 Small-cell carcinoma deaths in NELSON. The error bars represent the 95% confidence intervals.

**Quintile thresholds**	**Q1**	**Q2**	**Q3**	**Q4**	**Q5**
**Risk modeling (individual risk-factors)**	<12.26%	12.26% - 12.27%	12.27% - 12.27%	12.27% - 12.27%	≥12.27%
**Risk modeling (LLPv3 model)**	<12.18%	12.18% - 12.25%	12.25% - 12.26%	12.26% - 12.27%	≥12.27%
**Risk modeling (PLCOm2012 model)**	<11.92%	11.92% - 12.14%	12.14% - 12.21%	12.21% - 12.24%	≥12.24%
**Effect modeling**	<10.90%	10.90% - 10.91%	10.91% - 10.92%	10.92% - 10.93%	≥10.93%

Figure S61: Calibration for absolute benefit (overall LCM) for risk- and effect-models developed in NELSON and validation in NLST

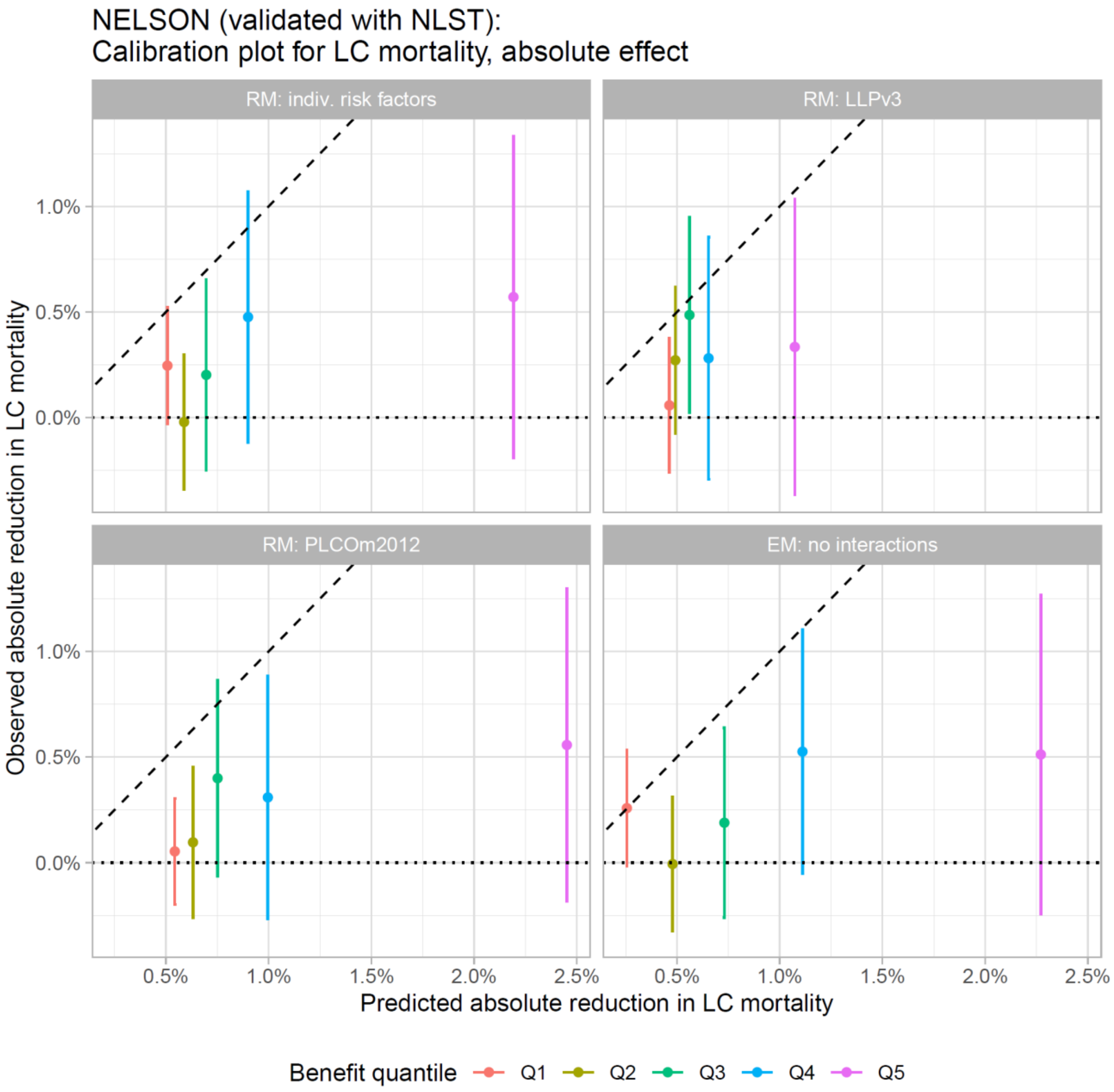

Figure notes: Based on N = 977 lung cancer deaths in NLST. The error bars represent the 95% confidence intervals.

**Quintile thresholds**	**Q1**	**Q2**	**Q3**	**Q4**	**Q5**
**Risk modeling (individual risk-factors)**	<0.55%	0.55% - 0.64%	0.64% - 0.77%	0.77% - 1.09%	≥1.09%
**Risk modeling (LLPv3 model)**	<0.47%	0.47% - 0.52%	0.52% - 0.60%	0.60% - 0.74%	≥0.74%
**Risk modeling (PLCOm2012 model)**	<0.59%	0.59% - 0.68%	0.68% - 0.84%	0.84% - 1.23%	≥1.23%
**Effect modeling**	<0.37%	0.37% - 0.60%	0.60% - 0.89%	0.89% - 1.41%	≥1.41%

Figure S62: Calibration for absolute benefit (overall LCM) for risk- and effect-models developed in NLST and validation in NELSON

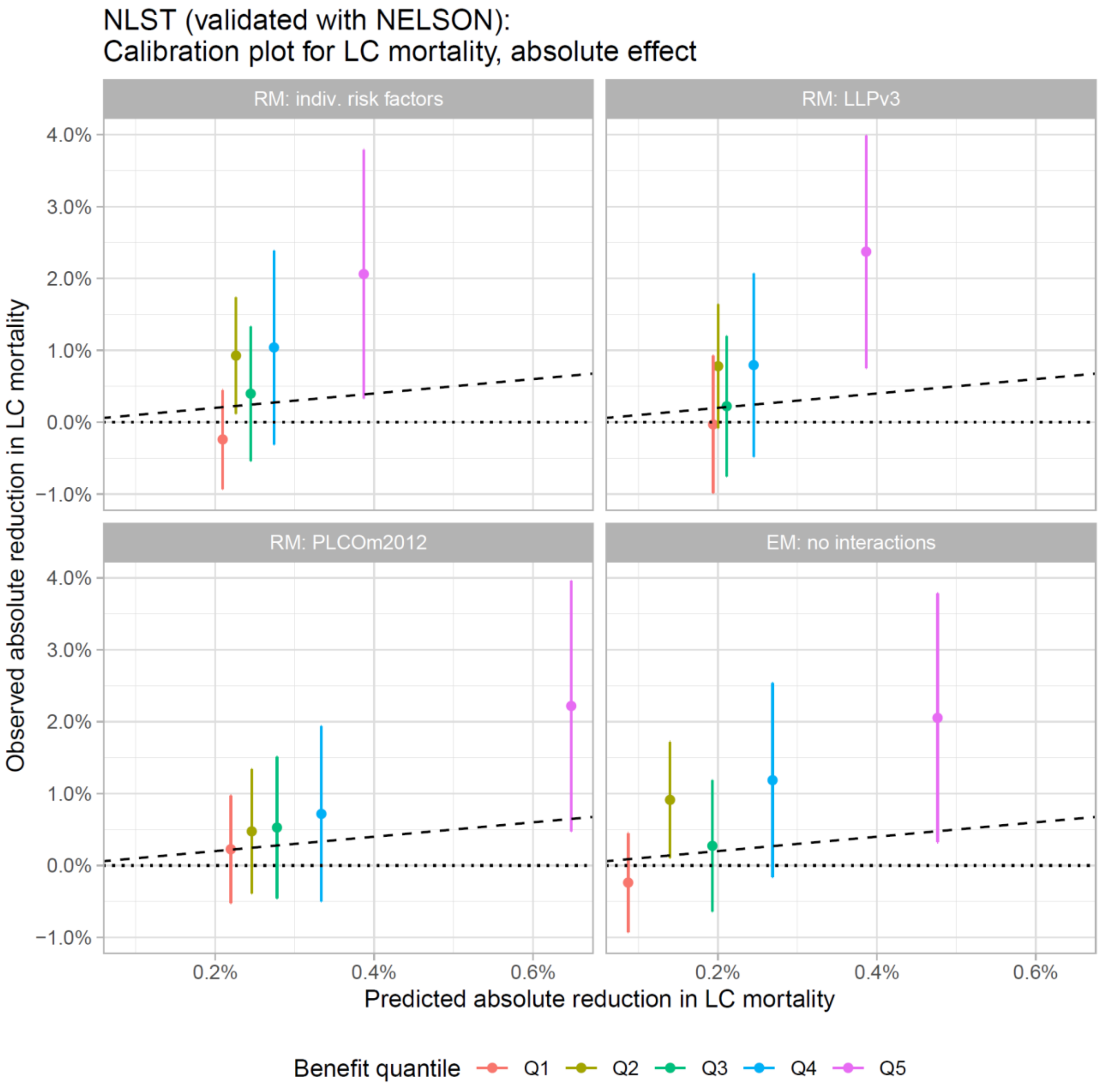

Figure notes: Based on N = 400 lung cancer deaths in NELSON. The error bars represent the 95% confidence intervals.

**Quintile thresholds**	**Q1**	**Q2**	**Q3**	**Q4**	**Q5**
**Risk modeling (individual risk-factors)**	<0.55%	0.55% - 0.64%	0.64% - 0.77%	0.77% - 1.09%	≥1.09%
**Risk modeling (LLPv3 model)**	<0.47%	0.47% - 0.52%	0.52% - 0.60%	0.60% - 0.74%	≥0.74%
**Risk modeling (PLCOm2012 model)**	<0.59%	0.59% - 0.68%	0.68% - 0.84%	0.84% - 1.23%	≥1.23%
**Effect modeling**	<0.37%	0.37% - 0.60%	0.60% - 0.89%	0.89% - 1.41%	≥1.41%

Figure S63: Calibration for absolute benefit (adenocarcinoma-specific mortality) for risk- and effect-models developed in NELSON and validation in NLST

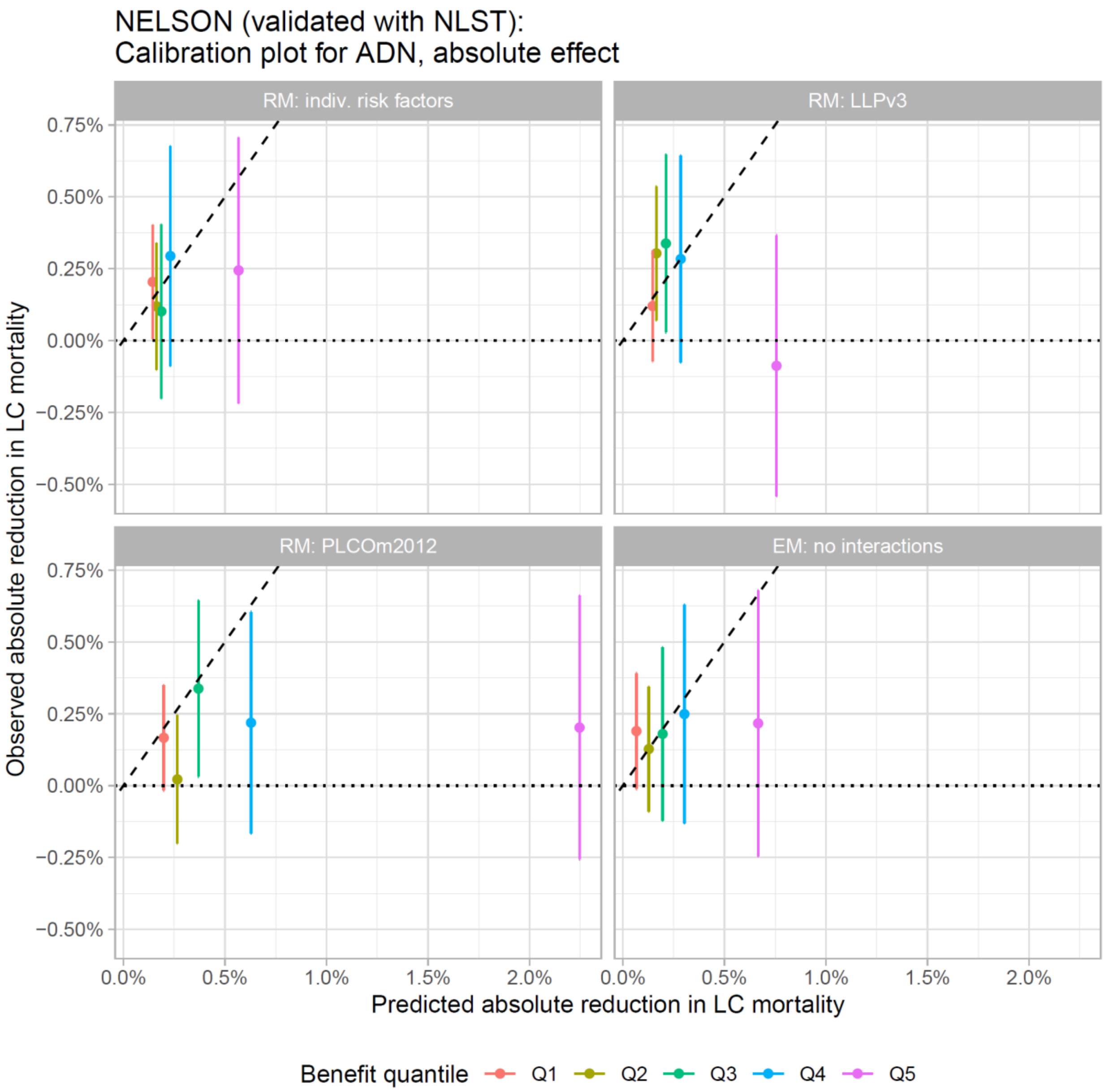

Figure notes: Based on N = 393 Adenocarcinoma deaths in NLST. The error bars represent the 95% confidence intervals.

Quintile thresholds	Q1	Q2	Q3	Q4	Q5
Risk modeling (individual risk-factors)	<0.15%	0.15% - 0.17%	0.17% - 0.20%	0.20% - 0.27%	≥0.27%
Risk modeling (LLPv3 model)	<0.15%	0.15% - 0.19%	0.19% - 0.24%	0.24% - 0.36%	≥0.36%
Risk modeling (PLCOm2012 model)	<0.23%	0.23% - 0.31%	0.31% - 0.45%	0.45% - 0.91%	≥0.91%
Effect modeling	<0.10%	0.10% - 0.16%	0.16% - 0.24%	0.24% - 0.39%	≥0.39%

Figure S64: Calibration for absolute benefit adenocarcinoma-specific mortality) for risk- and effect-models developed in NLST and validation in NELSON

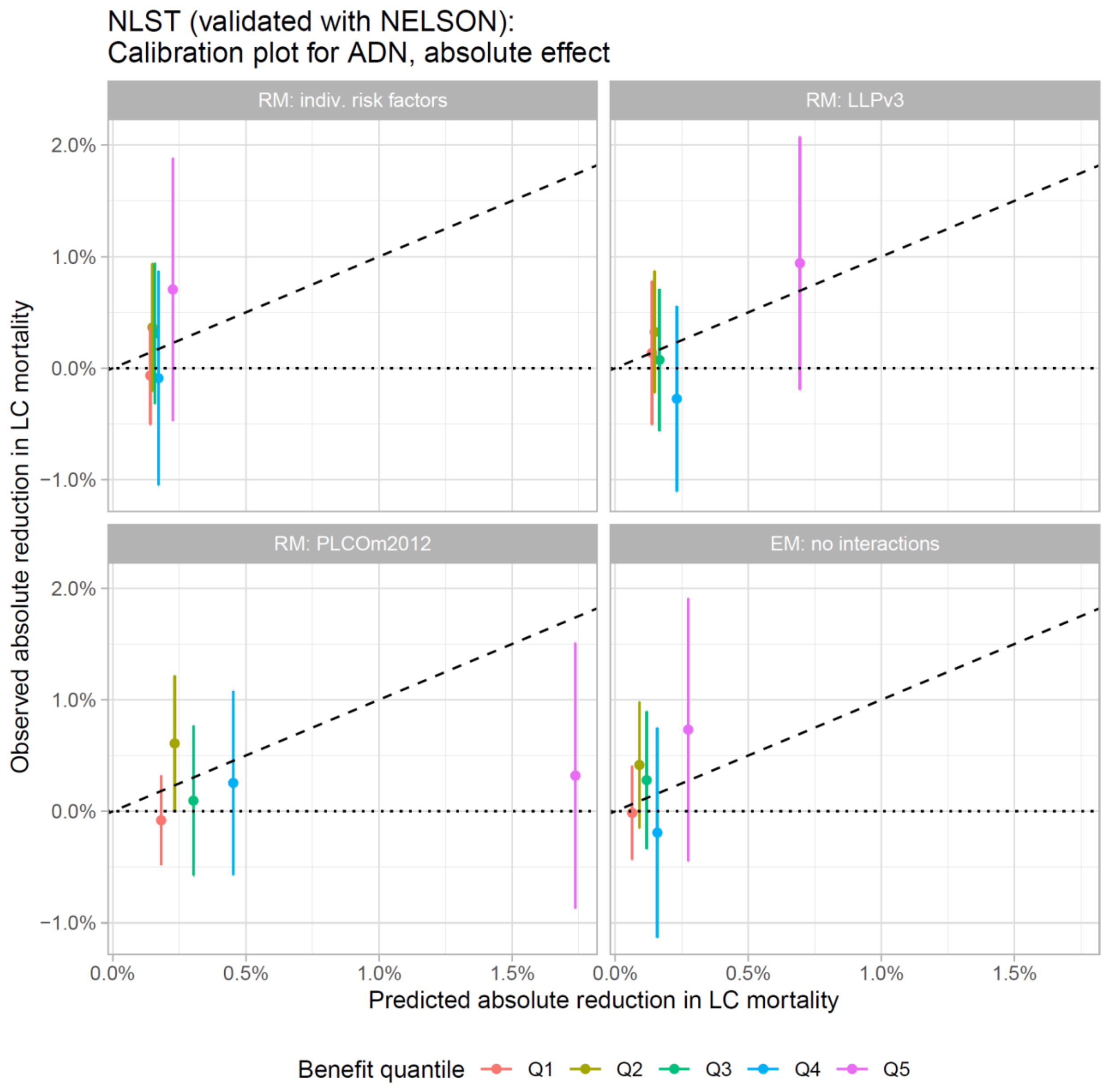

Figure notes: Based on N = 178 Adenocarcinoma deaths in NELSON. The error bars represent the 95% confidence intervals.

Quintile thresholds	Q1	Q2	Q3	Q4	Q5
**Risk modeling (individual risk-factors)**	<0.14%	0.14% - 0.15%	0.15% - 0.16%	0.16% - 0.18%	≥0.18%
**Risk modeling (LLPv3 model)**	<0.14%	0.14% - 0.16%	0.16% - 0.18%	0.18% - 0.31%	≥0.31%
**Risk modeling (PLCOm2012 model)**	<0.21%	0.21% - 0.26%	0.26% - 0.36%	0.36% - 0.60%	≥0.60%
**Effect modeling**	<0.08%	0.08% - 0.10%	0.10% - 0.14%	0.14% - 0.19%	≥0.19%

Figure S65: Calibration for absolute benefit (squamous cell carcinoma-specific mortality) for risk- and effect-models developed in NELSON and validation in NLST

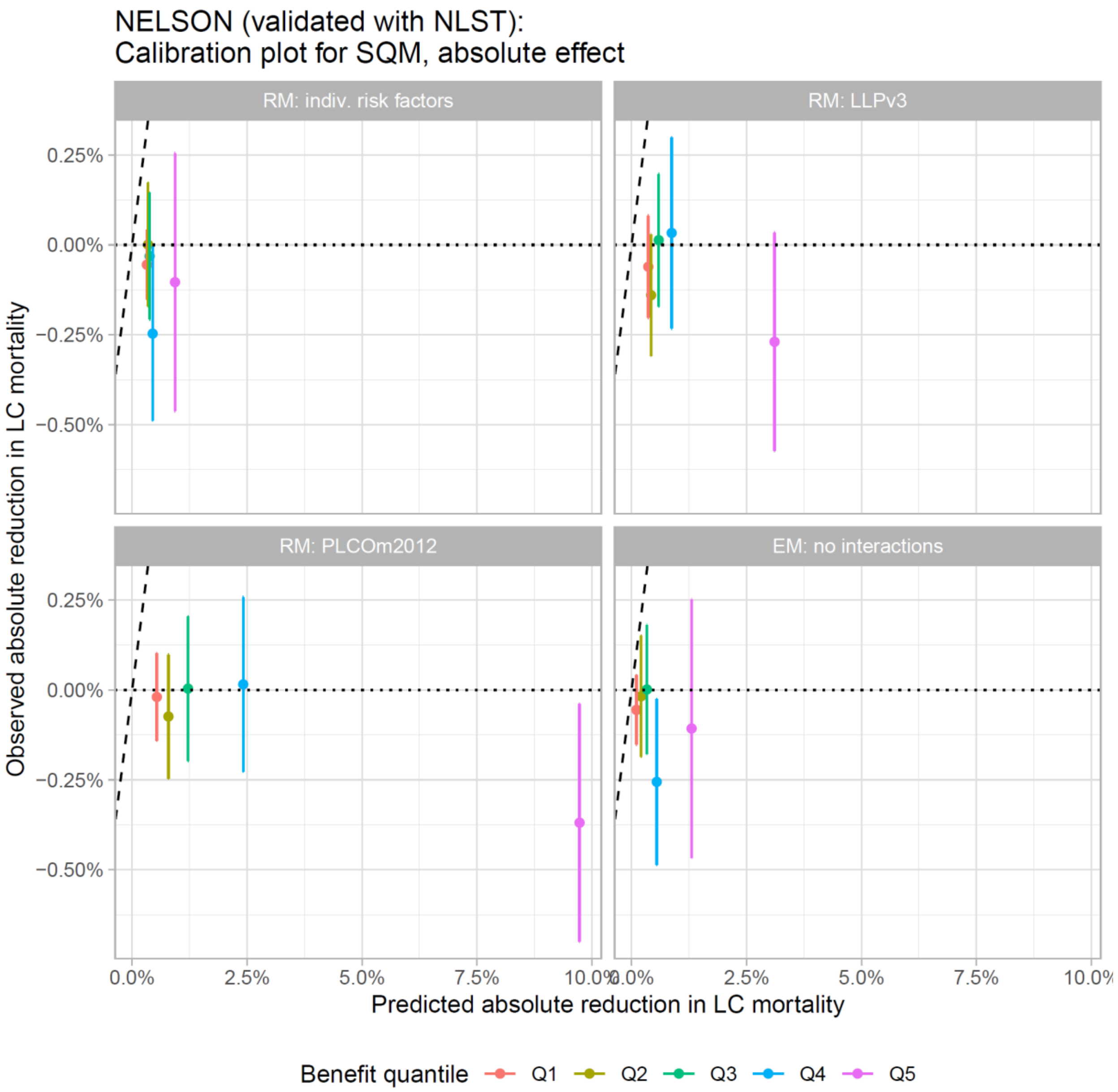

Figure notes: Based on N = 184 Squamous-cell carcinoma deaths in NLST. The error bars represent the 95% confidence intervals.

Quintile thresholds	Q1	Q2	Q3	Q4	Q5
Risk modeling (individual risk-factors)	<0.33%	0.33% - 0.36%	0.36% - 0.40%	0.40% - 0.50%	≥0.50%
Risk modeling (LLPv3 model)	<0.39%	0.39% - 0.49%	0.49% - 0.69%	0.69% - 1.19%	≥1.19%
Risk modeling (PLCOm2012 model)	<0.65%	0.65% - 0.95%	0.95% - 1.58%	1.58% - 3.81%	≥3.81%
Effect modeling	<0.16%	0.16% - 0.26%	0.26% - 0.42%	0.42% - 0.72%	≥0.72%

Figure S66: Calibration for absolute benefit squamous cell carcinoma-specific mortality) for risk- and effect-models developed in NLST and validation in NELSON

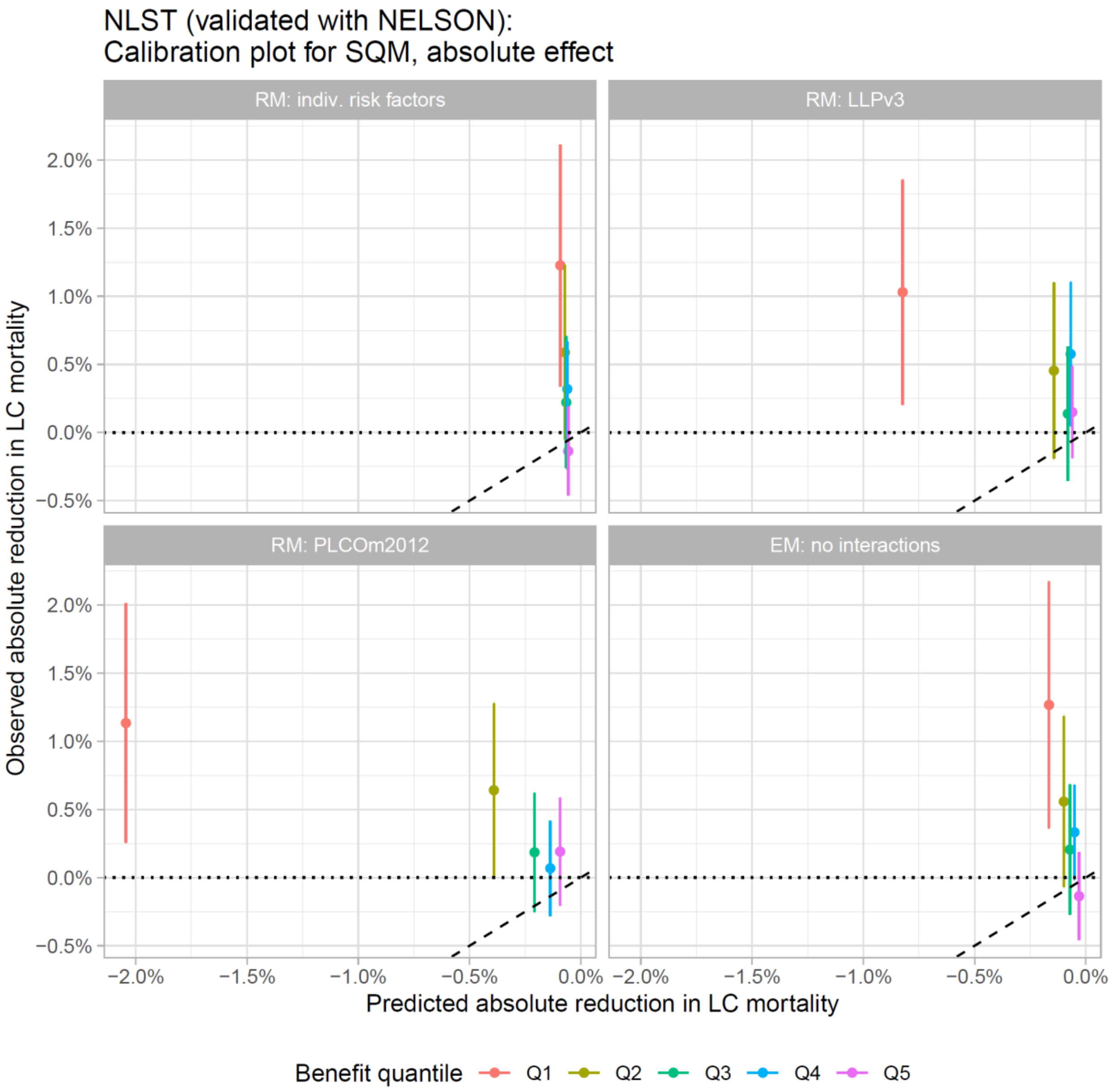

Figure notes: Based on N = 94 Squamous-cell carcinoma deaths in NELSON. The error bars represent the 95% confidence intervals.

Quintile thresholds	Q1	Q2	Q3	Q4	Q5
**Risk modeling (individual risk-factors)**	<-0.08%	-0.08% - -0.07%	-0.07% - -0.06%	-0.06% - -0.06%	≥-0.06%
**Risk modeling (LLPv3 model)**	<-0.21%	-0.21% - -0.09%	-0.09% - -0.07%	-0.07% - -0.06%	≥-0.06%
**Risk modeling (PLCOm2012 model)**	<-0.59%	-0.59% - -0.27%	-0.27% - -0.16%	-0.16% - -0.11%	≥-0.11%
**Effect modeling**	<-0.12%	-0.12% - -0.08%	-0.08% - -0.06%	-0.06% - -0.04%	≥-0.04%

Figure S67: Calibration for absolute benefit (Others and non-small cell carcinoma not otherwise specified-specific mortality) for risk- and effect-models developed in NELSON and validation in NLST

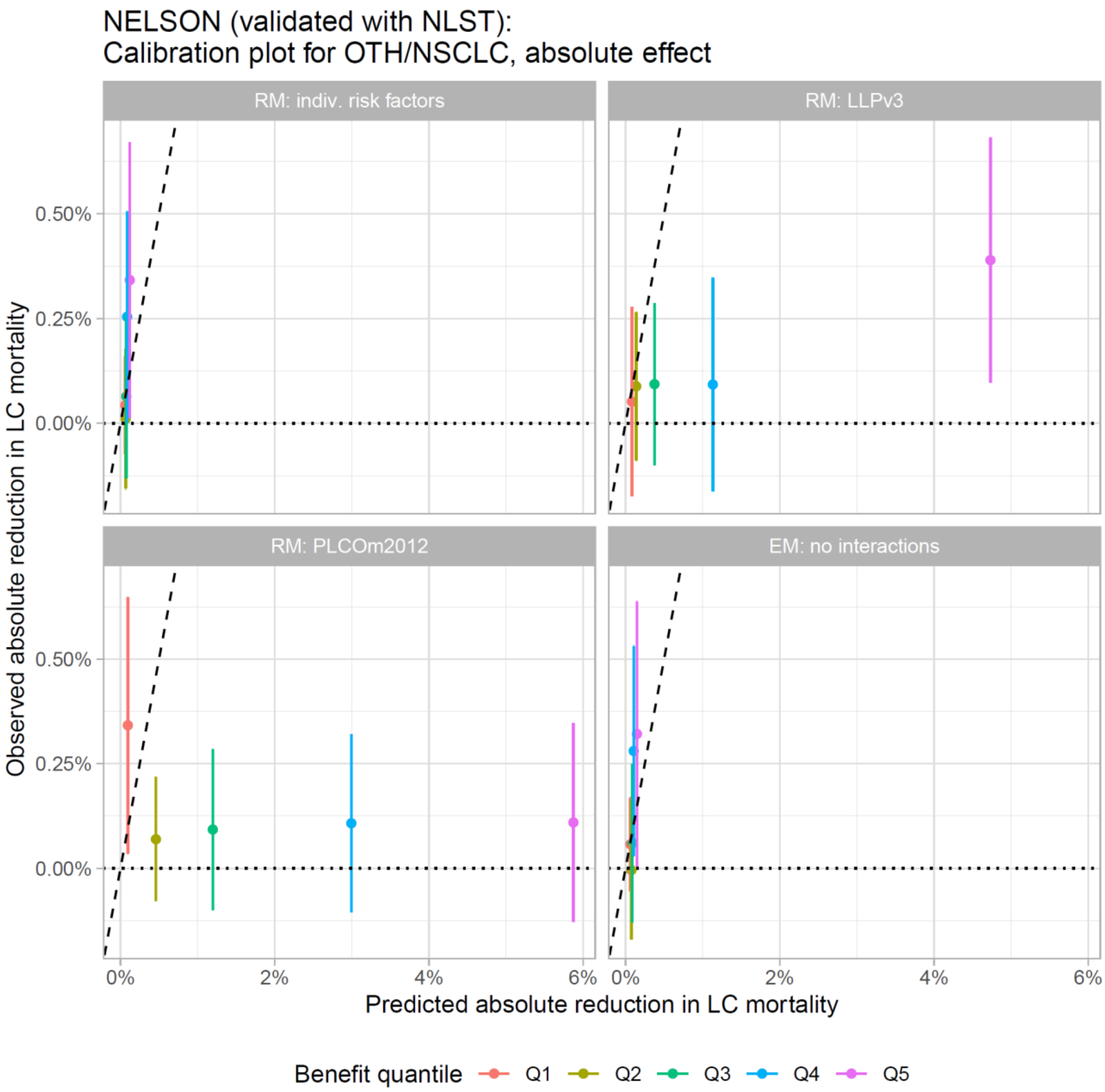

Figure notes: Based on N = 176 Other lung cancer deaths in NLST. The error bars represent the 95% confidence intervals.

Quintile thresholds	Q1	Q2	Q3	Q4	Q5
Risk modeling (individual risk-factors)	<0.07%	0.07% - 0.07%	0.07% - 0.08%	0.08% - 0.09%	≥0.09%
Risk modeling (LLPv3 model)	<0.10%	0.10% - 0.21%	0.21% - 0.61%	0.61% - 2.40%	≥2.40%
Risk modeling (PLCOm2012 model)	<0.29%	0.29% - 0.79%	0.79% - 2.01%	2.01% - 4.45%	≥4.45%
Effect modeling	<0.07%	0.07% - 0.08%	0.08% - 0.10%	0.10% - 0.12%	≥0.12%

Figure S68: Calibration for absolute benefit (Others and non-small cell carcinoma not otherwise specified-specific mortality) for risk- and effect-models developed in NLST and validation in NELSON

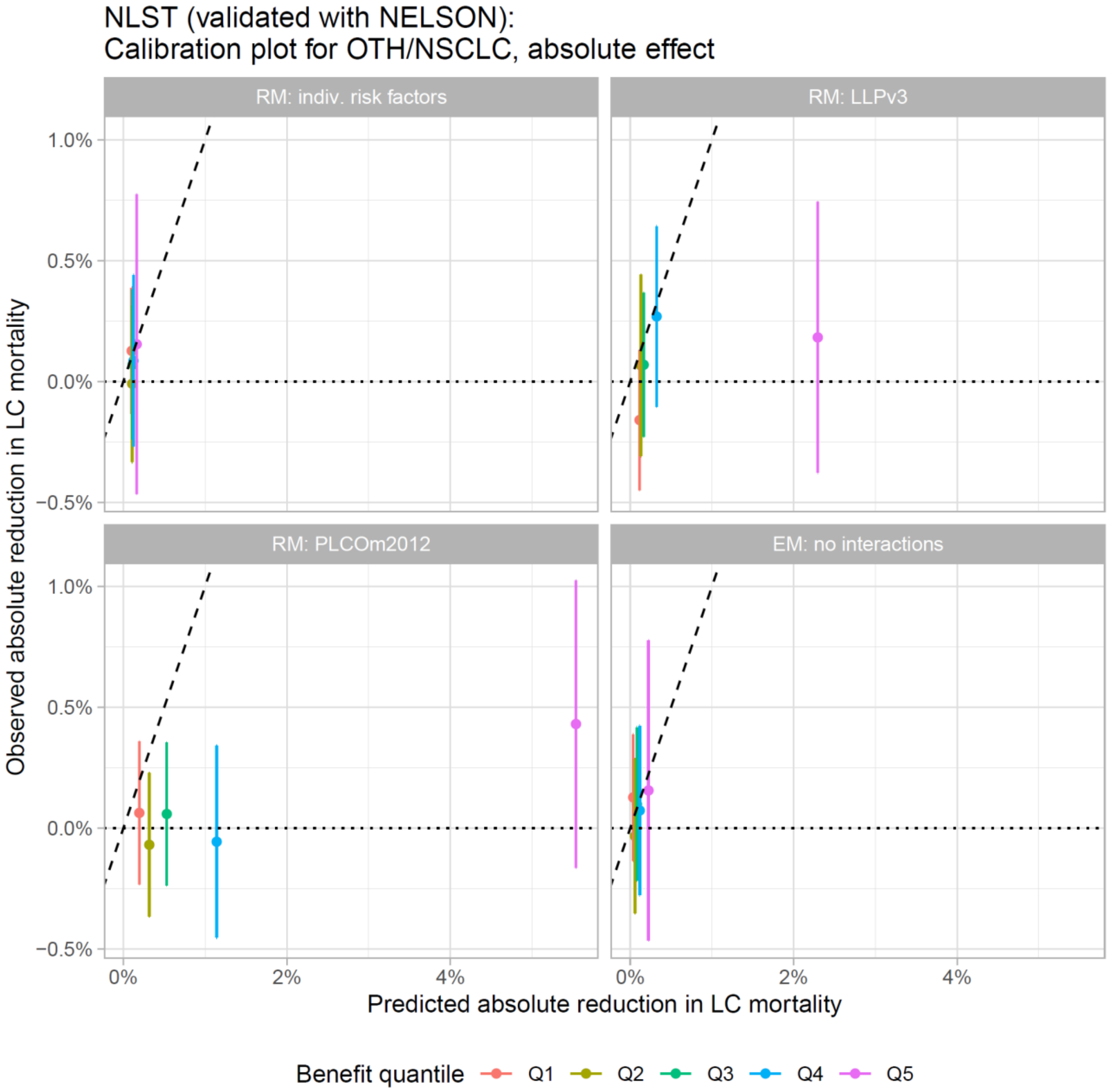

Figure notes: Based on N = 43 Other lung cancer deaths in NELSON. The error bars represent the 95% confidence intervals.

Quintile thresholds	Q1	Q2	Q3	Q4	Q5
Risk modeling (individual risk-factors)	<0.10%	0.10% - 0.11%	0.11% - 0.12%	0.12% - 0.13%	≥0.13%
Risk modeling (LLPv3 model)	<0.12%	0.12% - 0.14%	0.14% - 0.20%	0.20% - 0.55%	≥0.55%
Risk modeling (PLCOm2012 model)	<0.25%	0.25% - 0.40%	0.40% - 0.72%	0.72% - 1.85%	≥1.85%
Effect modeling	<0.05%	0.05% - 0.07%	0.07% - 0.10%	0.10% - 0.14%	≥0.14%

Figure S69: Calibration for absolute benefit (Small cell carcinoma- specific mortality) for risk- and effect-models developed in NELSON and validation in NLST

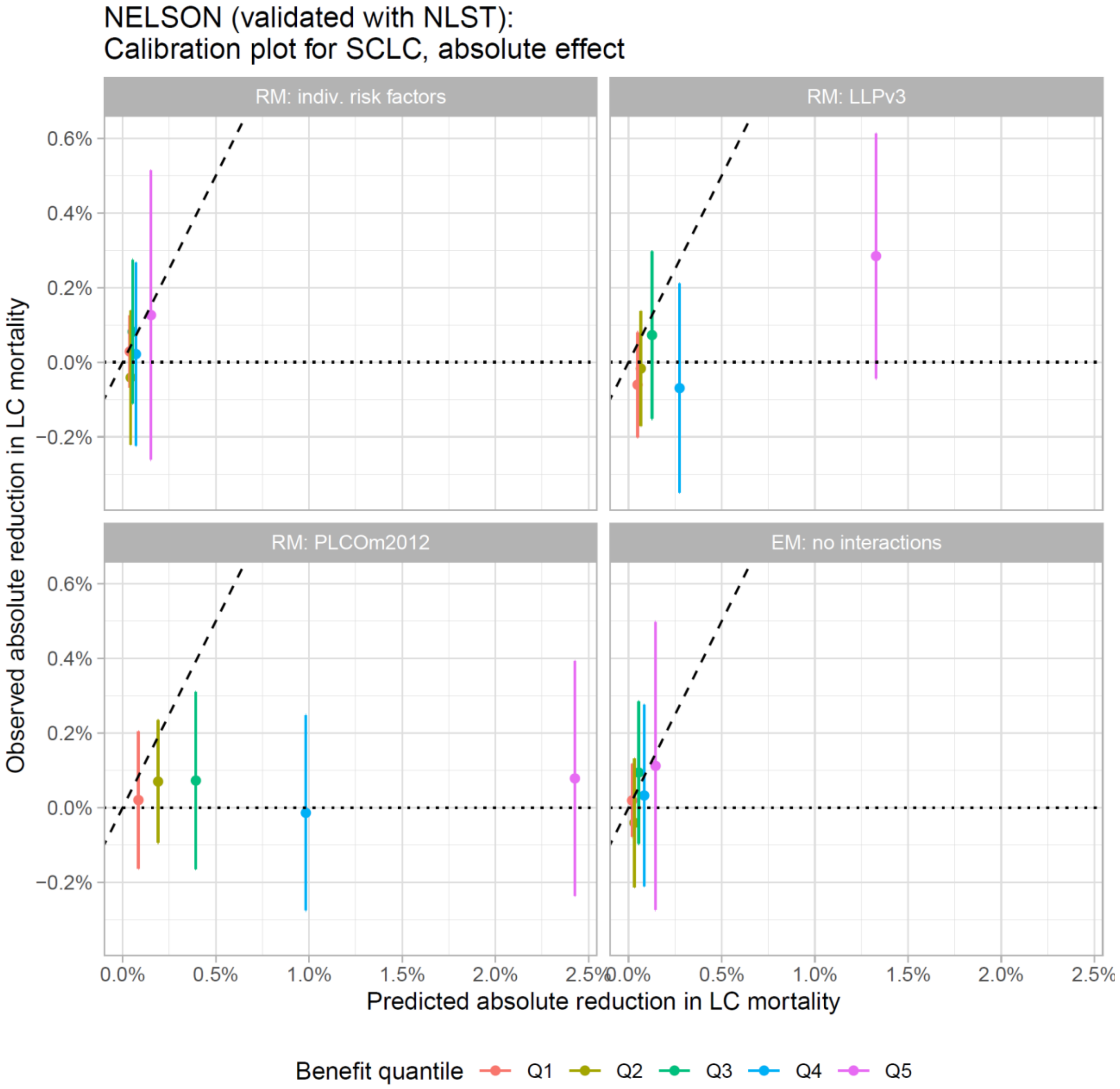

Figure notes: Based on N = 209 Small-cell carcinoma deaths in NLST. The error bars represent the 95% confidence intervals.

Quintile thresholds	Q1	Q2	Q3	Q4	Q5
Risk modeling (individual risk-factors)	<0.04%	0.04% - 0.05%	0.05% - 0.06%	0.06% - 0.09%	≥0.09%
Risk modeling (LLPv3 model)	<0.06%	0.06% - 0.09%	0.09% - 0.17%	0.17% - 0.48%	≥0.48%
Risk modeling (PLCOm2012 model)	<0.13%	0.13% - 0.26%	0.26% - 0.58%	0.58% - 1.58%	≥1.58%
Effect modeling	<0.02%	0.02% - 0.04%	0.04% - 0.07%	0.07% - 0.10%	≥0.10%

Figure S70: Calibration for absolute benefit (Small cell carcinoma- specific mortality) for risk- and effect-models developed in NLST and validation in NELSON

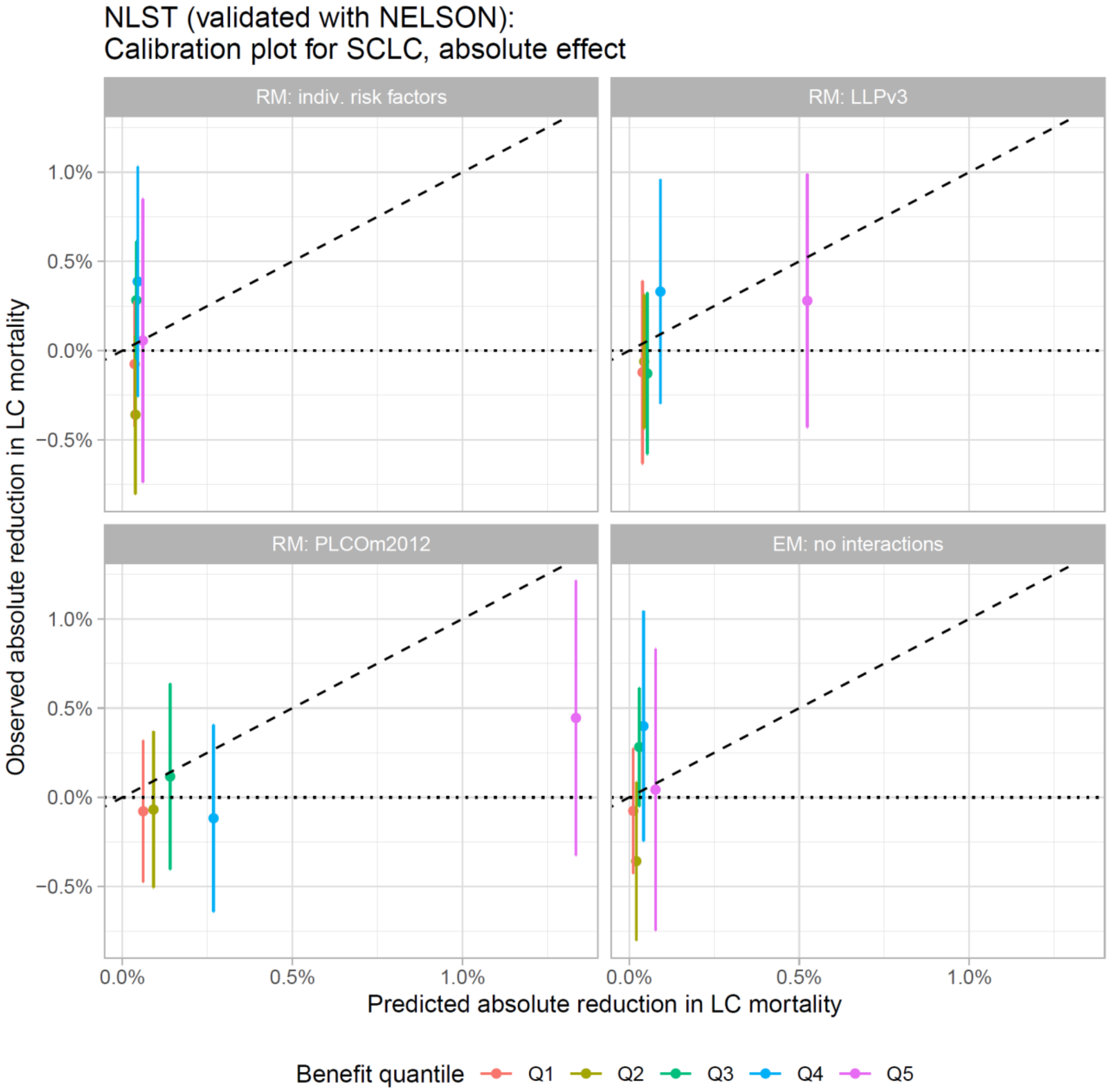

Figure notes: Based on N = 84 Small-cell carcinoma deaths in NELSON. The error bars represent the 95% confidence intervals.

Quintile thresholds	Q1	Q2	Q3	Q4	Q5
Risk modeling (individual risk-factors)	<0.04%	0.04% - 0.04%	0.04% - 0.04%	0.04% - 0.05%	≥0.05%
Risk modeling (LLPv3 model)	<0.04%	0.04% - 0.05%	0.05% - 0.06%	0.06% - 0.14%	≥0.14%
Risk modeling (PLCOm2012 model)	<0.07%	0.07% - 0.11%	0.11% - 0.18%	0.18% - 0.41%	≥0.41%
Effect modeling	<0.02%	0.02% - 0.02%	0.02% - 0.03%	0.03% - 0.05%	≥0.05%

Pooled analysis

As a sensitivity analysis, we perform a separate analysis in which we pool the individual patient-level data from NELSON and NLST into one joint dataset. The datasets were pooled based on the variables that were measured in both trials. The variables dropped were information on race and family history of lung cancer since they were only measured in NLST, but not NELSON.

The idea behind pooling the data from NELSON and NLST is to explicitly capture pre-screening design differences between these two trials, such as differences in eligibility criteria and trial population. To account for such differences, we design a multilevel model based on propensity score matching, based on recent recommendations by Chang et al.[16] Note that this model may not be compliant with the PATH statement because multilevel matching models are not discussed therein.[17] Therefore, this analysis might be viewed as sensitivity analysis that, unlike the other comparative models, explicitly adjusts for pre-screen design differences in trial design. The model is described in detail in the Statistical Methodology Supplementary Appendix. All results are adjusted for imputation uncertainty from 30 imputation runs following Rubin's rules.

In agreement with Chang et al, the multilevel propensity model comprises two stages.[16] In the first stage, the probability of being screened by either protocol (that is, the propensity score) is estimated for each patient using a logistic linear regression model with a trial dummy variable and trial-specific coefficients. Then, in a second stage, each screened patient is matched to a non-screened patient from the same trial based on the first stage propensity scores. Then, using the ensuing matched dataset, screening effectiveness is estimated for either trial by using weights constructed with the propensity scores from the first stage.

Figure S71 shows a histogram of the estimated propensity scores for each of the 68,213 participants in the pooled data. The average propensity scores amounts to 0.4999 (median=0.5006, Interquartile Range = 0.005187) and is centered about 0.5 (which indicates random screening assignment). Supplementary data 4 shows the coefficient estimates of the propensity score model. Since the propensity scores are narrowly centered about 0.5 and no covariate is significant at the 5% level save for: 1) COPD in NELSON, which had a 35% rate of missing data due to not being asked during the first recruitment round; 2) personal history of cancer in NLST, which had an uneven distribution between the screen and control group (Supplementary data 1), particularly the trial fixed effects, we conclude that the propensity model accounts for all differences in pre-screening characteristics between trials.

Figure S72 shows the estimated treatment effects for each histology, based on the second stage risk-model. The point estimates are very similar to those from the models in the main text; see Figure 3 in the main text. Notably, the confidence intervals are wider, which is expected because

estimates in propensity score matching are based on smaller datasets, namely the trial-specific datasets for which matches could be found instead of the full dataset.

We conclude that a pooled model that explicitly accounts for pre-screening differences between the two trials produces nearly equivalent results than the models from the main text. It follows that there is no evidence that there are unaccounted between-trial differences in the models from the main text.

Figure S71: Histogram of estimated propensity scores in the pooled dataset.

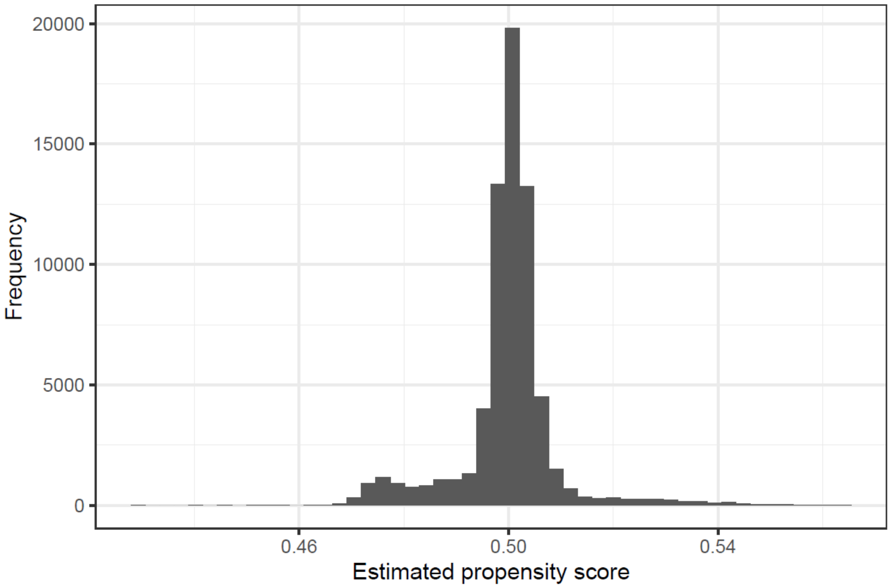

Figure notes: The figure was based on N = 68,213 individuals (N = 14,808 individuals from NELSON and N = 53,405 individuals from NLST).

Figure S72: Estimated screening effectiveness for overall lung cancer mortality and by histology-specific mortality

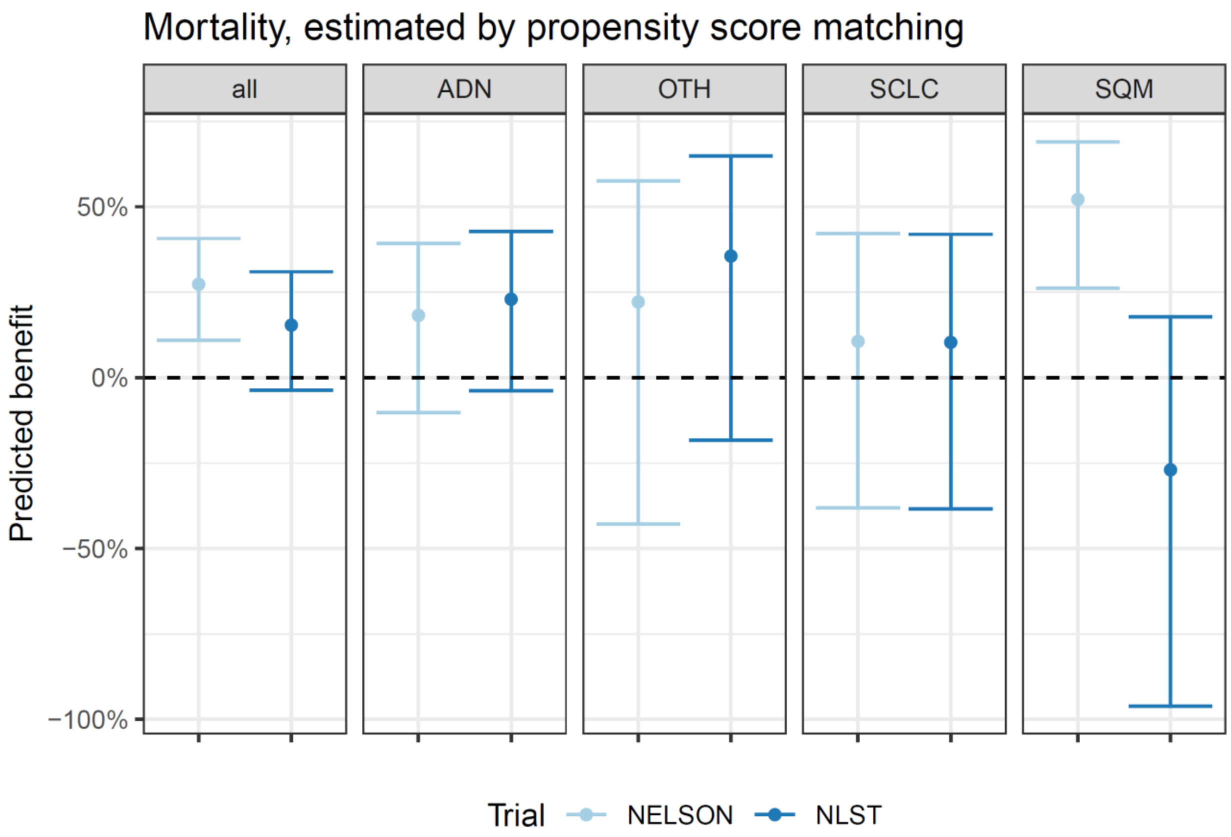

Figure notes: Estimates were constructed using the multilevel propensity score matching model on the pooled dataset. Error bars represent 95% confidence intervals. Based on N = 400 overall lung cancer deaths, N = 178 Adenocarcinoma deaths, N = 94 Squamous-cell carcinoma deaths, N = 43 Other lung cancer deaths and N = 84 Small-cell carcinoma deaths in NELSON and N = 977 overall lung cancer deaths N = 393 Adenocarcinoma deaths, N = 184 Squamous-cell carcinoma deaths, N = 176 Other lung cancer deaths and N = 209 Small-cell carcinoma deaths in NLST.

References

1 Athey, S., Tibshirani, J. & Wager, S. Generalized random forests. *The Annals of Statistics* **47**, 1148-1178 (2019). https://doi.org/10.1214/18-aos1709

2 Kent, D. M. *et al*. The Predictive Approaches to Treatment effect Heterogeneity (PATH) Statement. *Annals of Internal Medicine*

172, 35-45 (2020).

3 Lehmann, E. L. and Romano, J. P. (1986). Testing Statistical Hypotheses.

Wadsworth & Brooks/Cole, Pacific Grove, California, 2nd edition.

4 Zou, H. & Hastie, T. Regularization and variable selection via the elastic net. *Journal of the Royal Statistical Society: Series B (Statistical Methodology)* **67**, 301-320 (2005). https://doi.org/https://doi.org/10.1111/j.1467-9868.2005.00503.x

5 Friedman, J., Hastie, T. & Tibshirani, R. Regularization Paths for Generalized Linear Models via Coordinate Descent. *J Stat Softw* **33**, 1-22 (2010).

6 Tammemägi, M. C. *et al*. Selection Criteria for Lung-Cancer Screening. *New England Journal of Medicine* **368**, 728-736 (2013). https://doi.org/10.1056/NEJMoa1211776

7 Van der Vaart, A. W. (1998). Asymptotic Statistics. Cambridge Series in Statistical

and Probabilistic Mathematics. Cambridge University Press, Cambridge, UK.

8 Athey, S. and Wager, S. (2019). Estimating treatment effects with causal forests:

An application. arXiv preprint: arXiv1902.07409.

9 Alli, B. Y. InteractionR: An R package for full reporting of effect modification and interaction. *Software Impacts* **10**, 100147 (2021). https://doi.org/https://doi.org/10.1016/j.simpa.2021.100147

10 Hosmer, D. W. & Lemeshow, S. Confidence Interval Estimation of Interaction. *Epidemiology* **3** (1992).

11 European Commission: CORDIS EU research results 4-IN THE LUNG RUN: towards INdividually tailored INvitations, screening INtervals, and INtegrated co-morbidity reducing strategies in lung cancer screening. https://cordis.europa.eu/project/id/848294 Date accessed: January 4, 2022.

12 Oudkerk, M. *et al*. European position statement on lung cancer screening. *Lancet Oncol* **18**, e754-e766 (2017).

13 Crosbie, P. A. *et al*. Yorkshire Lung Screening Trial (YLST): protocol for a randomised controlled trial to evaluate invitation to community-based low-dose CT screening for lung cancer versus usual care in a targeted population at risk. *BMJ Open* **10**, e037075 (2020).

14 Fritz A, Percy C, Jack A, Shanmugaratnam K, Sobin L, Parkin DM, et al.. International classification of diseases for oncology. 3rd ed. Geneva: World Health Organization; 2000.

15 Field, J. K., Vulkan, D., Davies, M. P. A., Duffy, S. W. & Gabe, R. Liverpool Lung Project lung cancer risk stratification model: calibration and prospective validation. *Thorax* **76**, 161-168 (2021).

16 Chang, T.-H. & Stuart, E. A. Propensity score methods for observational studies with clustered data: A review. *Statistics in Medicine* **41**, 3612-3626 (2022). https://doi.org/https://doi.org/10.1002/sim.9437
17 Kent, D. M. *et al*. The Predictive Approaches to Treatment effect Heterogeneity (PATH) Statement: Explanation and Elaboration. *Annals of Internal Medicine* **172**, W1-W25 (2019). https://doi.org/10.7326/m18-3668
