# Supplementary material for: A comparative analysis of heterogeneity in lung cancer screening effectiveness in two randomised controlled trials
Source: Nat Commun. 2025 Aug 28;16:8060. doi: 10.1038/s41467-025-63471-6 (PMC12394595; doi:10.1038/s41467-025-63471-6)
Supplement: Supplementary file 2 — Description of Additional Supplementary Files [file 41467_2025_63471_MOESM2_ESM.pdf]

Description of Additional Supplementary Files

Supplementary Data 1- Baseline Characteristics of NELSON and NLST Participants by lung cancer mortality.

Supplementary Data 2 - Parameter estimates for the second stage of the risk-modelling approach for histology-specific mortality in NELSON

Supplementary Data 3- Parameter estimates for the second stage of the risk-modelling approach by histology in NLST

Supplementary Data 4- Coefficients of the first stage propensity score logistic regression model with screening arm assignment as binary outcome variable.
