# Supplementary material for: A comparative analysis of heterogeneity in lung cancer screening effectiveness in two randomised controlled trials
Source: Nat Commun. 2025 Aug 28;16:8060. doi: 10.1038/s41467-025-63471-6 (PMC12394595; doi:10.1038/s41467-025-63471-6)
Supplement: Supplementary file 7 — Reporting Summary [file 41467_2025_63471_MOESM7_ESM.pdf]

nature portfolio

Corresponding author(s): Kevin ten Haaf, Max Welz

Last updated by author(s): Apr 25, 2025

Reporting Summary

Nature Portfolio wishes to improve the reproducibility of the work that we publish. This form provides structure for consistency and transparency in reporting. For further information on Nature Portfolio policies, see our Editorial Policies and the Editorial Policy Checklist.

Statistics

For all statistical analyses, confirm that the following items are present in the figure legend, table legend, main text, or Methods section.

n/a	Confirmed	
☐	☒	The exact sample size (*n*) for each experimental group/condition, given as a discrete number and unit of measurement
☒	☐	A statement on whether measurements were taken from distinct samples or whether the same sample was measured repeatedly
☐	☒	The statistical test(s) used AND whether they are one- or two-sided<br>*Only common tests should be described solely by name; describe more complex techniques in the Methods section.*
☐	☒	A description of all covariates tested
☐	☒	A description of any assumptions or corrections, such as tests of normality and adjustment for multiple comparisons
☐	☒	A full description of the statistical parameters including central tendency (e.g. means) or other basic estimates (e.g. regression coefficient) AND variation (e.g. standard deviation) or associated estimates of uncertainty (e.g. confidence intervals)
☐	☒	For null hypothesis testing, the test statistic (e.g. *F*, *t*, *r*) with confidence intervals, effect sizes, degrees of freedom and *P* value noted<br>*Give P values as exact values whenever suitable.*
☒	☐	For Bayesian analysis, information on the choice of priors and Markov chain Monte Carlo settings
☒	☐	For hierarchical and complex designs, identification of the appropriate level for tests and full reporting of outcomes
☐	☒	Estimates of effect sizes (e.g. Cohen's *d*, Pearson's *r*), indicating how they were calculated

Our web collection on statistics for biologists contains articles on many of the points above.

Software and code

Policy information about availability of computer code

Data collection	No software was used in data collection
Data analysis	R version 4.2.0 was used for all analyses. We used our customly developed R-package Predmod (version 0.0.1; https://doi.org/10.5281/zenodo.15065276) for the analyses, which can be freely accessed at: Welz M., ten Haaf K., Alfons A. predmod: Predictive modeling for heterogeneous treatment effects. 2023. R package: https://github.com/mwelz/predmod. In addition, we used R-package VIM (version 6.2.2) and R-package interactionR (version 0.1.7)

For manuscripts utilizing custom algorithms or software that are central to the research but not yet described in published literature, software must be made available to editors and reviewers. We strongly encourage code deposition in a community repository (e.g. GitHub). See the Nature Portfolio guidelines for submitting code & software for further information.

Data

Policy information about availability of data

All manuscripts must include a data availability statement. This statement should provide the following information, where applicable:

- Accession codes, unique identifiers, or web links for publicly available datasets
- A description of any restrictions on data availability
- For clinical datasets or third party data, please ensure that the statement adheres to our policy

The data generated to create the main figures are available in the Source Data file. Access to the deidentified participant data from the National Lung Screening Trial

can be obtained from the United States National Cancer Institute Cancer Data Access Center at https://biometry.nci. nih.gov/cdas for researchers who meet the criteria for access to confidential data. Access to the deidentified participant data from the Dutch-Belgian lung cancer screening trial can be obtained through the NELSON data access board (https://umcgresearch.org/w/nelson-dataset) for researchers who meet the criteria for access to confidential data.

Research involving human participants, their data, or biological material

Policy information about studies with human participants or human data. See also policy information about sex, gender (identity/presentation), and sexual orientation and race, ethnicity and racism.

Reporting on sex and gender

Sex was defined by biological attributes based on self-reported data from the participants in both trials. We refer the reader to the original trial protocols of the NELSON and NLST trials for the reporting of sex-disaggregated data. Analyses were performed by sex in various ways, namely: 1) analyses per sex in the one-variable-at-a-time analyses; 2) sex was included as a confounder in the predictive modelling and random forest approaches. Finally, sensitivity analyses restricted to male participants (as the majority of the NELSON trial consisted of male participants) were performed and yielded similar conclusions as the primary analyses.

Reporting on race, ethnicity, or other socially relevant groupings

Individuals in the NLST provided self-reported information on their race/ethnicity according to the following groupings: Non-Hispanic White, Non-Hispanic Black, Asian, American Indian or Alaskan Native, Native Hawaiian or Pacific Islander, Hispanic. In NELSON, no information on race/ethnicity was collected. Race/ethnicity was incorporated as a covariate in the models.

Population characteristics

The NELSON trial enrolled individuals between the ages of 50-74, who smoked at least 15 cigarettes per day for ≥25 years or 10 cigarettes per day for ≥30 years and were current smokers or former smokers who quit <10 years ago. The intervention arm received four rounds of computed tomography screening with different intervals: at baseline, year 1 (1-year interval), year 3 (2-year interval) and year 5.5 (2.5-year interval). The participants in the control arm received no screening.
The final analyses included 14,808 NELSON participants (12,429 men, 2,377 women, 2 persons with an unknown sex). The median age of the participants was 58.

The NLST enrolled individuals between the ages of 55-74, who smoked at least 30 pack-years and were current smokers or former smokers who quit <15 years ago. The intervention arm received three rounds of computed tomography screening with a one-year interval each: at baseline, year 1 and year 2. The participants in the control arm received three rounds of chest radiography screening with the same schedule as the intervention arm. The final analyses included 53,405 NLST participants (31,501 men, 21,904 women). The median age of the participants was 60.

Recruitment

In NLST, centers were free to choose their own recruitment methods, as described in: https://pmc.ncbi.nlm.nih.gov/articles/PMC3641889/. In brief, participants were recruited through direct mail, community outreach, and mass media. In NELSON, individuals were recruited through direct mail on the basis of population registries. Both NLST (https://academic.oup.com/jnci/article/102/23/1771/920526) and NELSON (https://www.sciencedirect.com/science/article/pii/S1556086415323856?via%3Dihub) found that their participants were more likely to be younger and have ceased smoking, but were generally representative of the general population meeting their inclusion criteria.

Ethics oversight

The NELSON trial (Netherlands Trial Register number NL580) was approved by the Dutch Minister of Health and the medical ethics committee at each participating site. The NLST (ClinicalTrials.gov number NCT00047385) was approved by the institutional review board at each of the 33 participating medical institutions. Participants in both trials provided written informed consent before randomisation. Access to and use of the NELSON and NLST datasets were conducted in compliance with their respective data use agreements and adhered to applicable data privacy standards.

Note that full information on the approval of the study protocol must also be provided in the manuscript.

Field-specific reporting

Please select the one below that is the best fit for your research. If you are not sure, read the appropriate sections before making your selection.

☒ Life sciences ☐ Behavioural & social sciences ☐ Ecological, evolutionary & environmental sciences

For a reference copy of the document with all sections, see nature.com/documents/nr-reporting-summary-flat.pdf

Life sciences study design

All studies must disclose on these points even when the disclosure is negative.

Sample size

All individuals randomized in the NELSON (n=15,792) and NLST (n=53,454) were considered. After data exclusions (see next section), 14,808 NELSON and 53,405 NLST participants were included in the final analyses. Both NELSON and NLST were sufficiently powered to show lung cancer mortality reductions of 25% and 21% respectively.

Data exclusions

For Belgian NELSON participants, only summarised information on LC incidence and mortality was available. Consequently, they were excluded (NELSON: n=934). Furthermore, inadvertently randomised never-smokers (NELSON: n=17), individuals diagnosed with LC before randomisation (NELSON: n=10), and individuals without known LC incidence dates (NELSON: n=23, NLST: n=47) were excluded.

Replication

The NLST and NELSON trials were performed independently from each other. The models were developed and validated through 30 multiple imputated datasets for both trials. Models developed in NELSON were validated in NLST and vice-versa; overall discrimination for LCM and

screening benefit were similar across methods within both development and validation sets, for both overall LCM and histology-specific LCM. Calibration for relative and absolute benefits were generally good in the development datasets for both methods, but poor in the validation sets

Randomization	In NELSON, randomization by screening center was applied. In NLST, blocked randomization stratified by age, sex, and screening center was applied, with blocks of length six or eight (with order of assignment being random within each block).
Blinding	Blinding was not relevant to our study, as it represents a post-hoc analysis with the aim to evaluate heterogeneity in lung cancer screening effectiveness.

Reporting for specific materials, systems and methods

We require information from authors about some types of materials, experimental systems and methods used in many studies. Here, indicate whether each material, system or method listed is relevant to your study. If you are not sure if a list item applies to your research, read the appropriate section before selecting a response.

Materials & experimental systems

n/a	Involved in the study
☒	☐ Antibodies
☒	☐ Eukaryotic cell lines
☒	☐ Palaeontology and archaeology
☒	☐ Animals and other organisms
☐	☒ Clinical data
☒	☐ Dual use research of concern
☒	☐ Plants

Methods

n/a	Involved in the study
☒	☐ ChIP-seq
☒	☐ Flow cytometry
☒	☐ MRI-based neuroimaging

Clinical data

Policy information about clinical studies
All manuscripts should comply with the ICMJE guidelines for publication of clinical research and a completed CONSORT checklist must be included with all submissions.

Clinical trial registration	NELSON trial (Netherlands Trial Register number NL580). NLST (ClinicalTrials.gov number NCT00047385)
Study protocol	The NELSON study protocol can be accessed at: https://www.nejm.org/doi/suppl/10.1056/NEJMoa1911793/suppl_file/nejmoa1911793_protocol.pdf. The NLST protocol can be accessed at: https://www.nejm.org/doi/suppl/10.1056/NEJMoa1102873/suppl_file/nejmoa1102873_protocol.pdf
Data collection	For NLST participants in the United States were enrolled from August 2002 through April 2004; screening took place from August 2002 through September 2007. For NELSON, participants in the Netherlands and Belgium were recruited and randomised between December 2003 and July 2006. Screening took place from January 2004 through December 2012.For NLST, information on stage and histology was derived through the follow-up of individuals with positive screens, annual status update and medical records, including pathology and tumor staging reports, for all suspected lung cancer. For NELSON, data on cancer diagnosis, histology and stage, vital status, and cause of death were obtained through linkages with the Dutch Center for Genealogic and Heraldic Studies, Statistics Netherlands, and the Dutch Cancer Registry.
Outcomes	In NELSON, lung cancer diagnoses (n=743) and lung cancer deaths (n=400) occurring between randomisation and December 31st 2015, or 10 years of follow-up since randomisation (whichever came first) were considered. In NLST, lung cancer diagnoses (n=2,055) and lung cancer deaths (n=977) occurring between randomisation and 7 years of follow-up were considered.

Plants

Seed stocks	Not applicable
Novel plant genotypes	Not applicable
Authentication	Not applicable
